# A Straightforward Synthesis of Functionalized *cis*-Perhydroisoquinolin-1-ones

**DOI:** 10.3390/molecules24030557

**Published:** 2019-02-03

**Authors:** Federica Arioli, Maria Pérez, Celeste Are, Elies Molins, Joan Bosch, Mercedes Amat

**Affiliations:** 1 Laboratory of Organic Chemistry, Faculty of Pharmacy and Food Sciences, and Institute of Biomedicine (IBUB), University of Barcelona, 08028 Barcelona, Spain; fede.arioli@hotmail.it (F.A.); cele.are@ub.edu (C.A.); joanbosch@ub.edu (J.B.); 2 Department of Nutrition, Food Sciences and Gastronomy, Faculty of Pharmacy and Food Sciences, and Institute of Nutrition and Food Safety (INSA-UB), University of Barcelona, 08921 Santa Coloma de Gramanet, Spain; mariaperez@ub.edu; 3 Institut de Ciència de Materials de Barcelona (ICMAB-CSIC), Campus UAB, 08193 Cerdanyola, Spain; elies.molins@icmab.es

**Keywords:** 5,6-dihydro-2(1*H*)-pyridone, Nazarov reagents, perhydroisoquinoline, Michael addition, stereoselectivity

## Abstract

Base-catalyzed annulation reactions of 5,6-dihydro-2(1*H*)-pyridones with Nazarov-type reagents are reported. The effect of the solvent polarity and the concentration of the reagents is studied. The process involves two successive Michael additions and stereoselectively provides functionalized *cis*-perhydroisoquinolin-1-ones.

## 1. Introduction

Nitrogen heterocycles exhibit a broad range of significant biological and pharmacological activities, and many of them have been developed as therapeutic drugs [1,2]. 

**Figure 1:**
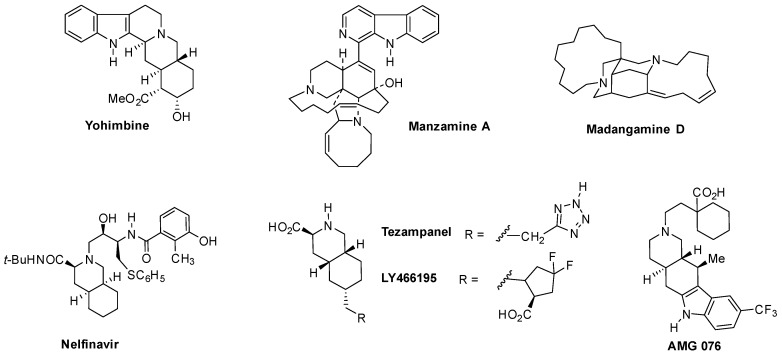
Hydroisoquinoline-containing bioactive natural products and synthetic compounds.

In particular, the partially or totally reduced isoquinoline ring system is present in a large number of biologically active natural products (such as the alkaloids of the yohimbine [3,4], manzamine [5], and madangamine [6] groups) and medicinally useful synthetic compounds (e.g., the HIV protease inhibitors saquinavir and nelfinavir [7,8], the antimigraine drugs tezampanel and LY466195 [9], and the antiobesity agent AMG 076 [10]) (Figure 1).

Together with the intramolecular Diels−Alder cyclization of suitable azatrienes [11,12], one of the most straightforward approaches for the construction of the hydroisoquinoline ring system involves the generation of the carbocyclic ring by an annulation reaction from appropriate 5,6-dihydro-2(1*H*)-pyridone derivatives. The latter strategy was developed some years ago in our laboratory using the classical Diels−Alder methodology with a variety of dienes [13]. Bearing in mind that Nazarov reagents (γ,δ-unsaturated β-keto esters) are able to participate in double Michael addition reactions with α,β-unsaturated carbonyl derivatives, we envisaged an alternative annulation procedure to directly access functionalized hydroisoquinolines from 5,6-dihydro-2(1*H*)-pyridones.

Nazarov reagents are versatile annulating agents, extensively used in a variety of Robinson-type and double Michael addition annulations [14,15,16]. In the former, the reagent sequentially acts as an electrophilic Michael acceptor and as a nucleophile to promote an aldol condensation. In the latter, however, it successively acts as a nucleophilic Michael donor and an electrophilic Michael acceptor, a reactivity pattern that has been successfully applied to assemble pentacyclic yohimbine-type derivatives from unsaturated indolo[2,3-*a*]quinolizidine-derived lactams [17,18,19].

## 2. Results and Discussion

Compounds **6a**,**b**,**c** and **8**, which incorporate an additional phenylsulfonyl or ethoxycarbonyl activating electron-withdrawing group conjugated to the carbon-carbon double bond, were selected as the starting dihydropyridones.

**Scheme 1:**
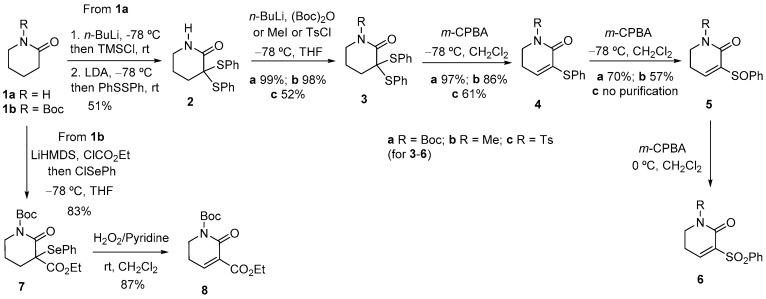
Preparation of the starting unsaturated lactams.

Lactams **6a**,**b**, bearing an easily removable phenylsulfonyl group, were prepared in acceptable overall yield from 2-piperidone (**1a**) by bis-sulfenylation, followed by reaction with either (Boc)_2_O or MeI, and subsequent stepwise *m*-CPBA oxidation of the resulting *N*-substituted piperidones **3a**,**b** via unsaturated sulfenyl and sulfinyl derivatives **4a**,**b** and **5a**,**b**. A similar reaction sequence from *N*-tosyl-2-piperidone **3c** led to unsaturated lactam **6c** in low overall yield. Due to their instability, lactams **6a**,**b**,**c** were used in the annulation step without purification. In turn, ethoxycarbonyl lactam **8** was prepared in high yield from *N*-Boc-2-piperidone (**1b**) via seleno derivative **7**, as outlined in Scheme 1.

To study the annulation reactions, we initially used unsaturated lactam **6a** and the Nazarov reagent **9** [14], which incorporates a methyl group at the terminal olefinic carbon, in the presence of Cs_2_CO_3_, the most commonly used base for the generation of Nazarov enolates [20]. Reagent **9** has been used extensively by Deslongchamps to generate *cis*-decalin derivatives [20,21]. The reaction was carried out at room temperature, using an excess (6 equiv.) of Cs_2_CO_3_ in CH_2_Cl_2_ at different concentrations (from 50 mM to 5 mM). In all cases, the double Michael addition reaction occurred satisfactorily, although with only moderate stereoselectivity, to give 3:1 C-8 stereoisomeric mixtures of *cis*-hydroisoquinolones **10a** (*cis* Me/SO_2_Ph) and **10b** (*trans* Me/SO_2_Ph), the chemical yield increasing (79% yield at 5 mM) with the dilution (Table 1, entries 1–3). Trace amounts of the monoaddition product **11** were detected by NMR, indicating that the annulation involves two successive Michael addition reactions. In contrast, when acetonitrile was used as the solvent the yield was very low (entry 4). Remarkably, the use of KF as the base in a polar solvent such as methanol (entry 5) resulted in a reversal of the stereoselectivity, leading to a mixture of bicyclic lactams **10a** and **10b**, in which the *trans* Me/SO_2_Ph isomer predominated (56% yield, ratio 1:2). The influence of the solvent polarity on the stereoselectivity of annulation reactions of **9** with β-keto esters has previously been observed [21]. The facial selectivity when using Cs_2_CO_3_ as the base can be attributed to the coordination of the Cs^+^ cation to the oxygen atoms of both the Nazarov reagent and the starting lactam [19].

**Figure 2:**
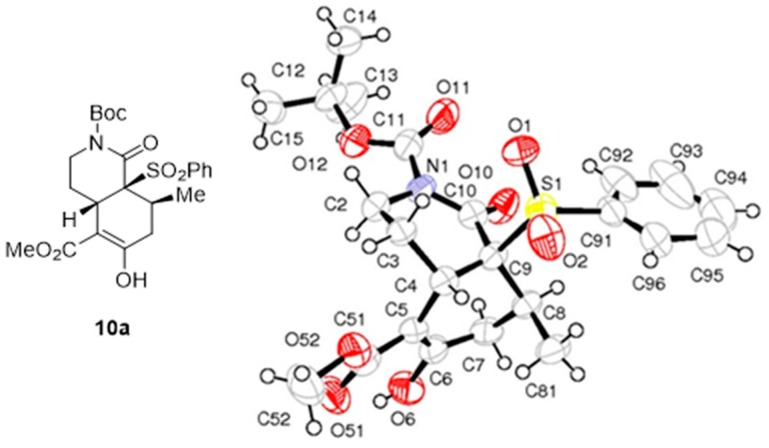
ORTEP plot of the X-ray structure of bicyclic lactam **10a**.

The relative Me/SO_2_Ph *cis* configuration of bicyclic lactam **10a** was unambiguously established by X-ray crystallographic analysis (Figure 2). 

Unsaturated lactam **6b** behaved similarly to lactam **6a** in the annulation reaction with Nazarov reagent **9**, although the yields were slightly lower, probably due to the lower electrophilicity of the Michael acceptor as a consequence of the absence of an electron-withdrawing group on the piperidone nitrogen. *cis*-Hydroisoquinolone **12a**, with a *cis* Me/SO_2_Ph relationship, was stereo-selectively formed (4:1 **12a**/**12b** ratio) when the reaction was performed in CH_2_Cl_2_ solution using Cs_2_CO_3_ as the base (Table 2, entries 1 and 2), the yield once again being higher with increasing dilution (40% yield at 5 mM). As before, a reversal of the stereoselectivity was observed and the *trans* Me/SO_2_Ph isomer predominated when using polar solvents, either MeOH in the presence of KF (1:4 ratio; entry 3) or DMF in the presence of Cs_2_CO_3_ (1:5 ratio; entry 4). The Me/SO_2_Ph *cis* relationship of the adducts **10a** and **12a** was maintained unchanged after an additional treatment (20 h, rt) with Cs_2_CO_3_ in CH_2_Cl_2_ or KF in MeOH, thus suggesting the non-reversibility of the cyclization step.

**Figure 3:**
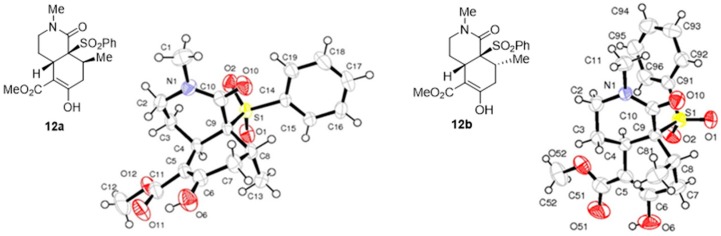
ORTEP plots of the X-ray structures of bicyclic lactams **12a** and **12b**.

The relative configuration of both hydroisoquinolones, **12a** and **12b**, was unambiguously established by X-ray crystallographic analysis (Figure 3).

**Scheme 2:**
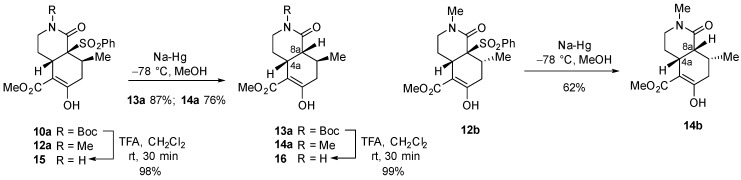
Desulfonylation reactions and removal of the *N*-Boc protecting group.

The activating phenylsulfonyl group of bicyclic lactams **10a**, **12a**, and **12b** was stereoselectively removed, with retention of configuration, by treatment with sodium amalgam [22] to give the respective *cis*-hydroisoquinolones **13a**, **14a**, and **14b** (Scheme 2). Alternatively, removal of the *N*-Boc protecting group of hydroisoquionolones **10a** and **13a** quantitatively afforded the potentially useful *N*-unsubstituted derivatives **15** and **16**, respectively. The *cis* ring function was evident from the observation of a positive NOE effect between the 4a and 8a methine protons.

To expand the scope of the methodology and access hydroisoquinolones lacking the methyl substituent on the B ring, we decided to study double Michael annulations using the silylated Nazarov reagent **17** [17], which constitutes a stable synthetic equivalent of the original Nazarov reagent (methyl or ethyl 3-oxo-4-pentenoate) [15] that avoids the polymerization problems associated with the latter under basic conditions. 

As expected, the Cs_2_CO_3_-promoted annulation of the Nazarov reagent **17** with unsaturated lactam **6a** under the usual reaction conditions (5 mM in CH_2_Cl_2_ as the solvent) stereoselectively afforded *cis*-hydroisoquinolone **18a**, in which protodesilylation had occurred, in acceptable yield (Table 3, entry 1). The yield was not improved by increasing the excess of reagent and base (entry 2) and was lower when operating at a higher concentration (entry 3) or when using KF in MeOH as the solvent (entry 4). Similar moderate yields were obtained in the generation of *cis*-hydroisoquinolones **18b** and **18c** from lactams **6b** (entries 5–7) and **6c** (entry 8) under a variety of conditions.

**Scheme 3:**
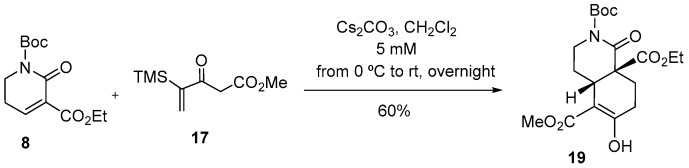
Reaction of unsaturated lactam **8** with the silylated Nazarov reagent **17**.

Remarkably, the yield of the annulation with the silylated Navarov reagent **17** was higher when using unsaturated lactam **8**, which incorporates an ester group as an additional activating substituent. Operating under the previously optimized reaction conditions, *cis*-hydroisoquinolone **19** was obtained in 60% yield (Scheme 3).

In conclusion, base-promoted annulation reactions of Nazarov reagents **9** and **17** with 5,6-dihydro-2(1*H*)-pyridones bearing an additional activating electron-withdrawing group α to the lactam carbonyl constitute a straightforward procedure for the stereoselective synthesis of highly substituted *cis*-hydroisoquinolin-2-ones. In the reactions with the methyl-substituted reagent **9**, leading to 8-substituted derivatives, the use of Cs_2_CO_3_ in CH_2_Cl_2_ leads to *cis*-hydroisoquinolones with a *cis* 8-Me/8a-SO_2_Ph relationship as the major stereoisomers. The stereoselectivity is reversed in a polar solvent such as DMF or when the annulation is performed using KF in MeOH.

The methodology developed here provides access to polyfunctionalized bicyclic scaffolds with potential use as precursors of bioactive hydroisoquinoline-containing natural products and synthetic derivatives.

## 3. Materials and Methods

### 3.1. General Information

All air sensitive manipulations were carried out under a dry argon or nitrogen atmosphere. THF and CH_2_Cl_2_ were dried using a column solvent purification system. Analytical thin-layer chromatography was performed on SiO_2_ (silica gel 60A 35–70 μm, Carlo Erba, Val de Reuil Cedex, France), and the spots were located with 1% aqueous KMnO_4_. Chromatography refers to flash chromatography and was carried out on SiO_2_ (SDS silica gel 60 ACC, 35–75 mm, 230-240 mesh ASTM). NMR spectra were recorded at 300 or 400 MHz (^1^H) and 100.6 MHz (^13^C), and chemical shifts are reported in δ values downfield from TMS or relative to residual chloroform (7.26 ppm, 77.0 ppm) as an internal standard. Data are reported in the following manner: chemical shift, multiplicity, coupling constant (J) in hertz (Hz), integrated intensity, and assignment (when possible). Assignments and stereochemical determinations are given only when they are derived from definitive two-dimensional NMR experiments (HSQC-COSY). IR spectra were performed in an Avatar 320 FT-IR spectrophotometer (Thermo Nicolet, Madison, WI, USA) and only noteworthy IR absorptions (cm^−1^) are listed. High resolution mass spectra (HMRS; LC/MSD TOF, Agilent Technologies, Santa Clara, CA, USA) were performed by Centres Científics i Tecnològics de la Universitat de Barcelona. 

### 3.2. Preparation of the Starting Unsaturated Lactams

*3,3-Bis(phenylthio)-2-piperidone* (**2**). *n*-BuLi (25.5 mL of a 1.6 M solution in hexane, 40.8 mmol) was added under an argon atmosphere at −78 °C to a solution of *δ*-valerolactam (**1a**, 4 g, 40.82 mmol) in anhydrous THF (100 mL). The solution was stirred for 30 minutes, and then TMSCl (5.2 mL, 40.8 mmol) was added. The solution was stirred at room temperature for 30 minutes, and then re-cooled to −78 °C. LDA (45 mL, 2 M solution in THF/heptane/ethylbenzene, 89.8 mmol) was added, and the solution was stirred for 30 minutes. Then, PhSSPh (19.5 g, 89.8 mmol) in anhydrous THF (50 mL) was added, and the resulting solution was stirred at room temperature for 1 h. After this time, NH_4_Cl was added, and the organic layer was extracted with EtOAc. The combined organic extracts were dried over anhydrous MgSO_4_, filtered, and concentrated under reduced pressure. Flash chromatography (9:1 hexane-EtOAc) of the residue gave lactam **2** (6.61 g, 51% yield): IR (ATR Pike) ν (cm^−1^): 3260 (NH), 1661 (CO); ^1^H-NMR (400 MHz, CDCl_3_): *δ* = 1.72-1.90 (m, 2H, H-5), 1.97 (t, *J* = 6.2 Hz, 2H, H-4), 3.18 (dt, *J* = 2.4, 6.0 Hz, 2H, H-6), 6.26 (br. s, 1H, NH), 7.32–7.34 (m, 6H, H_AR_), 7.63–7.65 (m, 4H, H_AR_); HRMS (ESI) calcd for [C_17_H_18_NOS_2_ + H^+^]: 316.0824, found: 316.062714b (detailed data in supplementary materials).

*3,3-Bis(phenylthio)-1-(tert-butoxycarbonyl)-2-piperidone* (**3a**). *n*-BuLi (6.1 mL of a 1.4 M solution in hexane, 8.54 mmol) was added under an argon atmosphere at −78 °C to a solution of lactam **2** (2.25 g, 7.12 mmol) in anhydrous THF (100 mL), and the resulting solution was stirred for 1 h. Then, Boc_2_O (2.30 g, 10.68 mmol) was added, and the mixture was stirred at −78 °C for 1 h. After this time, saturated NH_4_Cl was added, and the mixture was extracted with EtOAc. The combined organic extracts were dried over Na_2_SO_4_ and filtered, and the solvent was removed under reduced pressure. Flash chromatography (9:1 hexane-EtOAc) of the residue gave *N*-Boc derivative **3a** (2.92 g, 99% yield): IR (ATR Pike) ν (cm^−1^): 1717 (CO); ^1^H-NMR (400 MHz, CDCl_3_): *δ* = 1.53 [s, 9H, (CH_3_)_3_C], 1.94 (m, 2H, H-5), 2.05 (m, 2H, H-4), 3.60 (t, *J* = 6.1 Hz, 2H, H-6), 7.33–7.42 (m, 6H, H_AR_), 7.68 (m, 4H, H_AR_); ^13^C-NMR (400 MHz, CDCl_3_): *δ* = 20.1 (C-5), 28.0 [(*C*H_3_)_3_C], 35.1 (C-4), 46.7 (C-6), 68.6 (C-3), 83.2 [(CH_3_)_3_*C*], 128.7 (4CH_AR_), 129.6 (2CH_AR_), 130.9 (2C_AR_), 136.8 (4CH_AR_), 153.8 (CO), 168.3 (CO); HRMS (ESI) calcd for [C_22_H_25_NO_3_S_2_ + Na^+^]: 438.1168, found: 438.117.

*3,3-Bis(phenylthio)-1-methyl-2-piperidone* (**3b**). *n*-BuLi (8.54 mL of a 1.6 M solution in hexane, 13.67 mmol) was added under an argon atmosphere at −78 °C to a solution of lactam **2** (3.60 g, 11.39 mmol) in anhydrous THF (200 mL), and the resulting mixture was stirred at −78 °C for 1 h. Then, MeI (1.06 mL, 17.09 mmol) was added, and the solution was stirred at room temperature for 1 h. The reaction was quenched by addition of saturated NH_4_Cl, and the mixture was extracted with EtOAc. The combined organic extracts were dried over Na_2_SO_4_, filtered, and concentrated under reduced pressure. Flash chromatography (9:1 hexane-EtOAc) of the residue afforded *N*-methyl derivative **3b** (3.7 g, 98% yield): IR (ATR Pike) ν (cm^−1^): 1642 (NCO); ^1^H-NMR (400 MHz, CDCl_3_): *δ* = 1.86–1.91 (m, 2H, H-5), 1.97–1.99 (m, 2H, H-4), 2.92 (s, 3H, CH_3_), 3.16 (t, *J* = 6.4 Hz, 2H, H-6), 7.31–7.38 (m, 6H, H_AR_), 7.63–7.65 (m, 4H, H_AR_); ^13^C-NMR (100.6 MHz, CDCl_3_): *δ* = 19.6 (C-5), 34.0 (C-4), 35.8 (CH_3_), 50.1 (C-6), 66.0 (C-3), 128.6 (4CH_AR_), 129.3 (2CH_AR_), 131.6 (2C_AR_), 136.6 (4CH_AR_); HRMS (ESI) calcd for [C_18_H_19_NOS_2_ + H^+^]: 330.0981, found: 330.0977.

*3,3-Bis(phenylthio)-1-(p-toluenesulfonyl)-2-piperidone* (**3c**). *n*-BuLi (1.2 mL of a 1.6 M solution in hexane, 1.9 mmol) was added at −78 °C to a solution of lactam **2** (500 mg, 1.58 mmol) in anhydrous THF (25 mL), and the resulting mixture was stirred at −78 °C for 1 h. Then, TsCl (452 mg, 2.37 mmol) was added, and the solution was stirred at room temperature overnight. The reaction was quenched by addition of saturated NH_4_Cl, and the mixture was extracted with EtOAc. The combined organic extracts were dried over Na_2_SO_4_, filtered, and concentrated under reduced pressure. Flash chromatography (9:1 hexane-EtOAc) afforded *N*-tosyl derivative **3c** (264 mg, 52% yield): IR (ATR Pike) ν (cm^−1^): 1683 (NCO), 1169 (SO_2_); ^1^H-NMR (400 MHz, CDCl_3_): *δ* = 1.55 (s, 2H, H-5), 2.02 (s, 2H, H-4), 2.49 (s, 3H, CH_3_), 3.46 (br. s, 2H, H-6), 7.19–7.40 (m, 12H, H_AR_), 7.93 (d, *J* = 8.4 Hz, 2H, H_AR_).

*1-(tert-Butoxycarbonyl)-3-(phenylthio)-5,6-dihydro-2(1H)-pyridone* (**4a**). *m*-Chloroperbenzoic acid (1.09 g, technical grade 70%, 766 mg pure oxidant, 6.33 mmol) in CH_2_Cl_2_ (100 mL) was added to a solution of lactam **3a** (2.64 g, 6.33 mmol) in CH_2_Cl_2_ (200 mL) at −78 °C. The solution was stirred at this temperature for 2 h and then for 30 minutes at room temperature. After this time, a saturated solution of NaHCO_3_ was added, the mixture was extracted with CH_2_Cl_2_, and the organic solvent was removed under reduced pressure. The crude product was purified by flash chromatography (95:5 hexane-EtOAc) to afford unsaturated lactam **4a** (1.88 g, 97% yield): IR (ATR Pike) ν (cm^−1^): 1762 (CO), 1715 (CO); ^1^H-NMR (300 MHz, CDCl_3_): *δ* = 1.54 [s, 9H, (CH_3_)_3_C], 2.39 (td, *J* = 6.6, 4.8 Hz, 2H, H-5), 3.84 (t, *J* = 6.6 Hz, 2H, H-6), 6.08 (t, *J* = 4.8 Hz, 1H, H-4), 7.33–7.41 (m, 2H, H_AR_), 7.47–7.50 (m, 2H, H_AR_), 7.66–7.70 (m, 1H, H_AR_); ^13^C-NMR (100.6 MHz, CDCl_3_): *δ* = 25.3 (C-5), 28.0 [(*C*H_3_)_3_C], 43.7 (C-6), 83.4 [(CH_3_)_3_*C*], 128.8 (CH_AR_), 129.5 (2CH_AR_), 131.0 (C-3, C_AR_), 134.8 (2CH_AR_), 135.5 (C-4), 152.8 (CO), 161.7 (CO); HRMS (ESI) calcd for [C_16_H_19_NO_3_S + Na^+^]: 328.0978, found: 328.0975. 

*1-Methyl-3-(phenylthio)-5,6-dihydro-2(1H)-pyridone* (**4b**). Operating as described for the preparation of compound **4a**, from a solution of lactam **3b** (280 mg, 0.85 mmol) in CH_2_Cl_2_ (20 mL) and *m*-CPBA (210 mg, technical grade 70%, 0.85 mmol) in CH_2_Cl_2_ (20 mL), unsaturated lactam **4b** was obtained (160 mg, 86% yield) as a yellow foam: ^1^H-NMR (400 MHz, CDCl_3_, COSY, g-HSQC): *δ* = 2.34 (td, *J* = 7.2, 4.8 Hz, 2H, H-5), 3.04 (s, 1H, CH_3_), 3.40 (t, *J* = 7.2 Hz, 2H, H-6), 5.81 (t, *J* = 4.8 Hz, 1H, H-4), 7.35–7.39 (m, 3H, H_AR_), 7.48–7.50 (m, 2H, H_AR_); ^13^C-NMR (100.6 MHz, CDCl_3_): *δ* = 24.6 (C-5), 34.9 (CH_3_), 47.6 (C-6), 128.5 (CH_AR_), 129.4 (2CH_AR_), 130.8 (C-4), 132.0, 134.4 (C-3, C_AR_), 134.6 (2CH_AR_), 162.9 (CO).

*3-(Phenylthio)-1-(p-toluenesulfonyl)-5,6-dihydro-2(1H)-pyridone* (**4c**). Operating as described for the preparation of compound **4a**, from a solution of lactam **3c** (264 mg, 0.58 mmol) in CH_2_Cl_2_ (15 mL) and *m*-CPBA (142 mg, technical grade 70%, 0.58 mmol) in CH_2_Cl_2_ (15 mL), unsaturated lactam **4c** was obtained (123 mg, 61% yield) as a yellow foam: IR (ATR Pike) ν (cm^−1^): 1683 (CO), 1166 (SO_2_); ^1^H-NMR (400 MHz, CDCl_3_, COSY, g-HSQC): *δ* = 2.43 (s, 3H, CH_3_), 2.48 (td, *J* = 6.3, 4.8 Hz, 2H, H-5), 4.04 (t, *J* = 6.3 Hz, 2H, H-6), 6.08 (t, *J* = 4.8 Hz, 1H, H-4), 7.30–7.37 (m, 7H, H_AR_), 7.93 (d, *J* = 8.4 Hz, 2H, H_AR_); ^13^C-NMR (100.6 MHz, CDCl_3_): *δ* = 21.6 (CH_3_), 25.9 (C-5), 44.1 (C-6), 128.7 (CH_AR_), 128.9 (CH_AR_), 129.4 (CH_AR_), 129.6 (CH_AR_), 130.7 (C_AR_), 134.5 (CH_AR_), 135.6 (C-3, C_AR_), 136.2 (C-4), 144.9 (CH_AR_), 161.1 (NCO); HRMS (ESI) calcd for [C_18_H_17_NO_3_S_2_ + H^+^]: 360.0723, found: 360.0720.

*1-(tert-Butoxycarbonyl)-3-(phenylsulfinyl)-5,6-dihydro-2(1H)-pyridone* (**5a**). *m*-Chloroperbenzoic acid (1.51 g, technical grade 70%, 1.06 g pure oxidant, 6.14 mmol) in CH_2_Cl_2_ (100 mL) was added to a solution of phenylthio derivative **4a** (1.884 g, 6.14 mmol) in CH_2_Cl_2_ (200 mL) at −78 °C. The solution was stirred at this temperature for 1.5 h. Then, a saturated solution of NaHCO_3_ was added, the mixture was extracted with CH_2_Cl_2_, and the organic solvent was removed under reduced pressure. The crude product was purified by flash chromatography (95:5 hexane-EtOAc) to afford sulfinyl derivative **5a** (1.39 g, 70% yield): IR (ATR Pike) ν (cm^−1^): 1762 (CO), 1719 (CO), 1099, 1049 (SO); ^1^H-NMR (400 MHz, CDCl_3_, COSY, g-HSQC): *δ* = 1.51 [s, 9H, (CH_3_)_3_C], 2.55–2.75 (m, 2H, H-5), 3.59 (ddd, *J* = 13.2, 10.4, 5.2 Hz, 1H, H-6), 4.05 (dtd, *J* = 13.2, 5.2, 1.1 Hz, H-6), 7.45-7.47 (m, 3H, H_AR_), 7.57 (td, *J* = 3.6, 1.2 Hz, 1H, H-4), 7.79–7.81 (m, 2H, H_AR_); ^13^C-NMR (100.6 MHz, CDCl_3_): *δ* = 24.9 (C-5), 27.9 [(*C*H_3_)_3_C], 43.2 (C-6), 83.8 [(CH_3_)_3_*C*)], 125.6 (2CH_AR_), 129.1 (2CH_AR_), 131.3 (CH_AR_), 142.3 (C-4), 142.6, 143.7 (C_AR_, C-3), 151.9 (CO), 160.2 (CO); HRMS (ESI) calcd for [C_16_H_19_NO_4_S + Na^+^]: 344.0927, found: 344.0927. 

*1-(Methyl)-3-(phenylsulfinyl)-5,6-dihydro-2(1H)-pyridone* (**5b**). Operating as described for the preparation of compound **5a**, from a solution of phenylthio derivative **4b** (163 mg, 0.74 mmol) in CH_2_Cl_2_ (15 mL) and *m*-CPBA (183 mg, technical grade 70%, 0.74 mmol) in CH_2_Cl_2_ (20 mL), sulfinyl derivative **5b** was obtained (100 mg, 57% yield): IR (ATR Pike) ν (cm^−1^): 1652 (CO), 1043 (SO); ^1^H-NMR (400 MHz, CDCl_3_, COSY, g-HSQC): *δ* = 2.53–2.74 (2m, 2H, H-5), 2.90 (s, 3H, CH_3_), 3.38–3.46 (dd, *J* = 8.8, 6.8 Hz, 2H, H-6), 7.31 (t, *J* = 4.8 Hz, 1H, H-4), 7.44–7.45 (m, 3H, H_AR_), 7.78–7.81 (m, 2H, H_AR_); ^13^C-NMR (100.6 MHz, CDCl_3_): *δ* = 24.0 (C-5), 33.7 (CH_3_), 46.8 (C-6), 125.5 (2CH_AR_), 128.8 (2CH_AR_), 131.0 (CH_AR_), 138.1 (C-4), 140.6, 143.8 (C_AR_, C-3), 160.1 (NCO); HRMS (ESI) calcd for [C_12_H_13_NO_2_S + H^+^]: 236.074, found: 236.0741.

*3-(Phenylsulfinyl)-1-(p-toluenesulfonyl)-5,6-dihydro-2(1H)-pyridone* (**5c**). Operating as described for the preparation of compound **5a**, from a solution of phenylthio derivative **4c** (55 mg, 0.12 mmol) in CH_2_Cl_2_ (3 mL) and *m*-CPBA (30 mg, technical grade 70%, 0.12 mmol) in CH_2_Cl_2_ (3 mL), sulfinyl derivative **5c** was obtained. This compound was used in the next step without further purification: IR (ATR Pike) ν (cm^−1^): 1683, (NCO), 1362 (SO2), 1170 (SO); ^1^H-NMR (400 MHz, CDCl_3_, COSY, g-HSQC): *δ* = 2.43 (s, 3H, CH_3_), 2.67-2.81 (m, 2H, H-5), 3.78–3.86 (m, 1H, H-6), 4.11–4.20 (m, 1H, H-6), 7.27 (d, *J* = 7.6 Hz, 2H, H_AR_), 7.36–7.41 (m, 3H, H_AR_), 7.52 (dd, *J* = 4.4, 3.6 Hz, 1H, H-4), 7.63–7.66 (m, 2H, H_AR_), 7.82 (d, *J* = 8.0 Hz, 2H, H_AR_); ^13^C-NMR (100.6 MHz, CDCl_3_): *δ* = 21.6 (CH_3_), 25.3 (C-5), 43.8 (C-6), 125.1 (2CH_AR_), 128.3 (2CH_AR_), 129.1 (2CH_AR_), 129.4 (2CH_AR_), 131.3 (CH_AR_), 135.1 (C_AR_), 142.8 (C-4), 143.2, 145.1 (C_AR_, C-3), 159.7 (NCO).

*1-(tert-Butoxycarbonyl)-3-(phenylsulfonyl)-5,6-dihydro-2(1H)-pyridone* (**6a**). *m*-Chloroperbenzoic acid (1.06 mg, technical grade 70%, 745 mg pure oxidant, 4.32 mmol) in CH_2_Cl_2_ (150 mL) was added to a solution of sulfinyl derivative **5a** (1.39 g, 4.32 mmol) in CH_2_Cl_2_ (60 mL) at −78 °C. The solution was stirred at room temperature for 6 h. Then, a saturated solution of NaHCO_3_ was added, the mixture was extracted with CH_2_Cl_2_, and the organic solvent was removed under reduced pressure to afford sulfonyl derivative **6a**, which was used in the next step without purification: IR (ATR Pike) ν (cm^−1^): 1721 (CO), 1689 (CO), 1446 (SO_2_), 1143 (SO_2_); ^1^H-NMR (400 MHz, CDCl_3_, COSY, g-HSQC): *δ* = 1.49 [s, 9H, (CH_3_)_3_C], 2.67 (td, *J* = 6.0, 4.0 Hz, 2H, H-5), 3.87 (t, *J* = 6.8 Hz, 2H, H-6), 7.51–7.53 (m, 2H, H_AR_), 7.60 (t, *J* = 7.6 Hz, 1H, H_AR_), 8.05 (dm, *J* = 7.2 Hz, 2H, H_AR_), 8.08 (t, *J* = 4.4 Hz, 1H, H-4); ^13^C-NMR (100.6 MHz, CDCl_3_): *δ* = 25.0 (C-5), 27.9 [(CH_3_)_3_C], 42.9 (C-6), 84.2 [(CH_3_)_3_C], 128.7 (2CH_AR_), 129.3 (2CH_AR_), 133.5 (CH_AR_), 138.7, 139.5 (C-3, C_AR_), 151.4 (CO), 152.2 (C-4), 157.8 (CO); HRMS (ESI) calcd for [C_16_H_19_NO_5_S + H^+^]: 338.1057, found: 338.1066.

*1-Methyl-3-(phenylsulfonyl)-5,6-dihydro-2(1H)-pyridone* (**6b**). Operating as described for the preparation of compound **6a** (reaction time 20 h), from a solution of sulfinyl derivative **5b** (100 mg, 0.42 mmol) in CH_2_Cl_2_ (10 mL) and *m*-CPBA (104 mg, technical grade 70%, 0.42 mmol) in CH_2_Cl_2_ (10 mL), sulfonyl derivative **6b** was obtained. This compound was used in the next step without purification: IR (ATR Pike) ν (cm^−1^): 1662 (NCO), 1306 (SO_2_), 1151 (SO_2_); ^1^H-NMR (400 MHz, CDCl_3_, COSY, g-HSQC): *δ* = 2.67 (td, *J* = 7.2, 4.4 Hz, 2H, H-5), 2.93 (s, 3H, CH_3_), 3.46 (t, *J* = 7.2 Hz, 2H, H-6), 7.51 (tm, *J* = 7.6 Hz, 2H, H_AR_), 7.57 (m, 1H, H_AR_), 7.87 (t, *J* = 4.4 Hz, 1H, H-4), 8.05 (dm, *J* = 7.6 Hz, 2H, H_AR_); ^13^C-NMR (100.6 MHz, CDCl_3_): *δ* = 24.3 (C-5), 34.4 (CH_3_), 46.6 (C-6), 128.6 (2CH_AR_), 129.1 (2CH_AR_), 133.4 (CH_AR_), 140.0 (C-3, C_AR_), 148.4 (C-4), 159.0 (CO); HRMS (ESI) calcd for [C_12_H_13_NO_3_S + H^+^]: 252.0689, found: 252.0679. 

*3-(Phenylsulfonyl)-1-(p-toluenesulfonyl)-5,6-dihydro-2(1H)-pyridone* (**6c**). Operating as described for the preparation of compound **6a** (reaction time 20 h), from a solution of sulfinyl derivative **5c** (45 mg, 0.12 mmol) in CH_2_Cl_2_ (6 mL) and *m*-CPBA (30 mg, technical grade 70%; 21 mg pure oxidant, 0.12 mmol) in CH_2_Cl_2_ (6 mL), sulfonyl derivative **6c** was obtained. This compound was used in the next step without purification: IR (ATR Pike) ν (cm^−1^): 1695 (NCO), 1362 (SO_2_), 1157 (SO_2_); ^1^H-NMR (400 MHz, CDCl_3_, COSY, g-HSQC): *δ* = 2.42 (s, 1H, CH_3_), 2.79 (td, *J* = 6.4, 4.4 Hz, 2H, H-5), 4.04 (t, *J* = 6.0 Hz, 2H, H-6), 7.29 (d, *J* = 8.0 Hz, 2H, H_AR_), 7.47–7.51 (m, 2H, H_AR_), 7.57 (tt, *J* = 7.6, 1.2 Hz, 1H, H_AR_), 7.85 (d, *J* = 8.0 Hz, 2H, H_AR_), 7.96 (d, *J* = 8.0 Hz, 2H, H_AR_), 8.06 (t, *J* = 4.4 Hz, 1H, H-4); ^13^C-NMR (100.6 MHz, CDCl_3_): *δ* = 21.6 (CH_3_), 25.7 (C-5), 43.5 (C-6), 128.4 (2CH_AR_), 128.8 (2CH_AR_), 129.1 (2CH_AR_), 129.6 (2CH_AR_), 133.7 (CH_AR_), 134.9 (C_AR_), 137.8 (CH_AR_), 139.0 (C-3), 145.3 (C_AR_), 153.2 (C-4), 157.2 (NCO).

*1-(tert-Butoxycarbonyl)-3-(ethoxycarbonyl)-3-(phenylselenyl)-2-piperidone* (**7**). Lithium bis(trimethyl silyl)amide (11.1 mL of a 1 M solution in THF, 11.1 mmol) was slowly added at −78 °C under an argon atmosphere to a solution of lactam **1b** (1.0 g, 5.05 mmol) in anhydrous THF (100 mL), and the solution was stirred for 1 h. Then, ethyl chloroformate (532 μl, 5.56 mmol) and, after 1 h of continuous stirring at −78 °C, a solution of PhSeCl (1.48 g, 7.58 mmol) in anhydrous THF (10 mL) were added to the solution. The mixture was stirred for a further 1 h and poured into saturated aqueous NH_4_Cl. The resulting mixture was extracted with EtOAc, and the combined organic extracts were dried, filtered, and concentrated under reduced pressure. Flash chromatography (8:2 hexane-EtOAc) of the resulting oil gave the seleno derivative **7** (1.79 g, 83% yield) as a yellow foam: IR (ATR Pike) ν (cm^−1^): 1718 (CO); ^1^H-NMR (300 MHz, CDCl_3_): *δ* = 1.27 (t, *J* = 7.2 Hz, 3H, CH_2_C*H*_3_), 1.54 [s, 9H, (CH_3_)_3_C], 1.73 (m, 1H, H-5), 1.84 (m, 1H, H-5), 2.01 (ddd, *J* = 13.8, 10.2, 5.7 Hz, 1H, H-4), 2.28 (dt, *J* = 13.8, 6.0 Hz, 1H, H-4), 3.52 (m, 1H, H-6), 3.61 (ddd, *J* = 13.2, 8.4, 5.4 Hz, 1H, H-6), 4.22 (qd, *J* = 7.2, 3.3 Hz, 2H, C*H*_2_CH_3_), 7.31 (t, *J* = 7.2, 2H, H_AR_), 7.41 (t, *J* = 7.2 Hz, 1H, H_AR_), 7.65 (t, *J* = 7.2 Hz, 2H, H_AR_); HRMS (ESI) calcd for [C_19_H_25_NO_5_Se + Na^+^]: 450.0787, found: 450.0788.

*1-(tert-Butoxycarbonyl)-3-(ethoxycarbonyl)-5,6-dihydro-2(1H)-pyridone* (**8**). H_2_O_2_ (900 μl, 29.35 mmol) and pyridine (405 μl, 5.03 mmol) were added at room temperature to a solution of seleno lactam **7** (1.79 g, 4.19 mmol) in CH_2_Cl_2_ (300 mL), and the resulting solution was stirred at room temperature for 30 minutes. Water was then added, and the mixture was extracted with CH_2_Cl_2_. The combined organic extracts were dried, filtered, and concentrated under reduced pressure. Flash chromatography (8:2 hexane-EtOAc) of the resulting oil gave unsaturated lactam **8** (981 mg, 87% yield) as a yellow foam: IR (ATR Pike) ν (cm^−1^): 1715 (CO); ^1^H-NMR (400 MHz, CDCl_3_, COSY, g-HSQC): *δ* = 1.33 (t, *J* = 6.8 Hz, 3H, CH_2_C*H*_3_), 1.54 [s, 9H, (CH_3_)_3_C], 2.52 (td, *J* = 6.4, 4.4 Hz, 2H, H-5), 3.88 (t, *J* = 6.4 Hz, 2H, H-6), 4.29 (q, *J* = 6.8 Hz, 2H, C*H*_2_CH_3_), 7.48 (t, *J* =.4 Hz, 1H, H-4); ^13^C-NMR (100.6 MHz, CDCl_3_): *δ* = 14.1 (CH_2_*C*H_3_), 24.7 (C-5), 28.0 [(*C*H_3_)_3_C], 43.0 (C-6), 61.5 (*C*H_2_CH_3_), 83.6 [(CH_3_)_3_*C*], 130.9 (C-3), 148.5 (C-4), 160.0 (CO), 164.0 (CO); HRMS (ESI) calcd for [C_13_H_19_NO_5_ + Na^+^]: 292.1155, found: 292.1166.

### 3.3. General Procedure for the Double Michael Addition Reactions

A solution of unsaturated lactam **6a**,**b**,**c** or **8** (1 equiv.) in anhydrous CH_2_Cl_2_, MeCN or MeOH was added at 0 °C under an argon atmosphere to a solution of the Nazarov reagent (**9** or **15**) and Cs_2_CO_3_ or KF in anhydrous CH_2_Cl_2_, MeCN or MeOH, and the resulting mixture was allowed to warm slowly to room temperature. After 20 h of stirring at room temperature, the mixture was concentrated under reduced pressure. Flash chromatography (9:1 hexane-EtOAc) of the residue afforded the corresponding adduct/s (**10a,b**, **12a,b**, **16a**,**b**,**c** or **17**) as yellow foams (detailed data in supplementary materials).

*cis-2-(tert-Butoxycarbonyl)-6-hydroxy-5-(methoxycarbonyl)-8-methyl-1-oxo-8a-(phenylsulfonyl)-1,2,3,4,4a, 7,8,8a-octahydroisoquinolines***10a** and **10b**. Operating as described above in the general procedure, from unsaturated lactam **6a** (800 mg, 2.28 mmol) in anhydrous CH_2_Cl_2_ (200 mL), Nazarov reagent **9** (1.07 g, 7.5 mmol), and Cs_2_CO_3_ (4.9 g, 15 mmol) in anhydrous CH_2_Cl_2_ (250 mL), *cis*-hydroisoquinolones **10a** (*cis* Me/SO_2_Ph isomer) and **10b** (*trans* Me/SO_2_Ph isomer) were obtained (ratio 3:1, 890 mg, 79% yield). Compound **10a**: IR (ATR Pike) ν (cm^−1^): 1727 (CO), 1654 (CO); ^1^H-NMR (400 MHz, CDCl_3_, COSY, g-HSQC): *δ* = 1.08 (d, *J* = 6.4 Hz, 3H, CH_3_), 1.50 [s, 9H, (CH_3_)_3_C], 1.89 (m, 1H, H-7), 2.32 (dm, *J* = 14.8 Hz, 1H, H-4), 2.40 (m, 2H, H-8, H-7), 2.70 (dddd, *J* = 14.8, 13.6, 5.2, 3.6 Hz, 1H, H-4), 3.23 (td, *J* = 12.8, 4.0 Hz, 1H, H-3), 3.74 (dm, *J* = 12.8 Hz, 1H, H-3), 3.82 (s, 3H, CH_3_O) 3.92 (br. s, 1H, H-4a), 7.49 (td, *J* = 7.6, 2.0 Hz, 2H, H_AR_), 7.63 (td, *J* = 7.6, 1.2 Hz, 1H, H_AR_), 7.96–7.99 (dd, *J* = 7.6, 1.2 Hz, 2H, H_AR_); ^13^C-NMR (100.6 MHz, CDCl_3_): *δ* = 14.0 (CH_3_), 23.3 (C-4), 27.8 [(*C*H_3_)_3_C], 29.3 (C-4a), 30.8 (C-8), 35.0 (C-7), 42.9 (C-3), 51.8 (CH_3_O), 77.4 (C-8a), 83.6 [(CH_3_)_3_*C*], 96.6 (C-5), 128.3 (2CH_AR_), 130.9 (2CH_AR_), 134.1 (CH_AR_), 137.0 (C_AR_), 151.8 (CO), 167.1 (CO), 171.5, 171.6 (CO, C-6); HRMS (ESI) calcd for [C_23_H_29_NO_8_S + Na^+^]: 502.1506, found: 502.1511. Compound **10b**: ^1^H-NMR (400 MHz, CDCl_3_, COSY, g-HSQC): *δ* = 1.06 (d, *J* = 6.8 Hz, 3H, CH_3_), 1.53 [s, 9H, (CH_3_)_3_C], 1.69 (ddd, *J* = 13.2, 3.6, 1H, H-4), 2.17 (d, *J* = 18.4 Hz, 1H, H-7), 2.44 (dd, *J* = 14.0, 2.8 Hz, 1H, H-4), 2.79 (m, 1H, H-8), 3.15 (dd, *J* = 18.0, 6.0 Hz, 1H, H-7), 3.39 (td, *J* = 12.8, 3.2 Hz, 1H, H-3), 3.71 (dd, *J* = 12.8, 1.2, 1H, H-4a), 3.86 (s, 3H, CH_3_O) 3.94 (dt, *J* = 12.4, 2.4 Hz, H-3), 7.54 (t, *J* = 7.2 Hz, 2H, H_AR_), 7.68 (td, *J* = 8.0, 1.2 Hz, 1H, H_AR_), 7.89 (d, *J* = 8.0 Hz, 2H, H_AR_); ^13^C-NMR (100.6 MHz, CDCl_3_): *δ* = 20.4 (CH_3_), 27.7 (C-4), 27.9 [(*C*H_3_)_3_C], 30.1 (C-8), 33.2 (C-7), 33.2 (C-4a), 45.7 (C-3), 51.8 (CH_3_O), 77.9 (C-8a), 83.5 [(CH_3_)_3_*C*], 96.8 (C-5), 128.5 (2CH_AR_), 131.0 (2CH_AR_), 134.3 (CH_AR_), 134.9 (C_AR_), 151.5 (CO), 166.3 (CO), 171.0, 171.8 (CO, C-6).

*cis-6-Hydroxy-5-(methoxycarbonyl)-2,8-dimethyl-1-oxo-8a-(phenylsulfonyl)-1,2,3,4,4a,7,8,8a-octahydroiso- quinolines***12a** and **12b**. Operating as described in the general procedure, from unsaturated lactam **6b** (220 mg, 0.87 mmol) in anhydrous MeOH (160 mL), Nazarov reagent **9** (372 mg, 2.62 mmol), and KF (304 mg, 5.24 mmol) in anhydrous MeOH (10 mL), *cis*-hydroisoquinolones **12a** (*cis* Me/SO_2_Ph isomer) and **12b** (*trans* Me/SO_2_Ph isomer) were obtained (ratio 1:5, 175 mg, 51% yield). Compound **12a**: IR (ATR Pike) ν (cm^−1^): 1646 (CO); ^1^H-NMR (400 MHz, CDCl_3_, COSY, g-HSQC): *δ* = 1.09 (d, *J* = 6.8 Hz, 3H, CH_3_), 1.90 (dd, *J* = 18.5, 2.0 Hz, 1H, H-7), 2.30 (dq, *J* = 17.6, 2.4 Hz, 1H, H-4), 2.39 (dm, *J* = 18.5 Hz, 1H, H-7), 2.48 (m, 1H, H-8), 2.67-2.73 (m, 1H, H-4), 2.96 (s, 3H, CH_3_N), 3.21 (m, 2H, H-3), 3.84 (s, 3H, CH_3_O), 7.53 (t, *J* = 7.6 Hz, 2H, H_AR_), 7.65 (t, *J* = 7.2 Hz, 1H, H_AR_), 8.07 (dd, *J* = 7.6 Hz, 1H, H_AR_), 12.34 (s, 1H, OH); ^13^C-NMR (100.6 MHz, CDCl_3_): *δ* = 13.8 (CH_3_), 23.0 (C-4), 29.6 (C-4a), 30.5 (C-8), 35.3 (C-7), 35.8 (NCH_3_), 46.3 (C-3), 51.8 (CH_3_O), 75.5 (C-8a), 96.6 (C-5), 128.3 (2CH_AR_), 131.0 (2CH_AR_), 133.9 (CH_AR_), 138.0, (C_AR_), 165.0 (CO), 171.9 (C-6, CO); HRMS (ESI) calcd for [C_19_H_23_NO_6_S + H^+^]: 394.1319, found: 394.1323. Compound **12b**: ^1^H-NMR (400 MHz, CDCl_3_, COSY, g-HSQC): *δ* = 1.02 (d, *J* = 7.2 Hz, 3H, CH_3_), 1.69 (qd, *J* = 12.8, 4.4 Hz, 1H, H-4), 2.16 (dd, *J* = 18.0, 1.6 Hz, 1H, H-7), 2.32 (dm, *J* = 12.8 Hz, 1H, H-4), 2.77 (s, 3H, CH_3_N), 3.04 (t, *J* = 6.8 Hz, 1H, H-8), 3.04–3.10 (m, 2H, H-7, H-3), 3.25 (td, *J* = 13.2, 4.0 Hz, 1H, H-3), 3.60 (dd, *J* = 12.8, 4.0 Hz, 1H, H-4a), 3.81 (s, 3H, CH_3_O), 7.51 (t, *J* = 7.6 Hz, 2H, H_AR_), 7.62 (t, *J* = 7.2 Hz, 1H, H_AR_), 7.90 (t, *J* = 7.2 Hz, 1H, H_AR_), 12.2 (s, 1H, OH); ^13^C-NMR (100.6 MHz, CDCl_3_): *δ* = 20.6 (CH_3_), 28.1 (C-4), 29.7 (C-8), 33.2 (C-7), 33.9 (C-4a), 35.8 (CH_3_N), 48.8 (C-3), 51.8 (CH_3_O), 75.8 (C-8a), 97.4 (C-5), 128.3 (CH_AR_), 130.8 (CH_AR_), 133.9 (C-3), 136.7, (C_AR_), 164.9 (CO), 171.1 (C-6), 171.9 (CO).

*cis-2-(tert-Butoxycarbonyl)-6-hydroxy-5-(methoxycarbonyl)-1-oxo-8a-(phenylsulfonyl)-1,2,3,4,4a,7,8,8a-octahydroisoquinoline* (**18a**). Operating as described in the general procedure, from unsaturated lactam **6a** (150 mg, 0.44 mmol) in anhydrous CH_2_Cl_2_ (65 mL), Nazarov reagent **17** (268 mg, 1.33 mmol), and Cs_2_CO_3_ (865 mg, 2.66 mmol) in anhydrous CH_2_Cl_2_ (20 mL), *cis*-hydroisoquinolone **18a** was obtained (96 mg, 47% yield): IR (ATR Pike) ν (cm^−1^): 1733 (C=O), 1653 (NCO); ^1^H-NMR (400 MHz, CDCl_3_, COSY, *g*-HSQC): *δ* = 1.55 [s, 9H, (*C*H_3_)_3_C)], 1.64 (m, 1H, H-4), 2.12–2.21 (m, 3H, 2H-7, H-8), 2.46-2.54 (m, 2H, H-8, H-4), 3.62-3.74 (m, 2H, 2H-3), 3.85 (s, 3H, CH_3_O), 3.96 (dd, *J* = 9.6, 3.6 Hz, H-4a), 7.53 (t, *J* = 7.6 Hz, 2H, H_AR_), 7.68 (t, *J* = 7.6, 1.2 Hz, 1H, H_AR_), 7.86 (d, *J* = 7.6, 1.2 Hz, 2H, H_AR_), 12.34 (s, 1H, OH); ^13^C-NMR (100.6 MHz, CDCl_3_): *δ* = 25.8 (C-8), 26.4 (C-7), 27.9 [(*C*H_3_)_3_C], 28.3 (C-4), 32.6 (C-4a), 44.3 (C-3), 51.9 (CH_3_O), 74.1 (C-8a), 83.6 [(CH_3_)_3_*C*], 98.4 (C-5), 128.5 (2CH_AR_), 130.8 (2CH_AR_), 134.3 (CH_AR_), 135.0 (C_AR_), 151.8 (CO), 166.7 (CO), 171.5, 173.9 (CO, C-6); HRMS (ESI) calcd for [C_22_H_27_NO_8_S + NH_4_^+^]: 483.1796, found: 483.1789.

*cis-6-Hydroxy-5-(methoxycarbonyl)-2-methyl-1-oxo-8a-(phenylsulfonyl)-1,2,3,4,4a,7,8,8a-octahydroisoqui- noline* (**18b**). Operating as described in the general procedure, from unsaturated lactam **6b** (60 mg, 0.24 mmol) in anhydrous CH_2_Cl_2_ (35 mL), Nazarov reagent **17** (144 mg, 0.71 mmol), and Cs_2_CO_3_ (469 mg, 1.44 mmol) in anhydrous CH_2_Cl_2_ (15 mL), *cis*-hydroisoquinolone **18b** was obtained (32 mg, 35% yield): IR (ATR Pike) ν (cm^−1^): 1653 (CO); ^1^H-NMR (400 MHz, CDCl_3_, COSY, g-HSQC): *δ* = 1.97 (m, 1H, H-4), 2.00-2.15 (m, 3H, H-7, 2H-8), 2.35 (m, 1H, H-7), 2.46-2.54 (ddt, *J* = 13.8, 8.8, 4.2 Hz, 1H, H-4), 2.93 (s, 3H, CH_3_N), 3.17 (ddt, *J* = 12.8, 9.4, 3.8 Hz, 1H, H-3), 3.27 (m, 1H, H-3), 3.84 (s, 3H, CH_3_O), 4.01 (dd, *J* = 6.4, 3.6 Hz, H-4a), 7.51 (t, *J* = 7.6, Hz, 2H, H_AR_), 7.64 (t, *J* = 7.6 Hz, 1H, H_AR_), 7.88 (d, *J* = 7.6 Hz, 2H, H_AR_), 12.40 (s, 1H, OH); ^13^C-NMR (100.6 MHz, CDCl_3_): *δ* = 26.1 (C-7), 26.2 (C-4), 27.2 (C-8), 32.1 (C-4a), 35.8 (CH_3_N), 46.9 (C-3), 51.8 (CH_3_O), 71.6 (C-8a), 97.7 (C-5), 128.6 (2CH_AR_), 130.1 (2CH_AR_), 133.8 (CH_AR_), 136.7 (C_AR_), 164.5 (CO), 171.8, 172.9 (CO, C-6); HRMS calcd for [C_18_H_21_NO_6_S + H^+^]: 380.1162, found: 380.1158.

*cis-6-Hydroxy-5-(methoxycarbonyl)-1-oxo-8a-(phenylsulfonyl)-2-(p-toluenesulfonyl)-1,2,3,4,4a,7,8,8a-octa- hydroisoquinoline* (**18c**). Operating as described in the general procedure, from unsaturated lactam **6c** (50 mg, 0.12 mmol) in anhydrous CH_2_Cl_2_ (20 mL), Nazarov reagent **17** (73 mg, 0.36 mmol) and Cs_2_CO_3_ (234 mg, 0.72 mmol) in anhydrous CH_2_Cl_2_ (5 mL), *cis*-hydroisoquinolone **18c** was obtained (20 mg, 32% overall yield for the three steps). IR (ATR Pike) ν (cm^−1^): 1653 (CO); ^1^H-NMR (400 MHz, CDCl_3_, COSY, g-HSQC): *δ* = 1.85−1.93 (m, 2H, H-4, H-7), 1.99-2.04 (m, 2H, H-7, H-8), 2.29-2.35 (m, 1H, H-8), 2.50 (s, 3H, CH_3_), 2.75-2.70 (m, 1H, H-4), 3.84 (s, 3H, CH_3_O) 3.91 (m, 1H, H-3), 4.01 (m, 1H, H-3), 7.22 -7.34 (m, 6H, H_AR_), 7.60 (td, *J* = 7.2, 1.2 Hz, 1H, H_AR_), 7.91 (d, *J* = 8.8 Hz, 2H, H_AR_), 12.34 (s, 1H, OH); ^13^C-NMR (100.6 MHz, CDCl_3_): *δ* = 21.7 (CH_3_), 25.7 (C-8), 27.0 (C-7), 28.3 (C-4), 31.9 (C-4a), 43.8 (C-3), 52.0 (CH_3_O), 73.1 (C-8a), 97.8 (C-5), 128.3-130.7 (CH_AR_), 134.3 (CH_AR_), 134.6, 135.2 (C_AR_, CH_AR_), 145.1 (C_AR_), 165.5 (CO), 171.4, 172.9 (CO, C-6); HRMS (ESI) calcd for [C_24_H_25_NO_8_S_2_ + H^+^]: 520.1094, found: 520.1108.

*cis-2-(tert-Butoxycarbonyl)-8a-(ethoxycarbonyl)-6-hydroxy-5-(methoxycarbonyl)-1-oxo-1,2,3,4,4a,7,8,8a- octahydroisoquinoline* (**19**). Operating as described in the general procedure, from unsaturated lactam **8** (90 mg, 0.33 mmol) in anhydrous CH_2_Cl_2_ (60 mL), Nazarov reagent **17** (202 mg, 1 mmol), and Cs_2_CO_3_ (652 mg, 2 mmol) in anhydrous CH_2_Cl_2_ (5 mL), *cis*-hydroisoquinolone **19** was obtained (87 mg, 60% yield): IR (ATR Pike) ν (cm^−1^): 1725 (CO), 1654 (CO); ^1^H-NMR (400 MHz, CDCl_3_, COSY, g-HSQC): *δ* = 1.09 (d, *J* = 7.2 Hz, 3H, CH_2_C*H*_3_), 1.36 [s, 9H, CH_3_)_3_C], 1.51 (ddd, *J* = 14.0, 12.0, 4.8 Hz, 1H, H-4), 1.90 (ddd, *J* = 14.0, 11.2, 7.2 Hz, 1H, H-8), 2.03 (dq, *J* = 14.0, 3.6 Hz, 1H, H-4), 2.18 (ddd, *J* = 18.4, 7.2, 2.0 Hz, 1H, H-7), 2.32 (dd, *J* = 14.0, 7.2 Hz, 1H, H-8), 2.63 (ddd, *J* = 18.4, 10.4, 8.0 Hz, 1H, H-7), 3.31 (dd, *J* = 11.6, 3.2 Hz, 1H, H-4a), 3.40 (td, *J* = 12.4, 3.6 Hz, 1H, H-3), 3.64 (s, 3H, CH_3_O), 3.66 (ddd, *J* = 12.4, 4.8, 3.2 Hz, 1H, H-3), 4.05 (qd, *J* = 7.2, 2.4 Hz, 2H, C*H*_2_CH_3_), 12.18 (s, 1H, OH); ^13^C-NMR (100.6 MHz, CDCl_3_): *δ* = 13.9 (CH_2_*C*H_3_), 24.6 (C-8), 26.3 (C-7), 27.5 (C-4), 27.9 [(*C*H_3_)_3_C], 34.7 (C-4a), 45.7 (C-3), 51.7 (CH_3_O), 56.1 (C-8a), 61.7 (*C*H_2_CH_3_), 83.4 [(CH_3_)_3_*C*], 98.0 (C-5), 152.7 (C-6), 170.4 (CO), 170.5 (CO), 171.9 (CO), 172.7 (CO); HRMS (ESI) calcd for [C_19_H_27_NO_8_ + Na^+^]: 420.1629, found: 420.1645.

### 3.4. General Procedure for the Desulfonylation Reactions

Na_2_HPO_4_ (50 equiv.) and sodium amalgam (25 equiv.) were added at −78 ˚C under an argon atmosphere to a solution of the sulfonyl derivative **10a**, **12a** or **12b** (1 equiv.) in anhydrous methanol (0.025 M), and the mixture was stirred at −78 ˚C for 2 h. The solution was then filtered and quenched with H_2_O at low temperature. The mixture was concentrated under reduced pressure, and the resulting aqueous solution was extracted with EtOAc. The combined organic extracts were dried over anhydrous MgSO_4_, filtered, and concentrated under reduced pressure. Flash chromatography (9:1 hexane-EtOAc) of the residue afforded the desulfurated compounds **13a**, **14a** or **14b** (detailed data in supplementary materials). 

*cis-2-(tert-Butoxycarbonyl)-6-hydroxy-5-(methoxycarbonyl)-8-methyl-1-oxo-1,2,3,4,4a,7,8,8a-octahydroiso- quinoline* (*H-8/H-8a trans*, **13a**). Operating as described in the above general desulfonylation procedure, from a solution of sulfonyl derivative **10a** (580 mg, 1.21 mmol) in anhydrous methanol (50 mL), Na_2_HPO_4_ (8.60 g, 60.6 mmol), and sodium amalgam (6.77 g, 30.3 mmol), compound **13a** was obtained (357 mg, 87% yield): ^1^H-NMR (400 MHz, CDCl_3_, COSY, g-HSQC): *δ* = 1.05 (d, *J* = 6.0 Hz, 3H, CH_3_), 1.53 [s, 9H, (CH_3_)_3_C], 1.79 (dddd, *J* = 19.2, 12.8, 11.2, 4.8 Hz, 1H, H-4), 2.04 (m, 1H, H-7), 2.10 (m, 1H, H-4), 2.30 (m, 2H, H-8, H-8a), 2,46 (dd, *J* = 19.2, 4.8 Hz, 1H, H-7), 2.98 (dt, *J* = 11.2, 4.0 Hz, 1H, H-4a), 3.41 (td, *J* = 12.8, 4.0 Hz, 1H, H-3), 3.70-3.75 (m, 1H, H-3), 3.79 (s, 3H, CH_3_O), 12.23 (s, 1H, OH); ^13^C-NMR (100.6 MHz, CDCl_3_): *δ* = 18.8 (CH_3_), 26.9 (C-8), 27.1 (C-4), 27.9 [(*C*H_3_)_3_C], 32.0 (C-4a), 36.8 (C-7), 45.2 (C-3), 50.5 (C-8a), 51.6 (CH_3_O), 82.7 [(CH_3_)_3_*C*], 99.3 (C-5), 153.3 (CO), 169.0 (CO), 172.2, 173.1 (CO, C-6); HRMS (ESI) calcd for [C_17_H_25_NO_6_ + Na^+^]: 362.1574, found: 362.1579.

*cis-6-Hydroxy-5-(methoxycarbonyl)-2-methyl-1-oxo-1,2,3,4,4a,7,8,8a-octahydroisoquinoline* (*H-8/H-8a trans*, **14a**). Operating as described in the general desulfonylation procedure, from a solution of sulfone **12a** (100 mg, 0.25 mmol) in anhydrous MeOH (10 mL), Na_2_HPO_4_ (1.81 g, 12.7 mmol), and sodium amalgam (1.42 g, 6.36 mmol), compound **14a** was obtained (48 mg, 76% yield) as a white foam: IR (ATR Pike) ν (cm^−1^): 1645 (CO); ^1^H-NMR (400 MHz, CDCl_3_, COSY, g-HSQC): *δ* = 1.09 (d, *J* = 7.2 Hz, 3H, CH_3_), 1.90 (m, 1H, H-4), 1.94–2.10 (m, 2H, H-4, H-7), 2.24 (m, 2H, H-8, H-8a), 2.38 (d, *J* = 18.4, 4.4 Hz, 1H, H-7), 2.90 (m, 1H, H-4a), 2.92 (s, 3H, CH_3_N), 3.21–3.25 (m, 2H, H-3), 3.79 (s, 3H, CH_3_O), 12.30 (s, 1H, OH); ^13^C-NMR (100.6 MHz, CDCl_3_): *δ* = 19.2 (CH_3_), 26.5 (C-4), 27.4 (C-8a), 31.3 (C-4a), 34.8 (CH_3_N), 37.1 (C-7), 47.2 (C-8), 48.8 (C-3), 51.5 (CH_3_O), 99.1 (C-5), 170.7 (CO), 172.3 (C-6), 172.6 (CO); HRMS (ESI) calcd for [C_13_H_20_NO_4_ + H^+^]: 254.1387, found: 254.1387.

*cis-6-Hydroxy-5-(methoxycarbonyl)-2-methyl-1-oxo-1,2,3,4,4a,7,8,8a-octahydroisoquinoline* (*H-8/H-8a cis*, **14b**). Operating as described in the general desulfonylation procedure, from a solution of sulfone **12b** (340 mg, 0.87 mmol) in anhydrous MeOH (10 mL), Na_2_HPO_4_ (6.134 g, 43.2 mmol), and sodium amalgam (4.836 g, 21.6 mmol), compound **14b** was obtained (137 mg, 62% yield) as a white foam: ^1^H-NMR (400 MHz, CDCl_3_, COSY, g-HSQC): *δ* = 1.05 (d, *J* = 7.2 Hz, 3H, CH_3_), 1.86 (qd, *J* = 12.0, 5.2, 1H, H-4), 2.10 (dm, *J* = 12.0 Hz, 1H, H-4), 2.19 (dd, *J* = 18.0, 2.0 Hz, 1H, H-7), 2.657-2.64 (m, 12H, H-8, H-7), 2.67 (m, 1H, H-8a), 2.85–2.90 (m, 1H, H-4a), 3.00 (s, 3H, NCH_3_), 3.24 (dq, *J* = 12.0, 3.2 Hz, 1H, H-3), 3.39 (td, *J* = 12.0, 4.4 Hz, 1H, H-3), 3.79 (s, 3H, CH_3_O), 12.48 (s, 1H, OH); ^13^C-NMR (100.6 MHz, CDCl_3_): *δ* = 18.8 (CH_3_), 26.6 (C-4), 29.4 (C-8a), 31.9 (C-4a), 34.9 (CH_3_N), 36.1 (C-7), 43.6 (C-8), 49.2 (C-3), 51.5 (CH_3_O), 98.0 (C-5), 171.2 (CO), 172.6 (C-6, CO).

### 3.5. General Procedure for the Removal of the N-Boc Substituent

TFA (1:3 TFA/CH_2_Cl_2_ ratio) was added to a solution of the *N*-Boc derivative **10a** or **13a** in anhydrous CH_2_Cl_2_ (0.05-0.06 M), and the mixture was stirred for 30 minutes at room temperature. Toluene (1 mL) was added to the resulting solution, and the mixture was concentrated under reduced pressure. After a second addition of toluene (1 mL) and concentration of the mixture, flash chromatography (7:3 to 3:7 hexane-EtOAc) of the residue afforded the deprotected compound **15** or **16**.

*cis-6-Hydroxy-5-(methoxycarbonyl)-8-methyl-1-oxo-8a-(phenylsulfonyl)-1,2,3,4,4a,7,8,8a-octahydroisoqui- noline* (*Me/SO_2_Ph cis*, **15**). Operating as described in the above general procedure, from a solution of the *N*-Boc derivative **10a** (23 mg, 0.048 mmol) in anhydrous CH_2_Cl_2_ (750 μL) and TFA (250 μL), compound **15** was obtained (18 mg, 98% yield): IR (ATR Pike) ν (cm^−1^): 3352 (NH), 1668 (CO); ^1^H-NMR (400 MHz, CDCl_3_, COSY, g-HSQC): *δ* = 1.11 (d, *J* = 6.8 Hz, 3H, CH_3_), 1.94 (dd, *J* = 17.6, 2.0 Hz, H-7), 2.32 (dm, *J* = 14.8 Hz, 1H, H-4), 2.45 (m, 1H, H-8), 2.50 (dm, *J* = 17.6 Hz, 1H, H-7), 2.65 (dddd, *J* = 14.8, 12.4, 5.6, 3.6 Hz, 1H, H-4), 3.16 (td, *J* = 12.4, 4.4 Hz, 1H, H-3), 3.27 (m, 1H, H-3), 3.85 (s, 3H, CH_3_O), 3.97 (br.s, 1H, H-4a), 6.29 (br.s, 1H, NH), 7.53 (t, *J* = 7.2, Hz, 2H, H_AR_), 7.65 (t, *J* = 7.2 Hz, 1H, H_AR_), 8.06 (d, *J* = 7.2 Hz, 2H, H_AR_), 12.33 (s, 1H, OH); ^13^C-NMR (100.6 MHz, CDCl_3_): *δ* = 13.7 (CH_3_), 22.4 (C-4), 29.2 (C-4a), 30.1 (C-8), 35.2 (C-7), 38.7 (C-3), 51.8 (CH_3_O), 75.1 (C-8a), 96.5 (C-5), 128.3 (2CH_AR_), 130.6 (2CH_AR_), 134.0 (CH_AR_), 137.7 (C_AR_), 167.0 (CO), 171.6, 171.8 (CO, C-6); HRMS calcd for [C_18_H_21_NO_6_S + H^+^]: 380.1162, found: 380.1164.

*cis-6-Hydroxy-5-(methoxycarbonyl)-8-methyl-1-oxo-1,2,3,4,4a,7,8,8a-octahydroisoquinoline* (*H-8/H-8a trans*, **16**). Operating as described in the above general procedure, from a solution of the *N*-Boc derivative **13a** (10 mg, 0.03 mmol) in anhydrous CH_2_Cl_2_ (600 μL) and TFA (200 μL), compound **16** was obtained (7 mg, 99% yield): IR (ATR Pike) ν (cm^−1^): 3340 (NH), 1659 (CO); ^1^H-NMR (400 MHz, CDCl_3_, COSY, g-HSQC): *δ* = 1.12 (d, *J* = 6.8 Hz, 3H, CH_3_), 1.83 (m, 1H, H-4), 1.98 (dd, *J* = 13.6, 3.6 Hz, 1H, H-4), 2.06 (dd, *J* = 18.4, 8.8 Hz, H-7), 2.23 (m, 2H, H-8, H-8a), 2.42 (dd, *J* = 18.4, 4.8 Hz, 1H, H-7), 2.93 (m, 1H, H-4a), 3.23-3.40 (m, 2H, H-3), 3.79 (s, 3H, CH_3_O), 6.22 (br.s, 1H, NH), 12.29 (s, 1H, OH); ^13^C-NMR (100.6 MHz, CDCl_3_): *δ* = 19.1 (CH_3_), 25.7 (C-4), 27.3 (C-8, C-8a), 31.0 (C-4a), 37.1 (C-7), 41.1 (C-3), 51.6 (CH_3_O), 99.1 (C-5), 172.2, 172.4 (CO, C-6); HRMS calcd for [C_12_H_17_NO_4_ + H^+^]: 240.1230, found: 240.1232.

## Figures and Tables

**Table 1:**
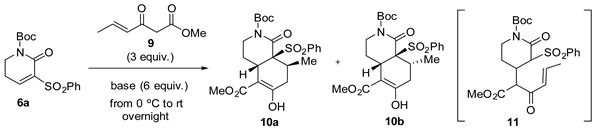
Reaction of unsaturated lactam **6a** with Nazarov reagent **9**.

Entry	Base	Solvent	Concentration (mM)	Yield (%)	a/b Ratio
1	Cs_2_CO_3_	CH_2_Cl_2_	50	47	3:1
2	Cs_2_CO_3_	CH_2_Cl_2_	25	56	3:1
3	Cs_2_CO_3_	CH_2_Cl_2_	5	79	3:1
4	Cs_2_CO_3_	MeCN	5	10	–
5	KF	MeOH	5	56	1:2

**Table 2:**
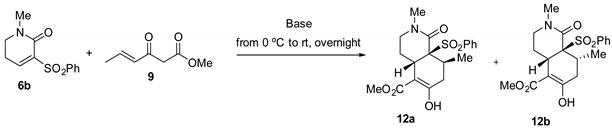
Reaction of unsaturated lactam **6b** with Nazarov reagent **9**.

Entry	Base	Equiv Base	Equiv (9)	Solvent	Concentration (mM)	Yield (%)	a/b Ratio
1	Cs_2_CO_3_	6	3	CH_2_Cl_2_	100	28	4:1
2	Cs_2_CO_3_	6	3	CH_2_Cl_2_	5	40	4:1
3	KF	6	3	MeOH	5	51	1:4
4	Cs_2_CO_3_	4	2	DMF	5	45	1:5

**Table 3:**
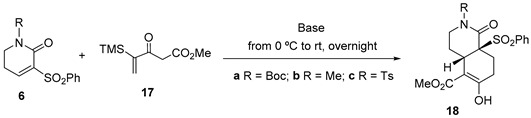
Reactions of unsaturated lactams **6a-c** with Nazarov reagent **17**.

Entry	Unsaturated Lactam	Base	Equiv Base	Equiv (15)	Solvent	Concentration (mM)	Product	Yield (%)
1	**6a**	Cs_2_CO_3_	6	3	CH_2_Cl_2_	5	**18a**	47
2	**6a**	Cs_2_CO_3_	10	5	CH_2_Cl_2_	5	**18a**	47
3	**6a**	Cs_2_CO_3_	6	3	CH_2_Cl_2_	20	**18a**	20
4	**6a**	KF	6	3	MeOH	5	**18a**	20
5	**6b**	Cs_2_CO_3_	6	3	CH_2_Cl_2_	5	**18b**	35
6	**6b**	Cs_2_CO_3_	6	3	CH_2_Cl_2_	100	**18b**	21
7	**6b**	KF	6	3	MeOH	5	**18b**	20
8	**6c**	Cs_2_CO_3_	6	3	CH_2_Cl_2_	5	**18c**	32

## Supplementary Materials

The following are available online. Copies of ^1^H, ^13^C, and NOE NMR spectra, and crystallographic data for **10a** (CCDC 1890851), **12a** (CCDC 1890850), and **12b** (CCDC 1890852). CCDC 1890850-1890852 contains the supplementary crystallographic data for this paper. These data can be obtained free of charge via http://www.ccdc.cam.ac.uk/conts/retrieving.html (or from the CCDC, 12 Union Road, Cambridge CB2 1EZ, UK; Fax: +44 1223 336033; E-mail: deposit@ccdc.cam.ac.uk).
