# Supplementary material for: A Straightforward Synthesis of Functionalized cis-Perhydroisoquinolin-1-ones
Source: Molecules. 2019 Feb 3;24(3):557. doi: 10.3390/molecules24030557 (PMC6385050; doi:10.3390/molecules24030557)
Supplement: Supplementary file 1 [file molecules-24-00557-s001.pdf]

Supporting Information

A Straightforward Synthesis of Functionalized *cis*-Perhydroisoquinolin-1-ones

Federica Arioli [1], Maria Pérez [2], Celeste Are [1], Elies Molins [3], Joan Bosch [1] and Mercedes Amat [1,*]

[1] Laboratory of Organic Chemistry, Faculty of Pharmacy and Food Sciences, and Institute of Biomedicine (IBUB), University of Barcelona, 08028 Barcelona, Spain; fede.arioli@hotmail.it (F.A.); cele.are@ub.edu (C.A.); joanbosch@ub.edu (J.B.)

[2] Department of Nutrition, Food Sciences and Gastronomy, Faculty of Pharmacy and Food Sciences, and Institute of Nutrition and Food Safety (INSA-UB), University of Barcelona, 08921 Santa Coloma de Gramanet, Spain; mariaperez@ub.edu

[3] Institut de Ciència de Materials de Barcelona (ICMAB-CSIC), Campus UAB, 08193 Cerdanyola, Spain; elies.molins@icmab.es

* Correspondence: amat@ub.edu; Tel.: +34-93-402-4540

Contents

I) Copies of ^{1}H- and ^{13}C-NMR spectra S2–S26

II) Copies of NOE NMR spectra of compounds **13a**, **14a**, and **14b** S27–S29

III) X- ray crystallographic data for compounds **10a**, **12a**, and **12b** S30–S49

I) Copies of ^{1}H- and ^{13}C-NMR spectra

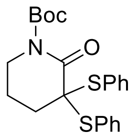

3a

(400 MHz, $CDCl_3$)

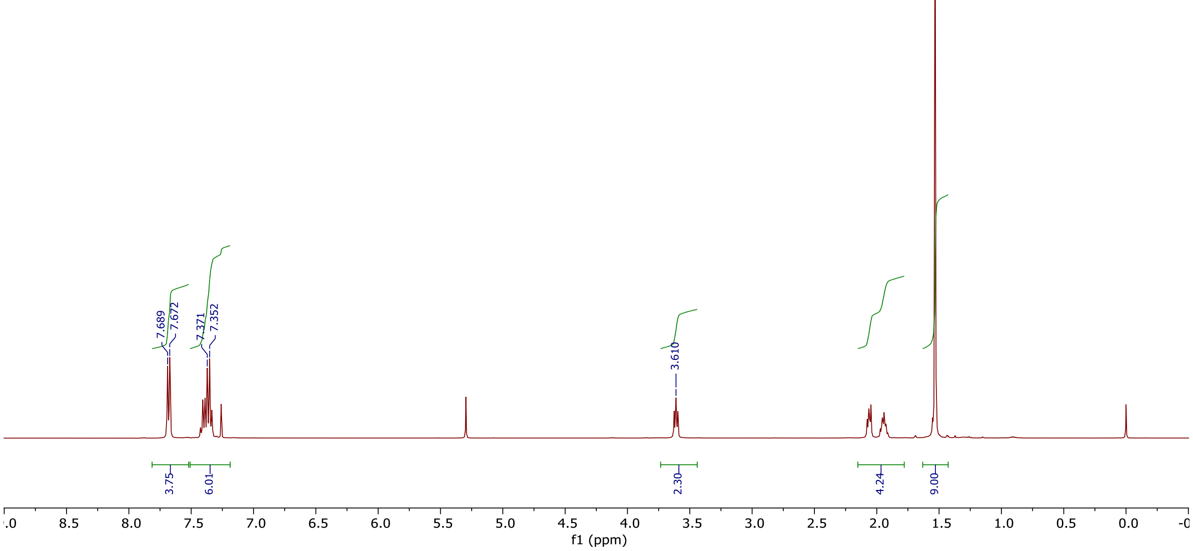

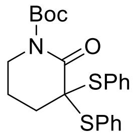

3a

(100.6 MHz, $CDCl_3$)

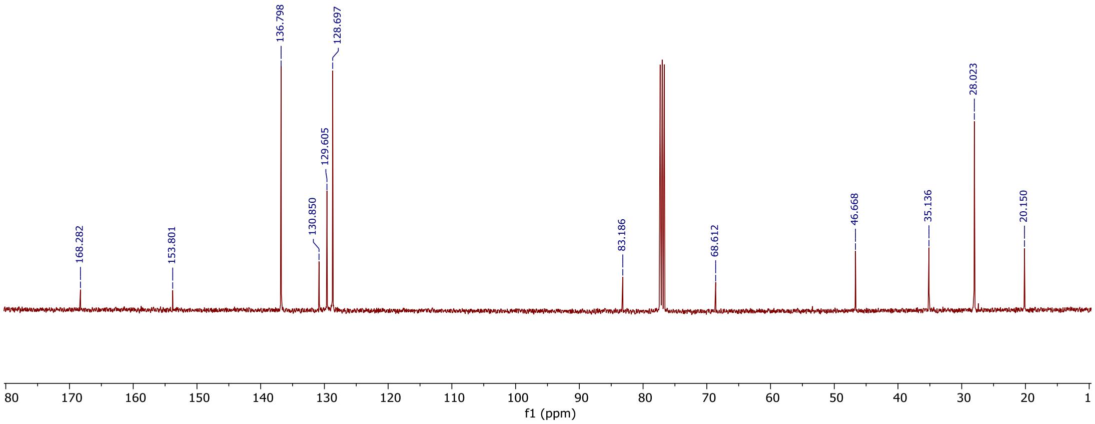

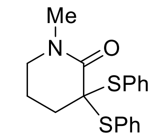

3b
(400 MHz, $CDCl_3$)

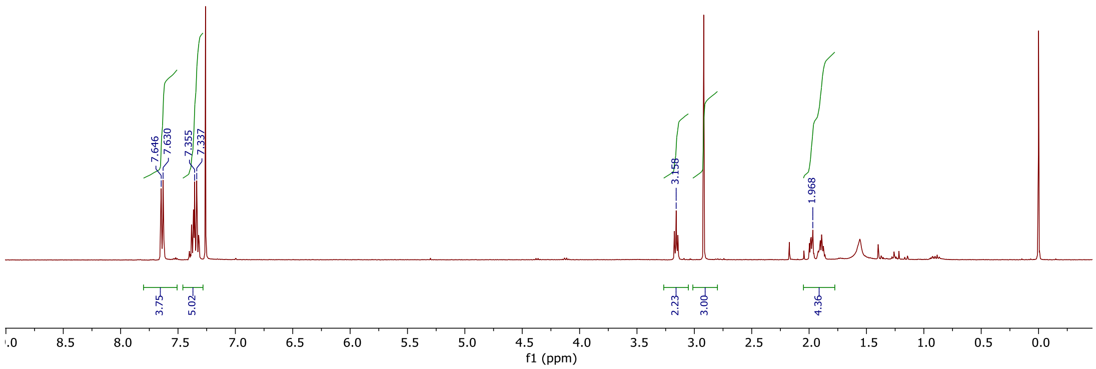

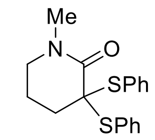

3b
(100.6 MHz, $CDCl_3$)

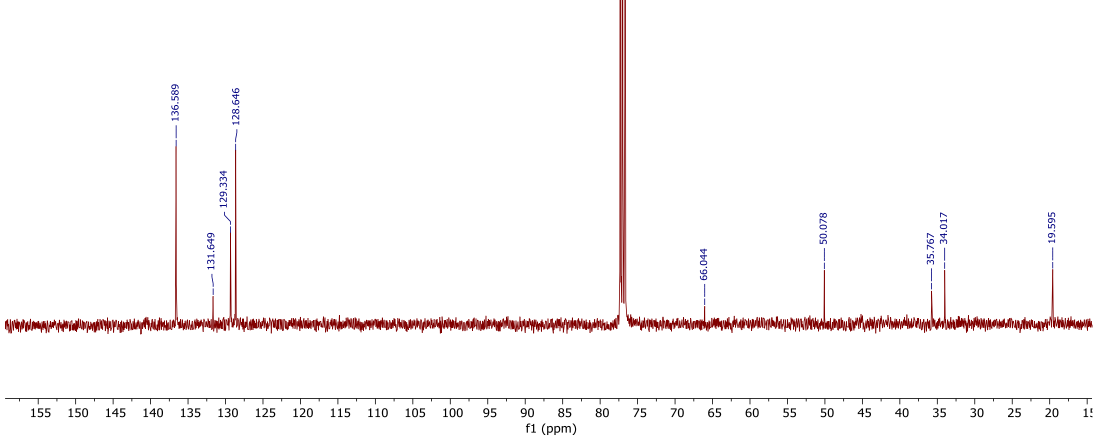

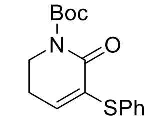

4a
(300 MHz, $CDCl_3$)

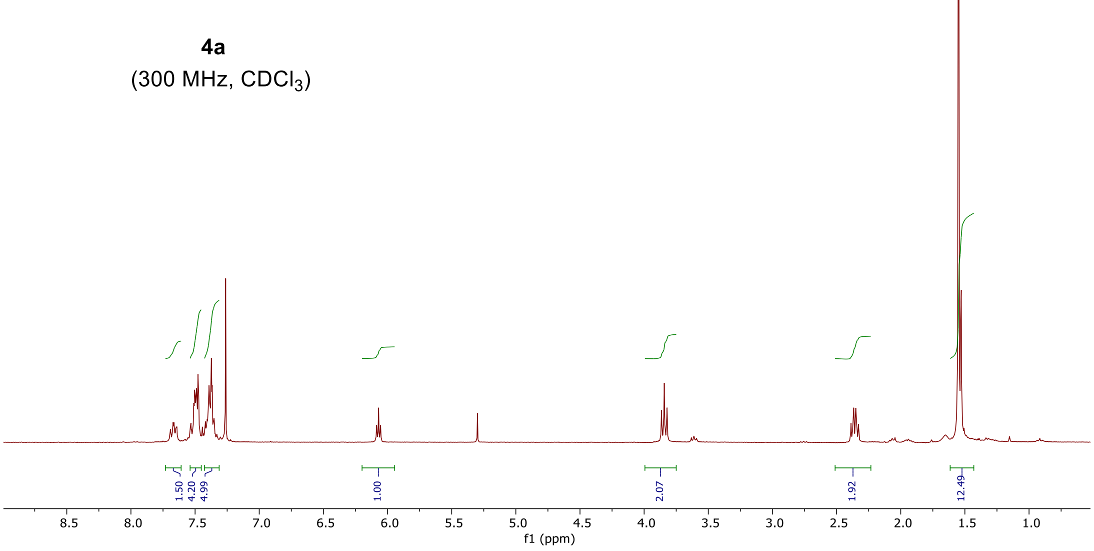

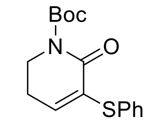

4a
(100.6 MHz, $CDCl_3$)

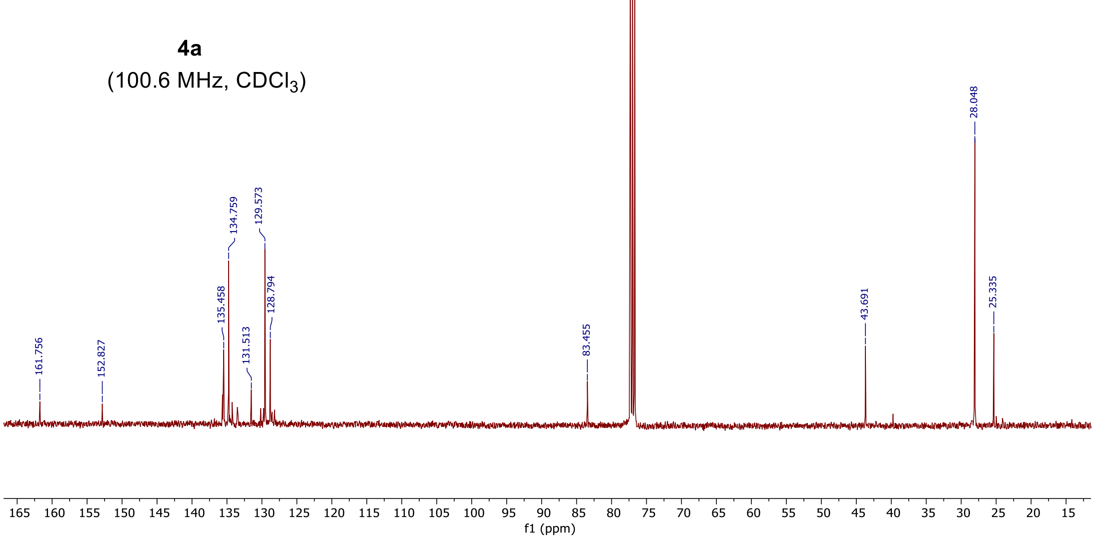

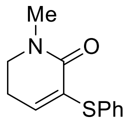

4b

(400 MHz, $CDCl_3$)

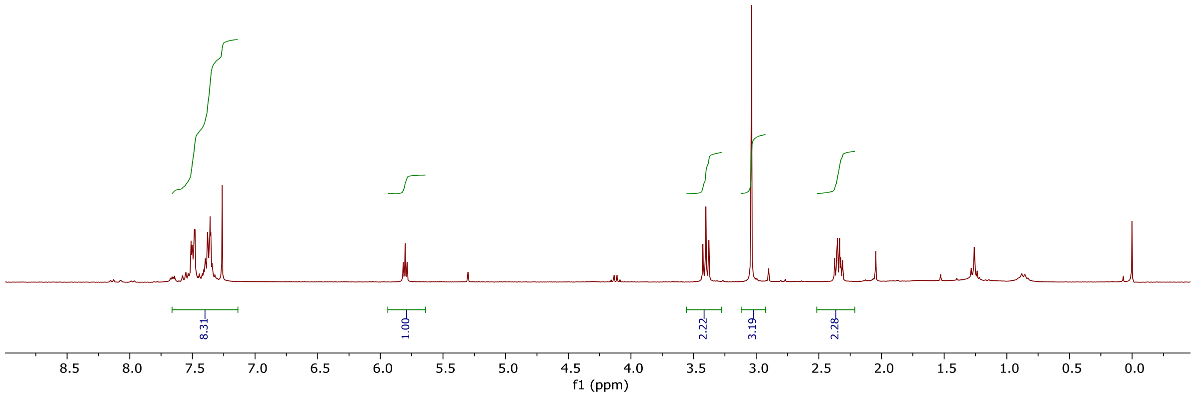

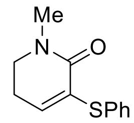

4b

(100.6 MHz, $CDCl_3$)

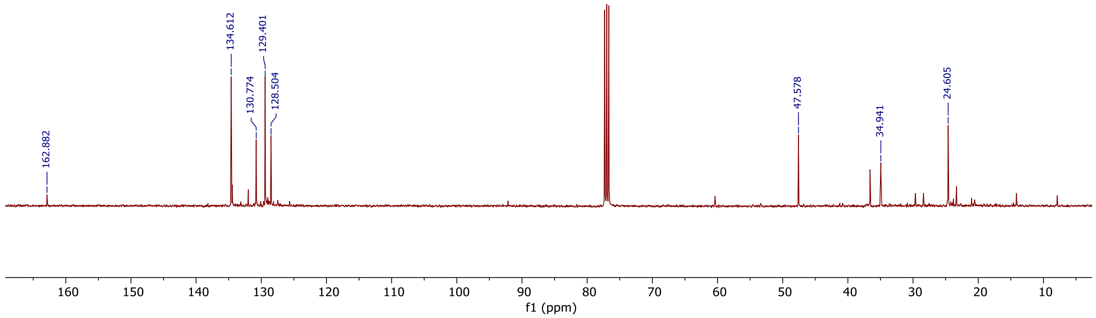

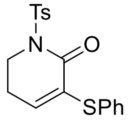

4c
(400 MHz, $CDCl_3$)

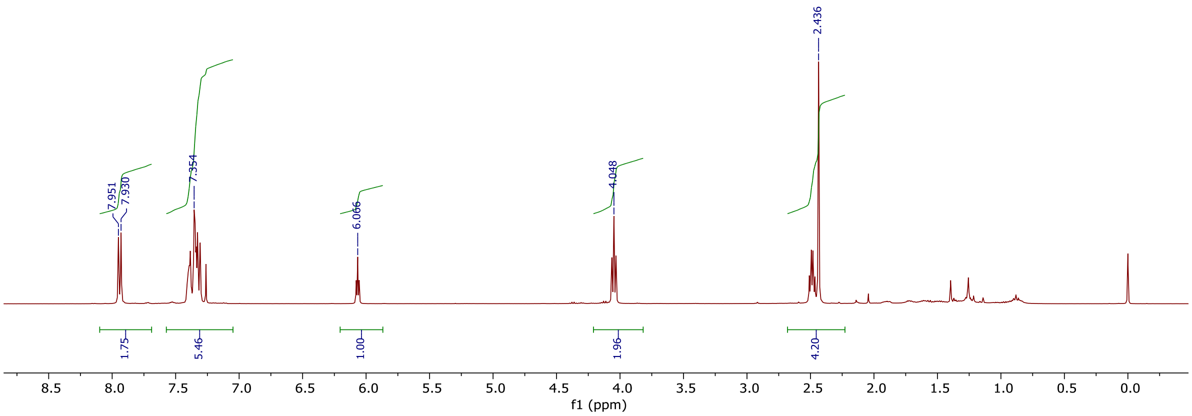

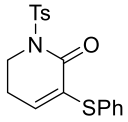

4c
(100.6 MHz, $CDCl_3$)

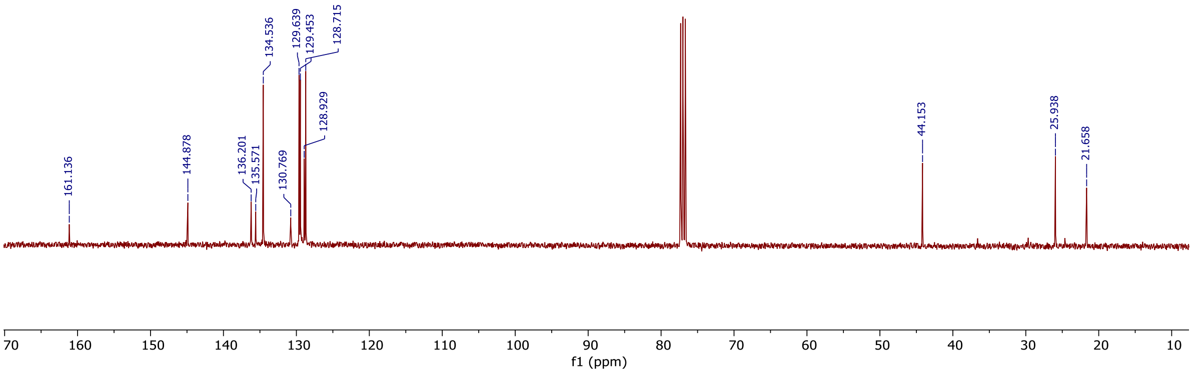

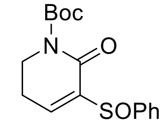

5a
(400 MHz, $CDCl_3$)

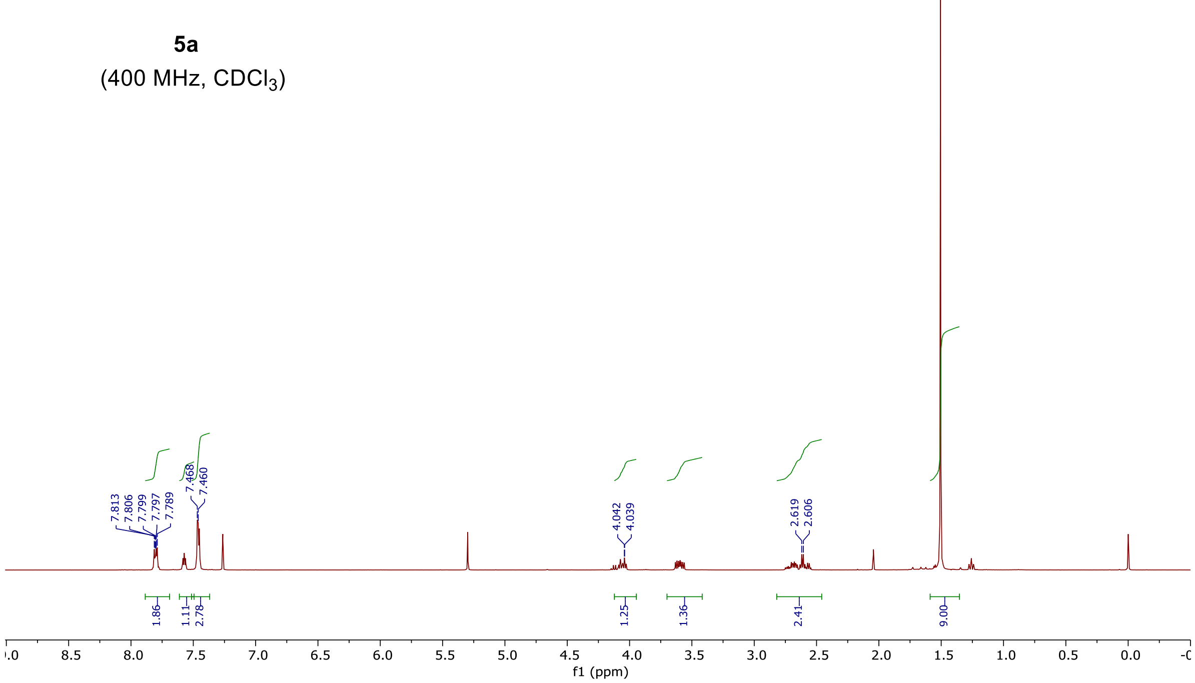

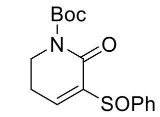

5a
(100.6 MHz, $CDCl_3$)

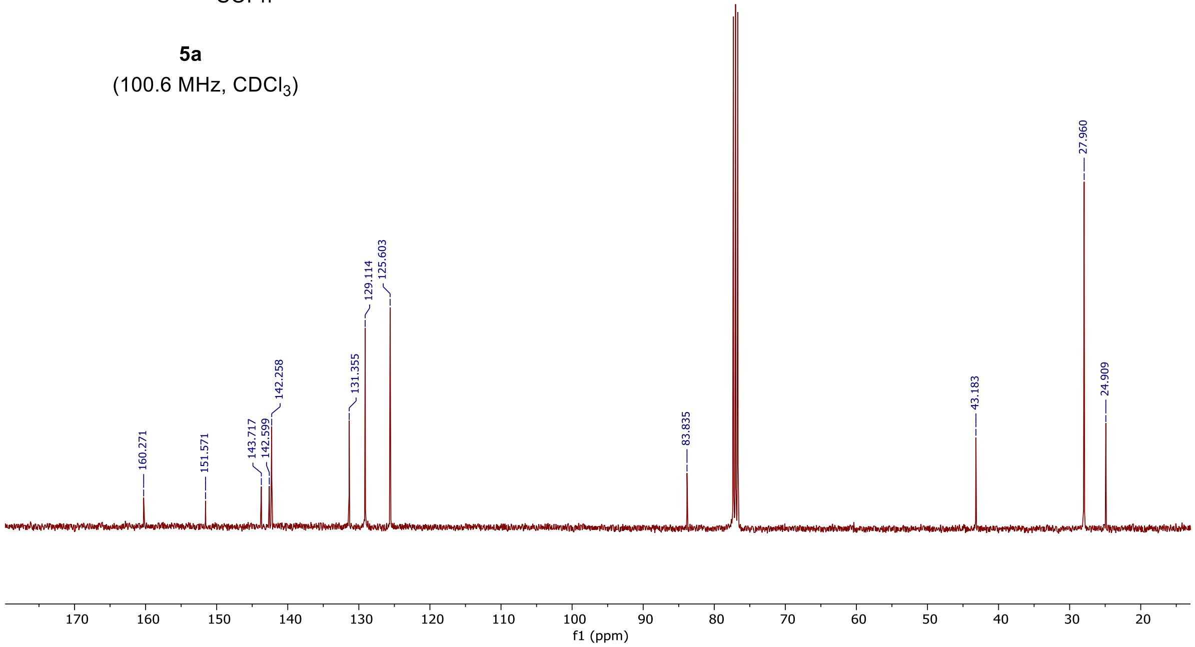

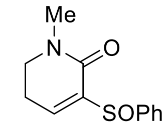

5b
(400 MHz, $CDCl_3$)

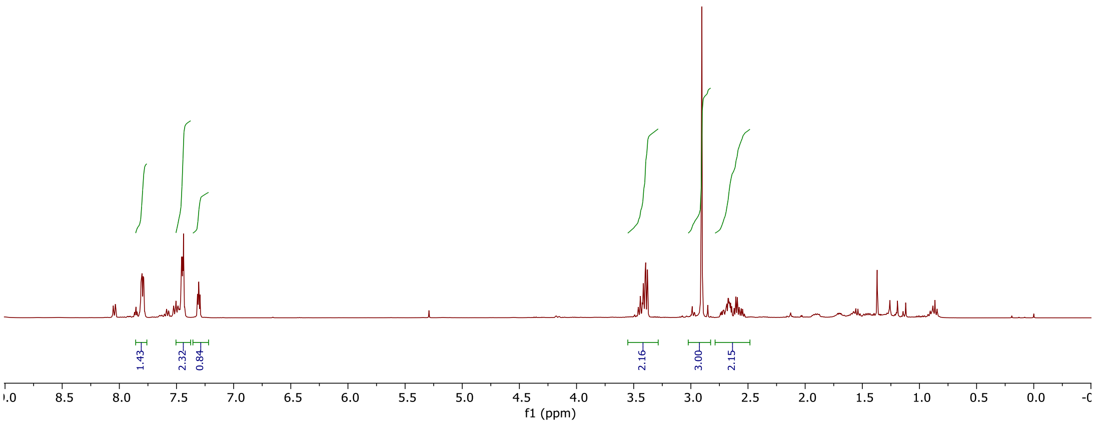

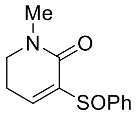

5b
(100.6 MHz, $CDCl_3$)

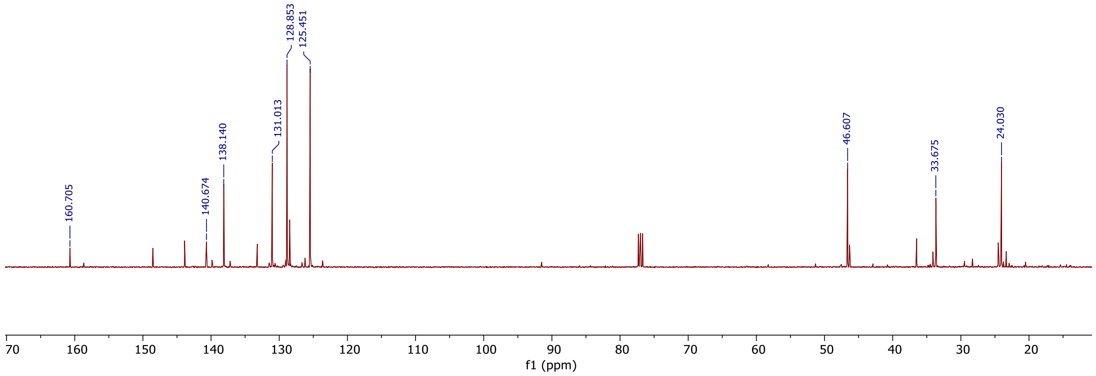

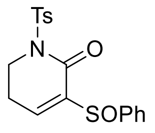

5c
(400 MHz, $CDCl_3$)

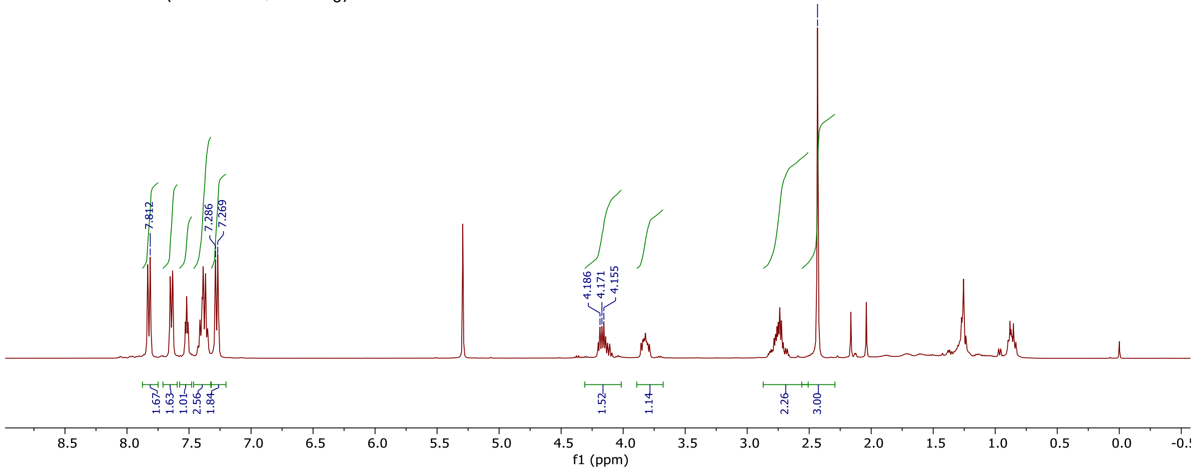

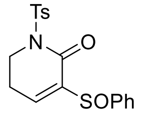

5c
(100.6 MHz, $CDCl_3$)

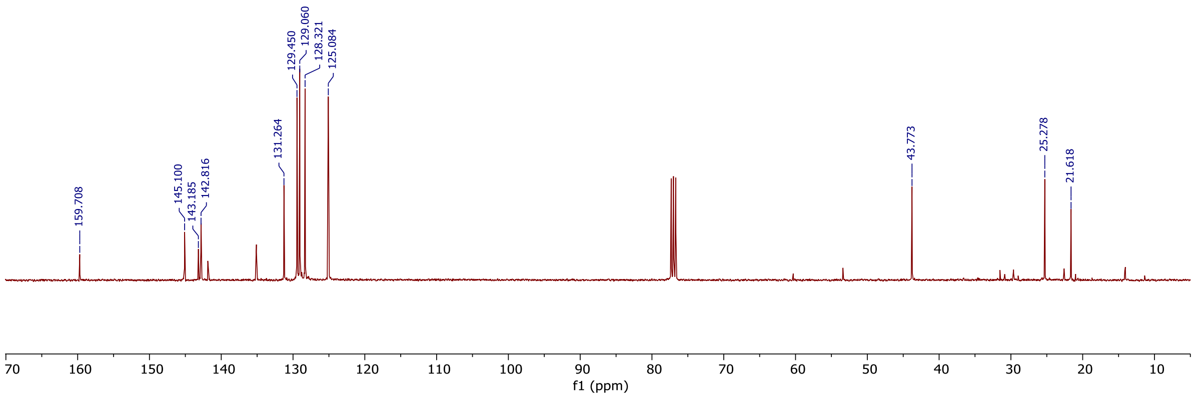

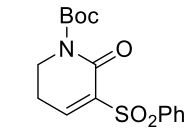

6a

(400 MHz, $CDCl_3$)

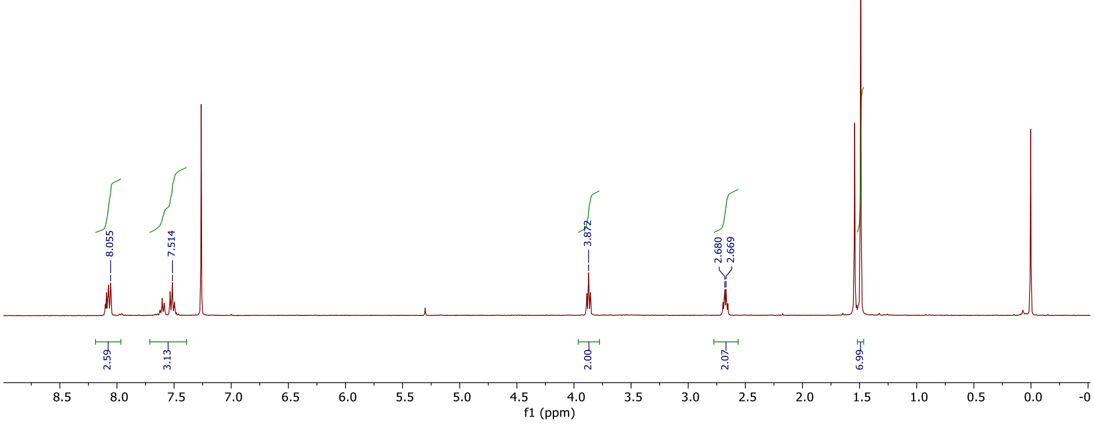

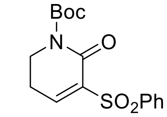

6a

(100.6 MHz, $CDCl_3$)

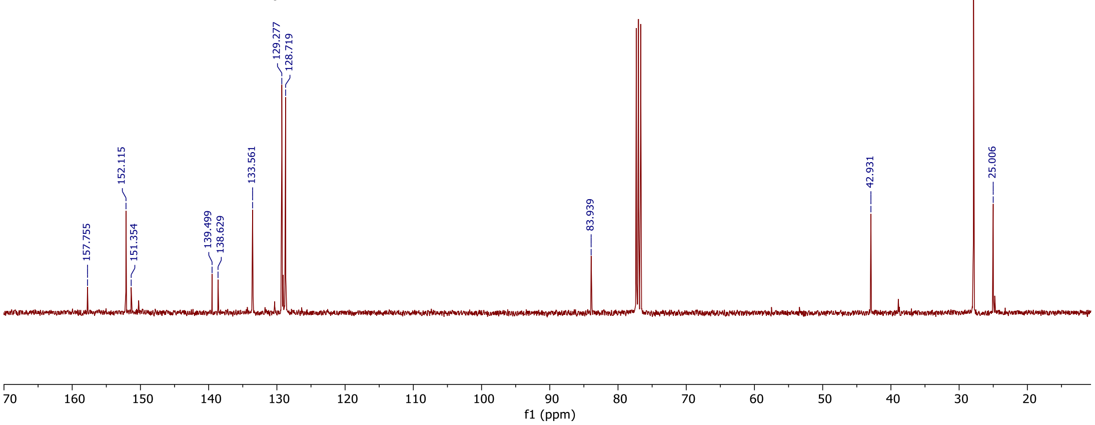

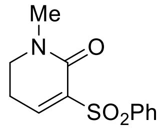

6b
(400 MHz, $CDCl_3$)

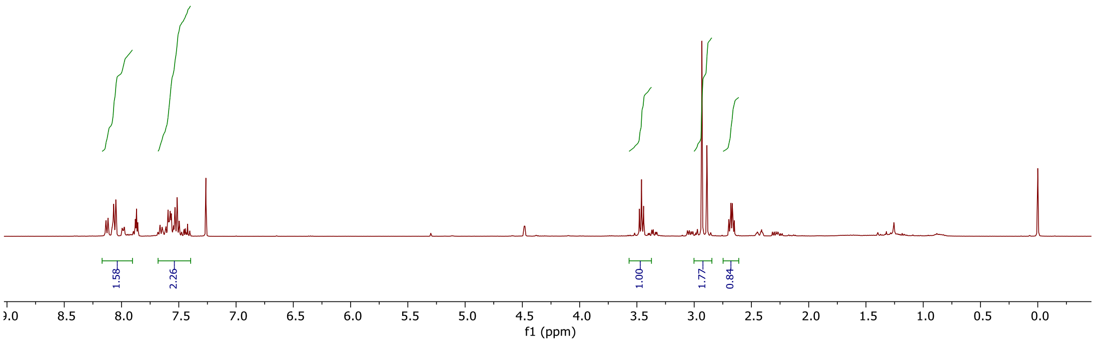

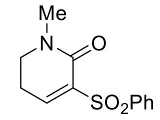

6b
(100.6 MHz, $CDCl_3$)

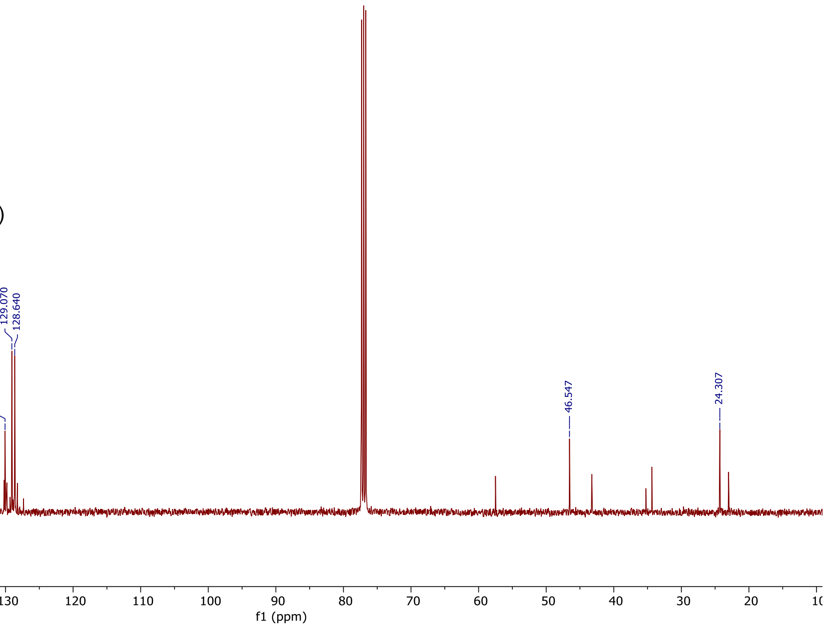

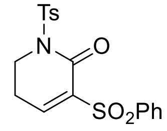

6c
(400 MHz, $CDCl_3$)

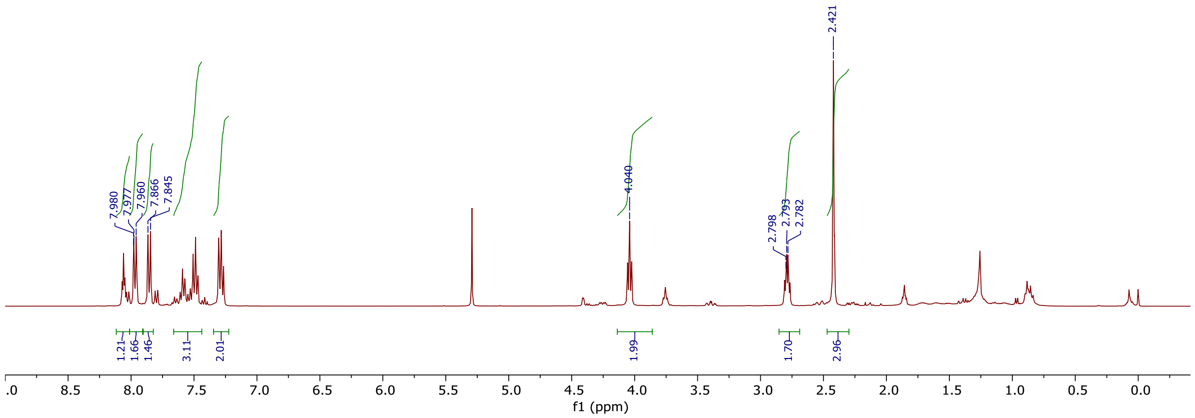

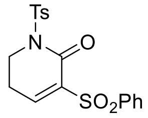

6c
(100.6 MHz, $CDCl_3$)

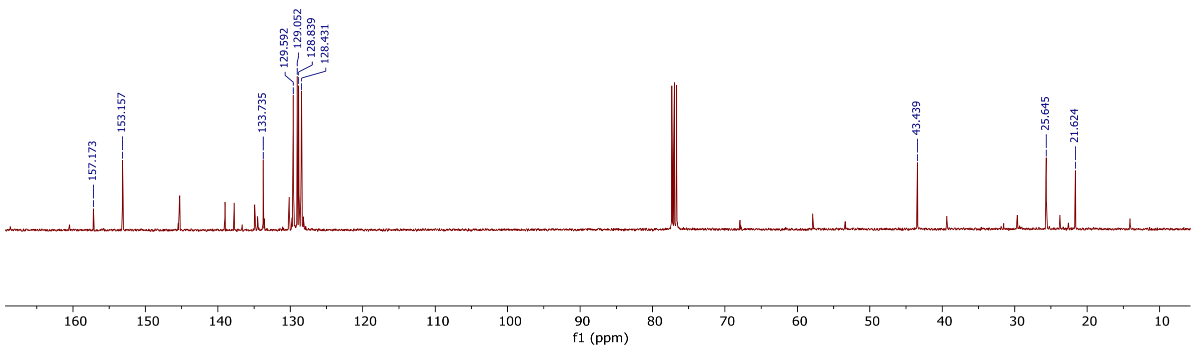

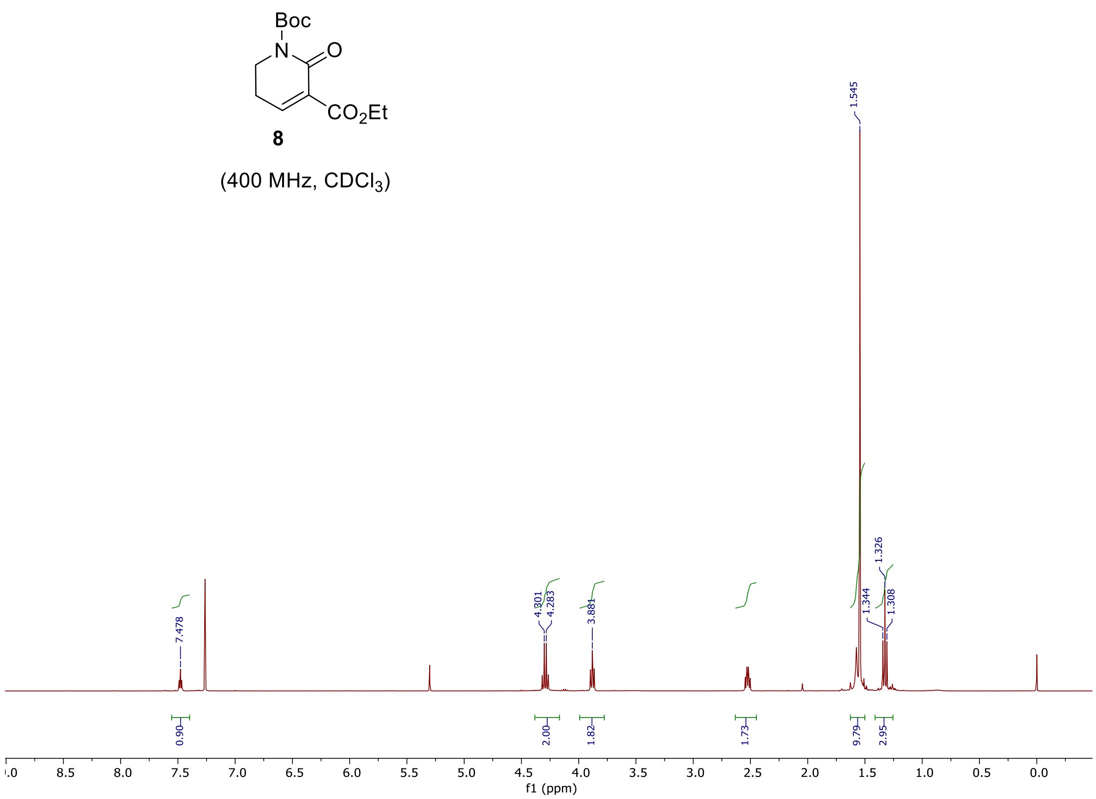
Boc
N
O
CO2Et
8
(400 MHz, CDCl3)
1.545
7.478
4.301
4.283
3.881
1.344
1.326
1.308
0.90
2.00
1.82
1.73
9.79
2.95
8.5 8.0 7.5 7.0 6.5 6.0 5.5 5.0 4.5 4.0 3.5 3.0 2.5 2.0 1.5 1.0 0.5 0.0
f1 (ppm)

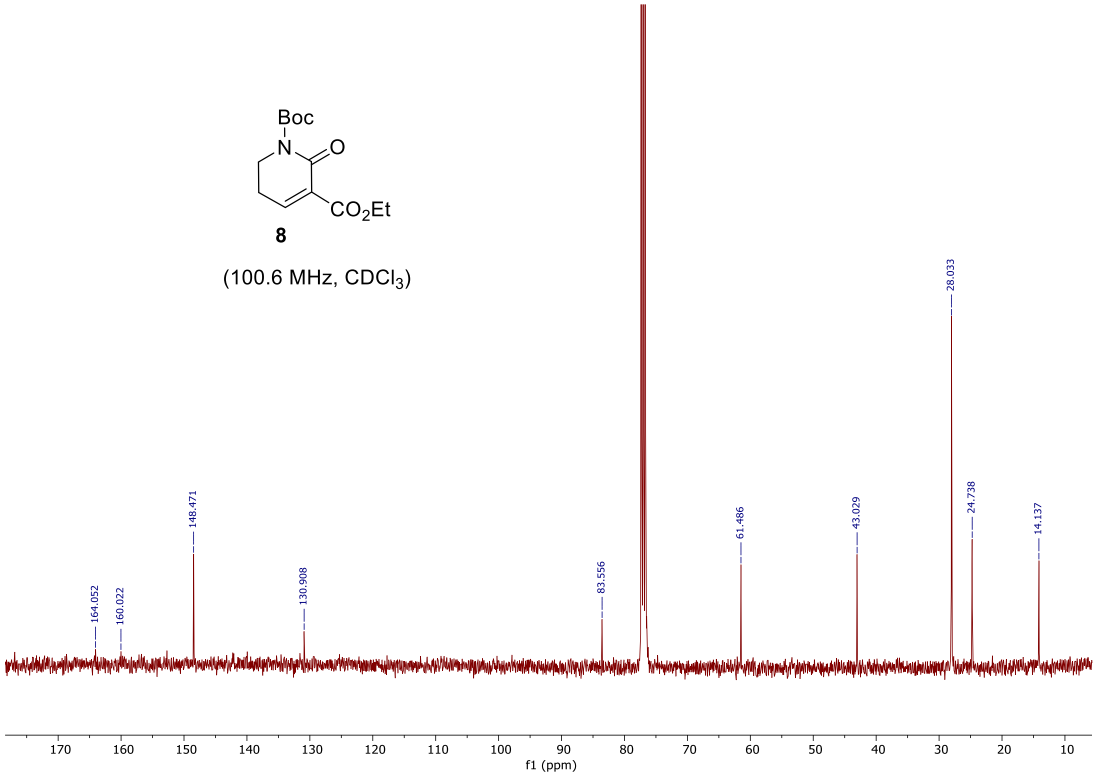
Boc
N
O
CO2Et
8
(100.6 MHz, CDCl3)
164.052
160.022
148.471
130.908
83.556
61.486
43.029
28.033
24.738
14.137
170 160 150 140 130 120 110 100 90 80 70 60 50 40 30 20 10
f1 (ppm)

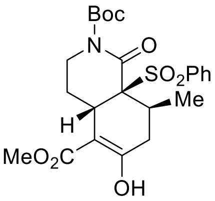

10a

(400 MHz, $CDCl_3$)

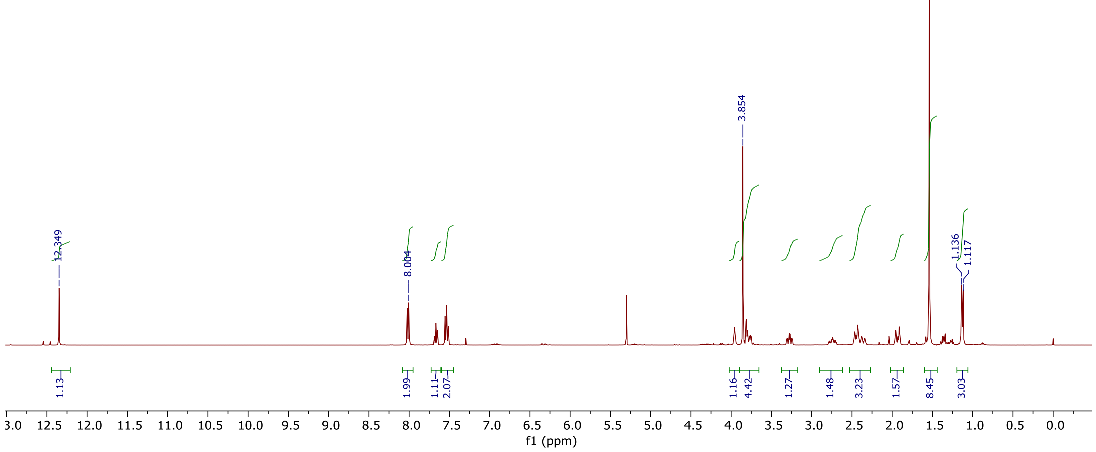

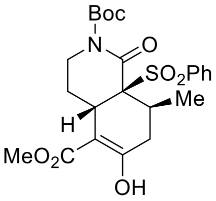

10a

(100.6 MHz, $CDCl_3$)

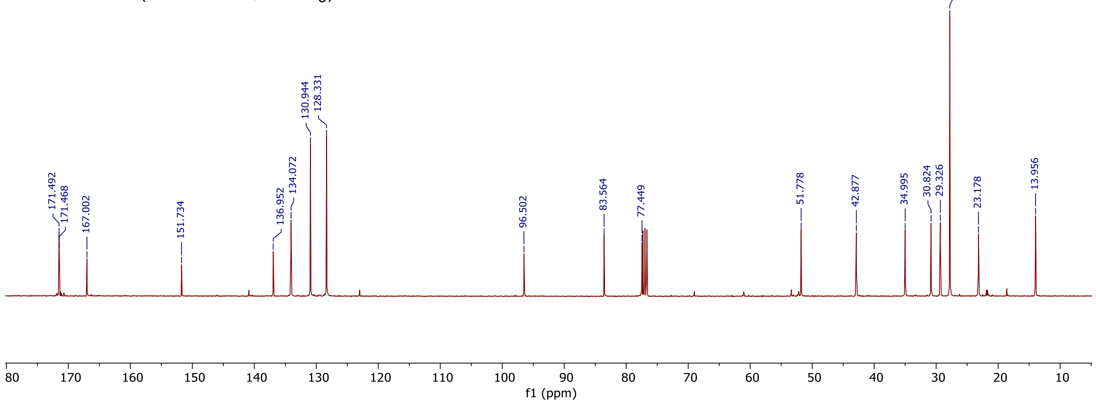

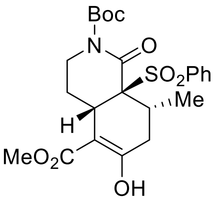

10b

(400 MHz, $CDCl_3$)

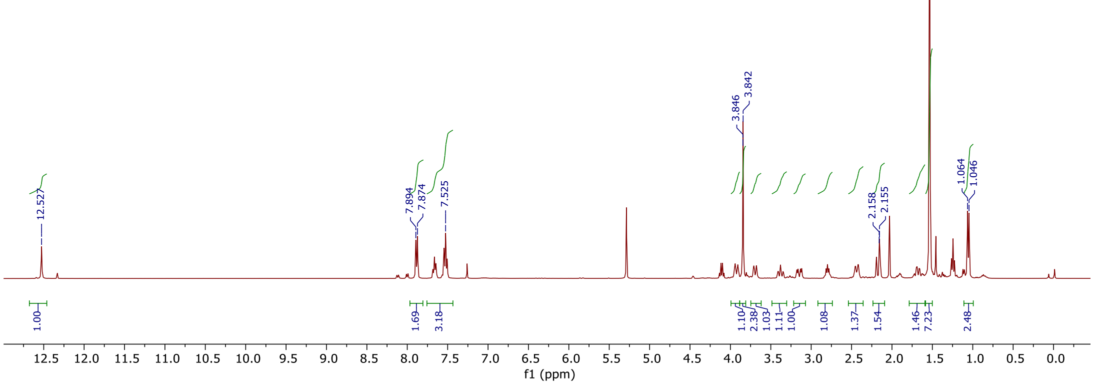

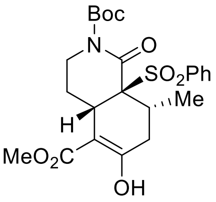

10b

(100.6 MHz, $CDCl_3$)

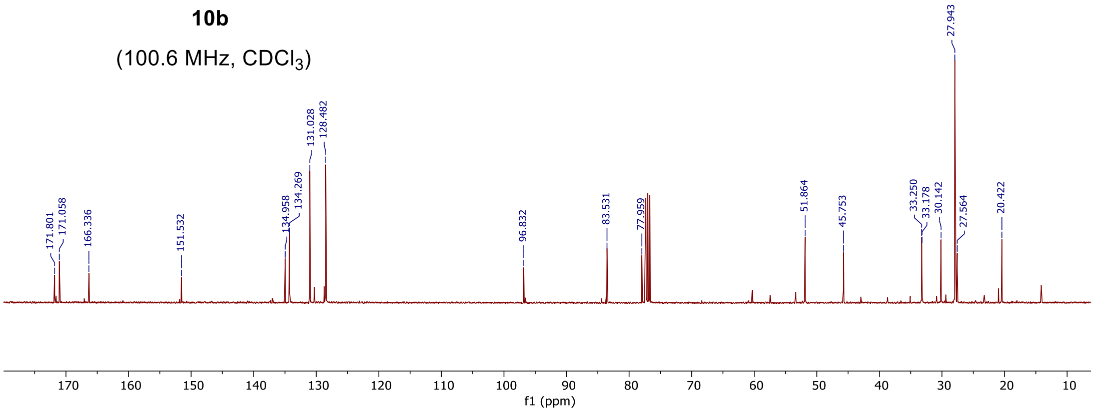

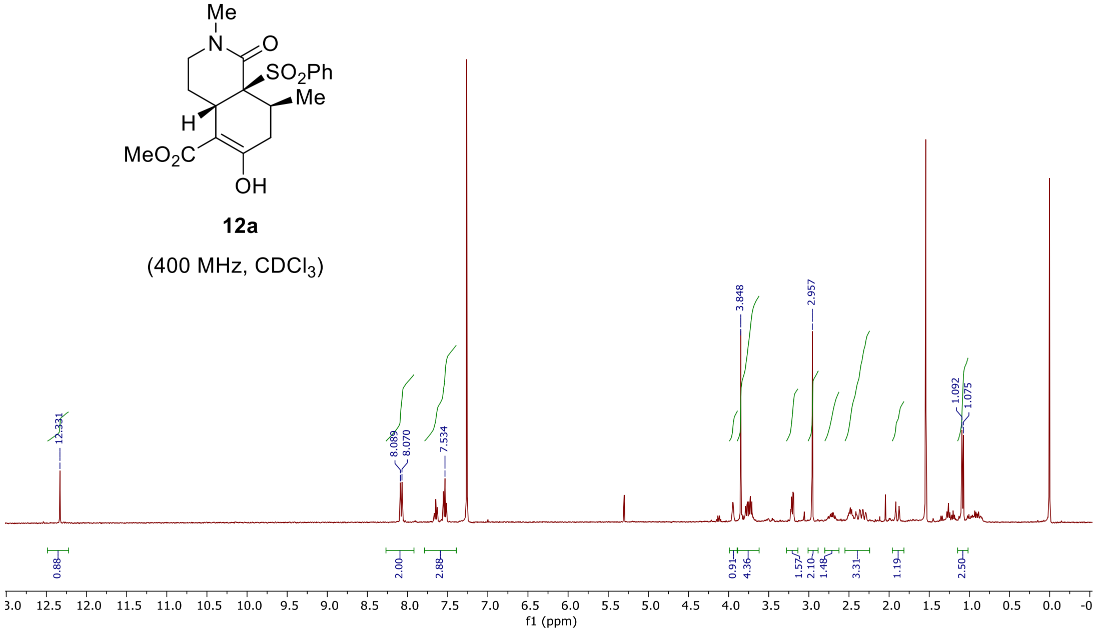

12a

(400 MHz, $CDCl_3$)

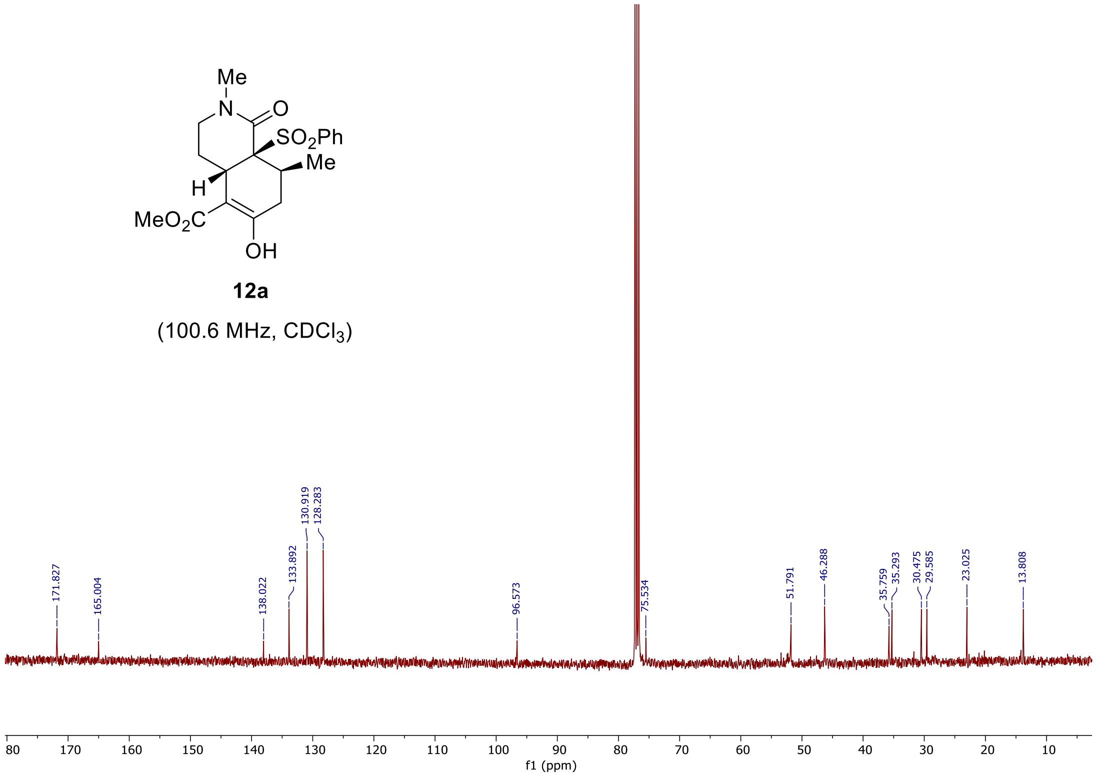

12a

(100.6 MHz, $CDCl_3$)

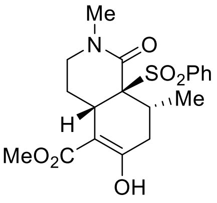

12b

(400 MHz, $CDCl_3$)

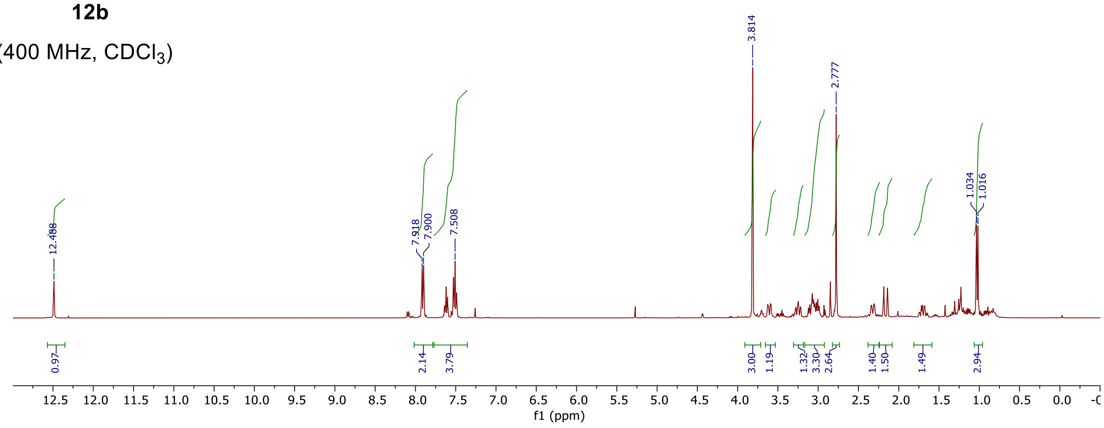

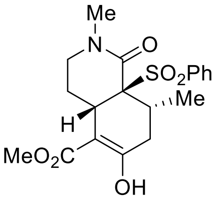

12b

(100.6 MHz, $CDCl_3$)

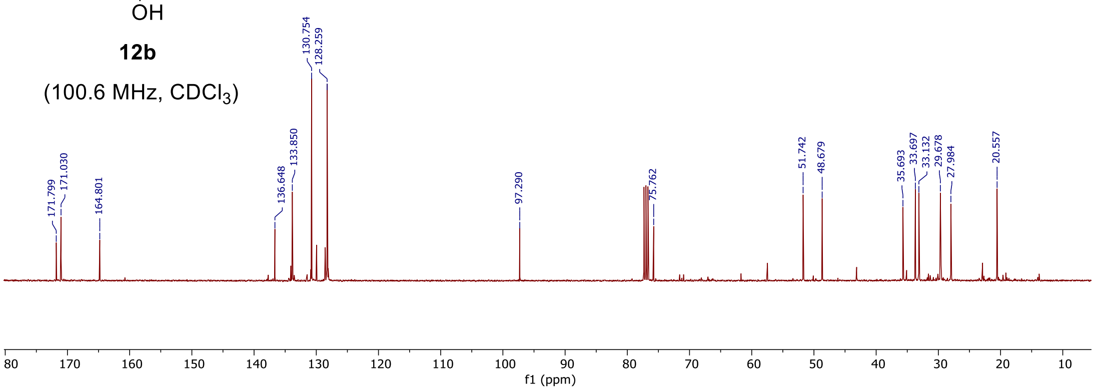

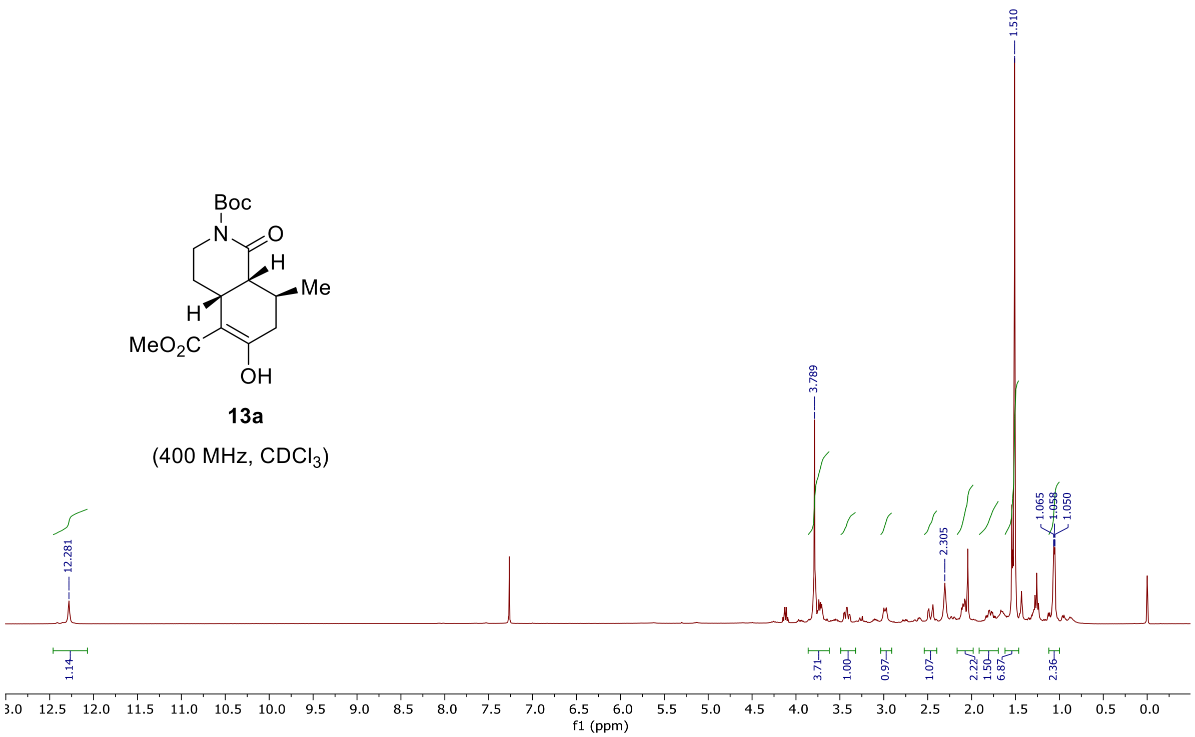
Boc
N
O
H
Me
H
MeO2C
OH
13a
(400 MHz, CDCl3)
12.281
3.789
2.305
1.510
1.065
1.058
1.050
1.14
3.71
1.00
0.97
1.07
2.22
1.50
6.87
2.36
f1 (ppm)

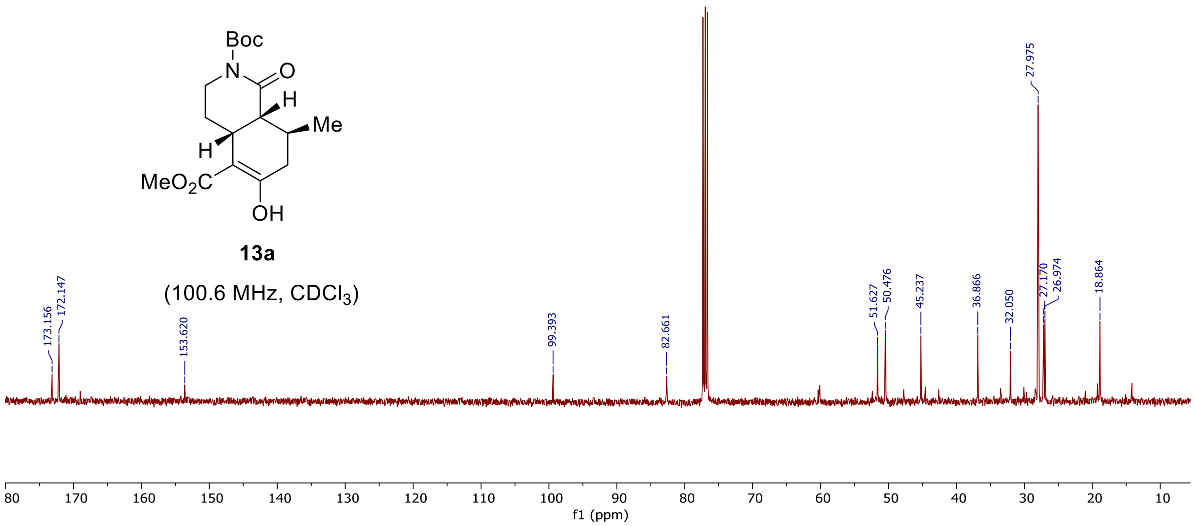
Boc
N
O
H
Me
H
MeO2C
OH
13a
(100.6 MHz, CDCl3)
173.156
172.147
153.620
99.393
82.661
51.627
50.476
45.237
36.866
32.050
27.975
27.170
26.974
18.864
f1 (ppm)

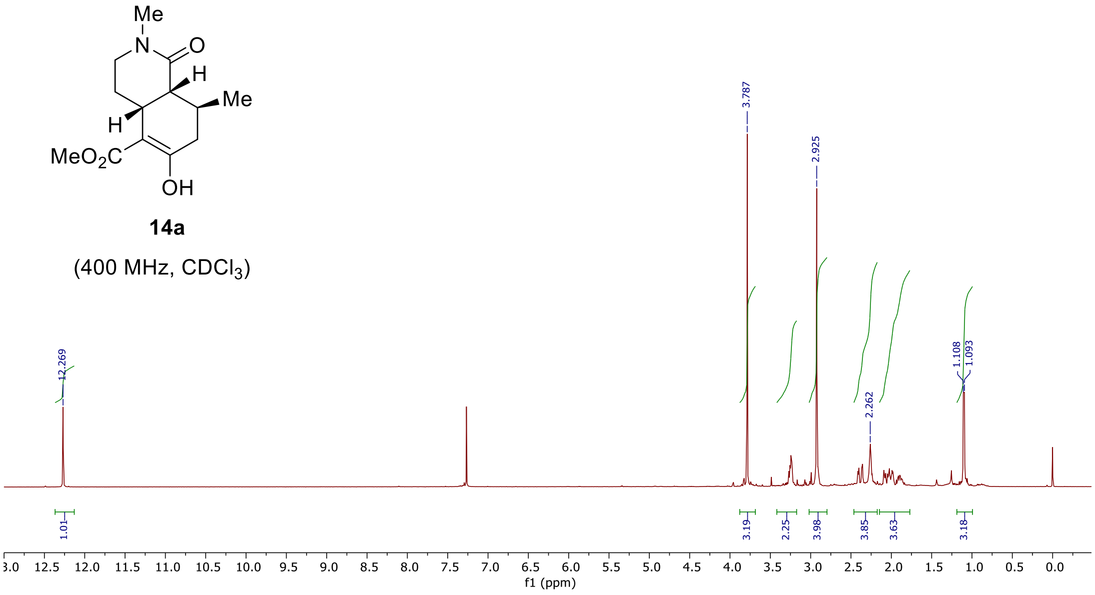

Me
N
O
H
Me
H
MeO2C
OH
14a
(400 MHz, CDCl3)
12.269
3.787
2.925
2.262
1.108
1.093
1.01
3.19
2.25
3.98
3.85
3.63
3.18
f1 (ppm)

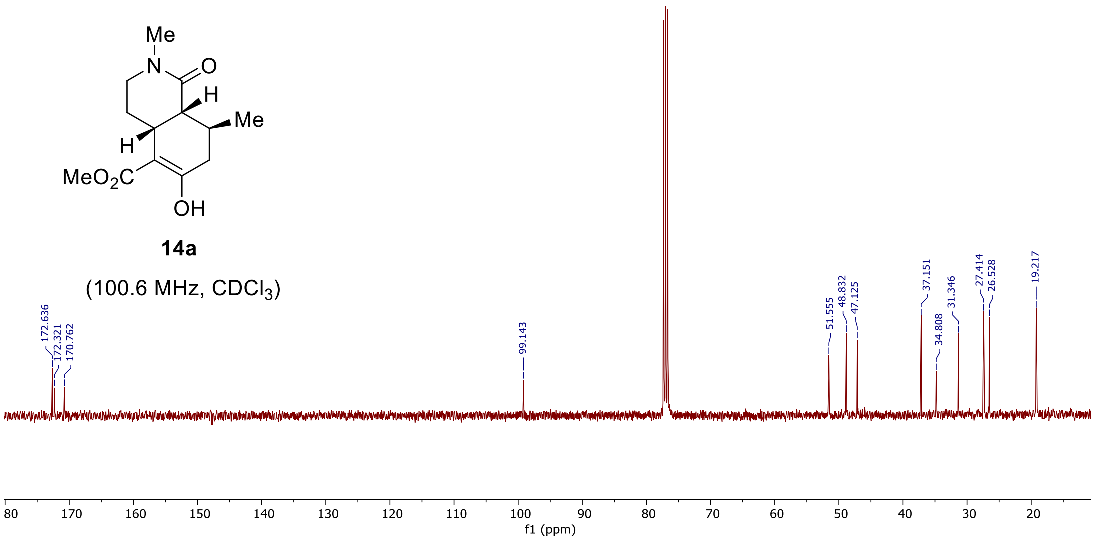

Me
N
O
H
Me
H
MeO2C
OH
14a
(100.6 MHz, CDCl3)
172.636
172.321
170.762
99.143
51.555
48.832
47.125
37.151
34.808
31.346
27.414
26.528
19.217
f1 (ppm)

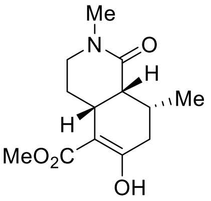

14b

(400 MHz, $CDCl_3$)

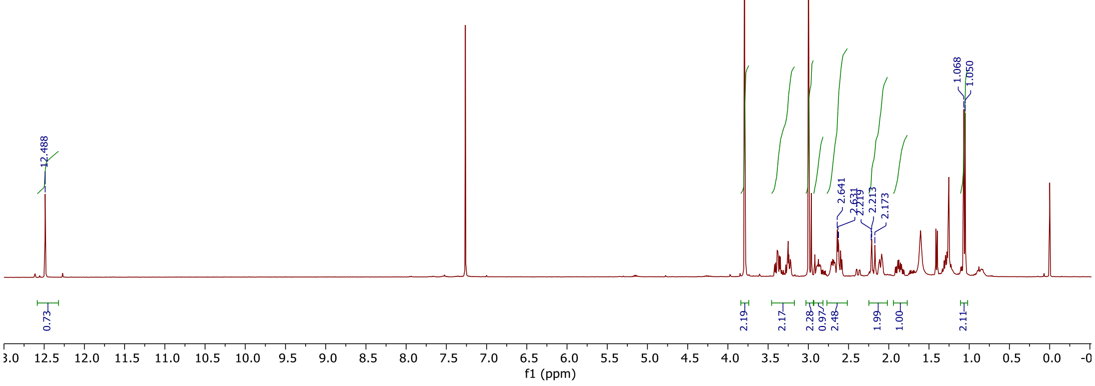

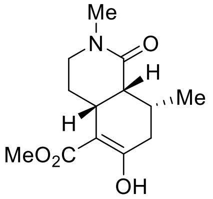

14b

(100.6 MHz, $CDCl_3$)

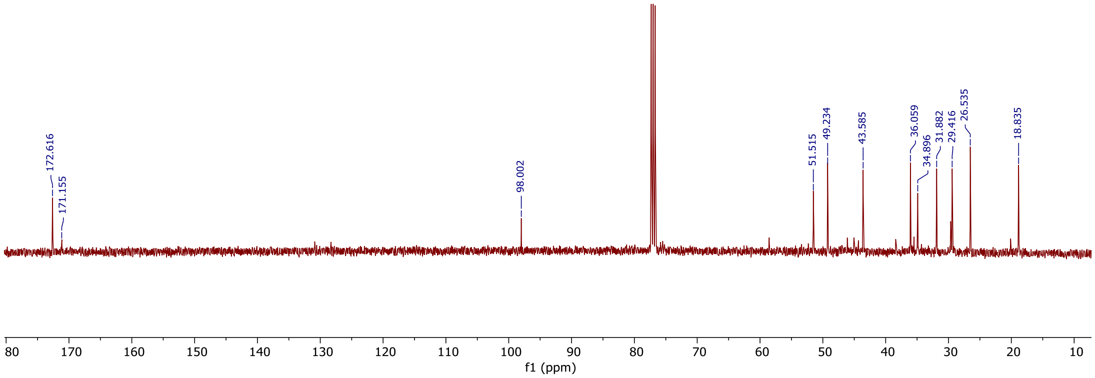

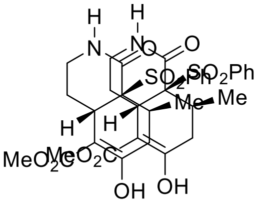

15

(400 MHz, $CDCl_3$)

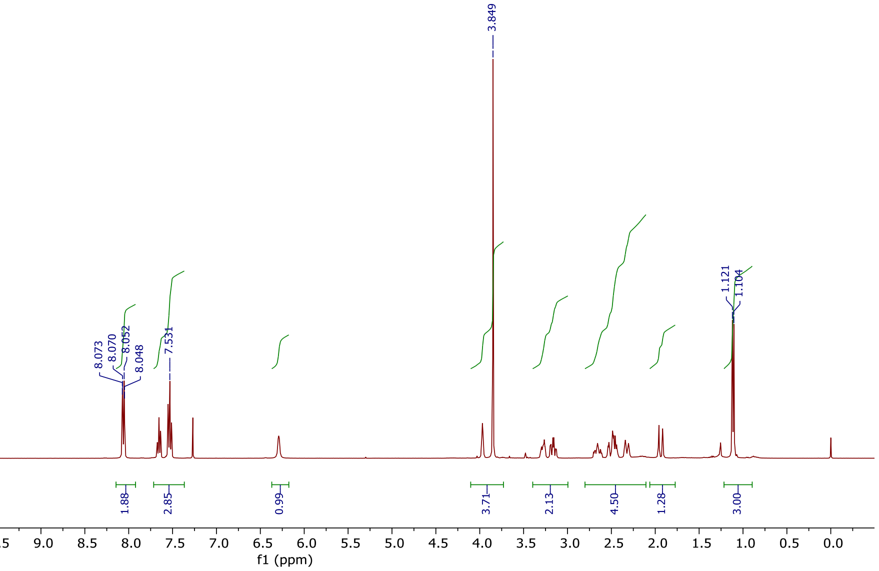

15

(100.6 MHz, $CDCl_3$)

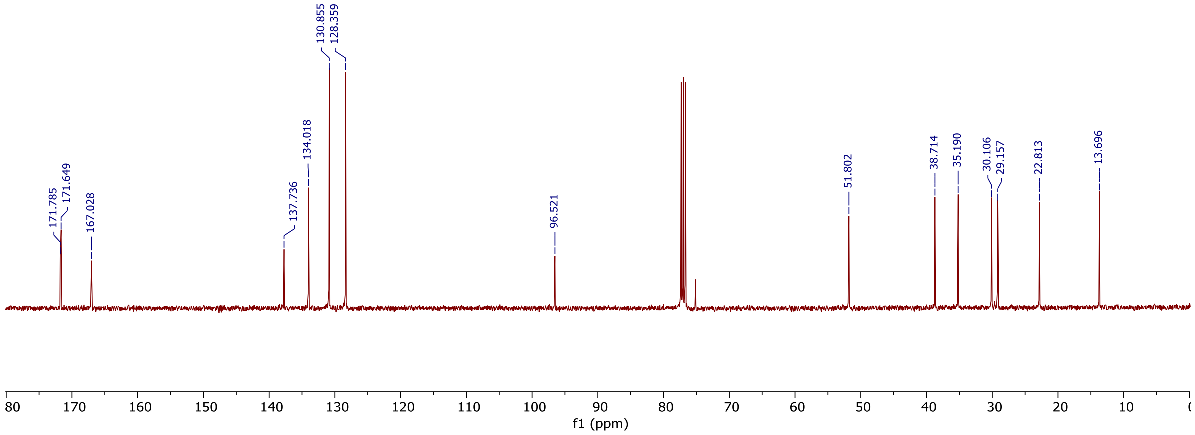

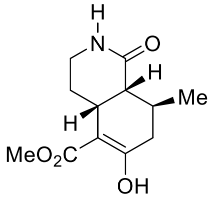

16

(400 MHz, $CDCl_3$)

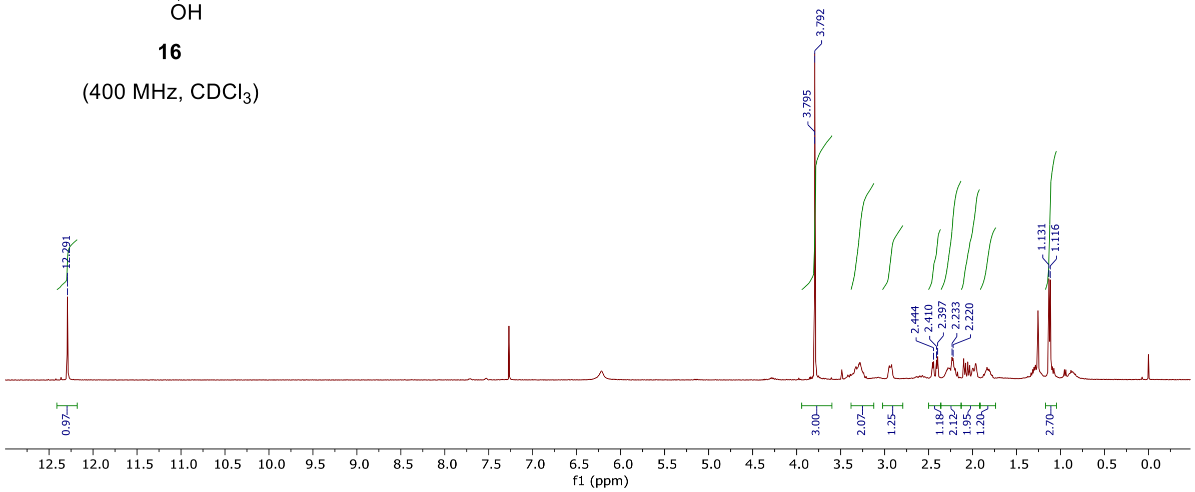

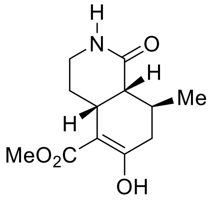

16

(100.6 MHz, $CDCl_3$)

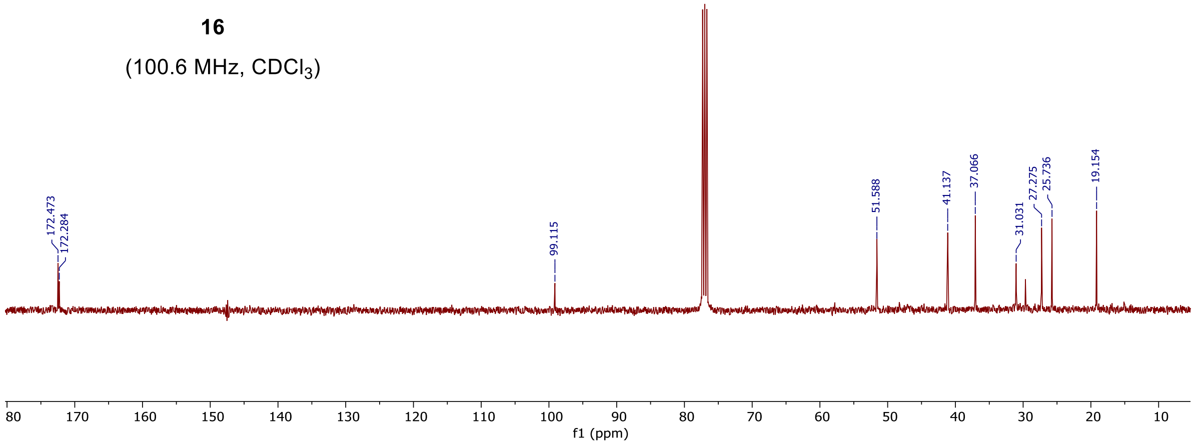

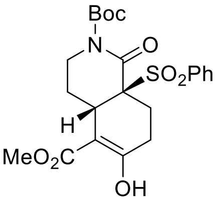

18a

(400 MHz, $CDCl_3$)

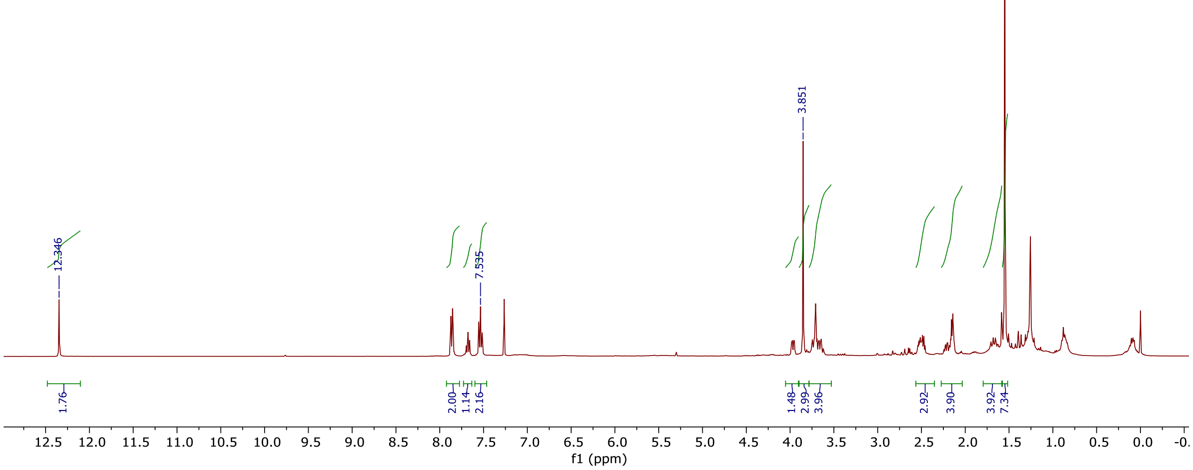

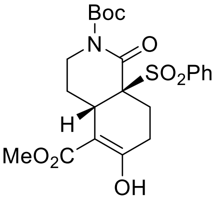

18a

(100.6 MHz, $CDCl_3$)

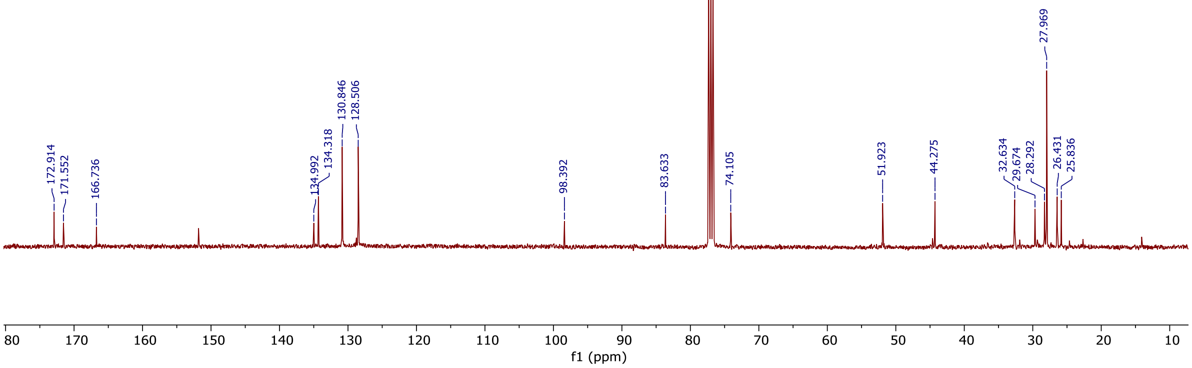

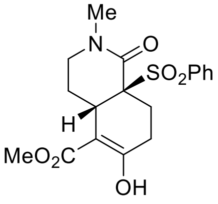

18b

(400 MHz, $CDCl_3$)

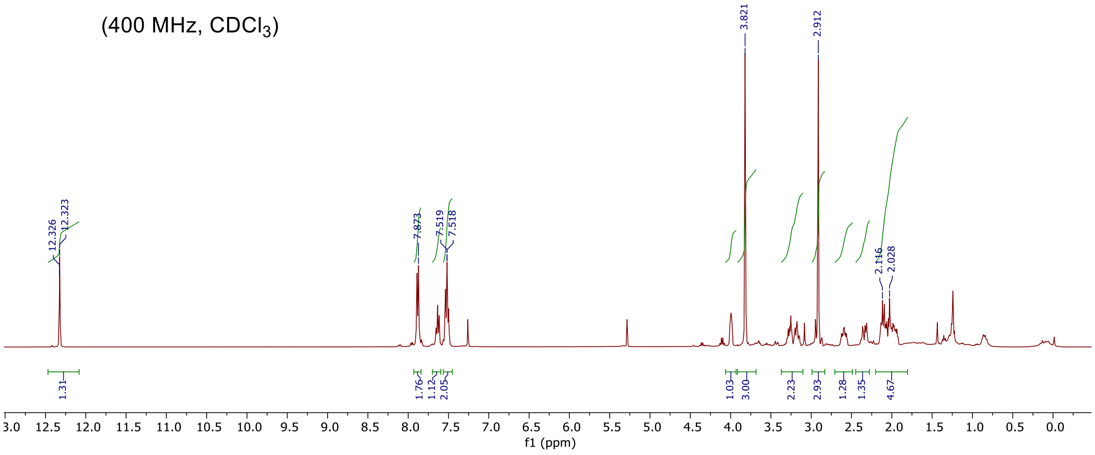

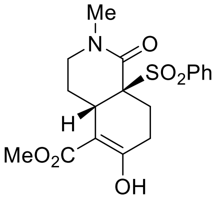

18b

(100.6 MHz, $CDCl_3$)

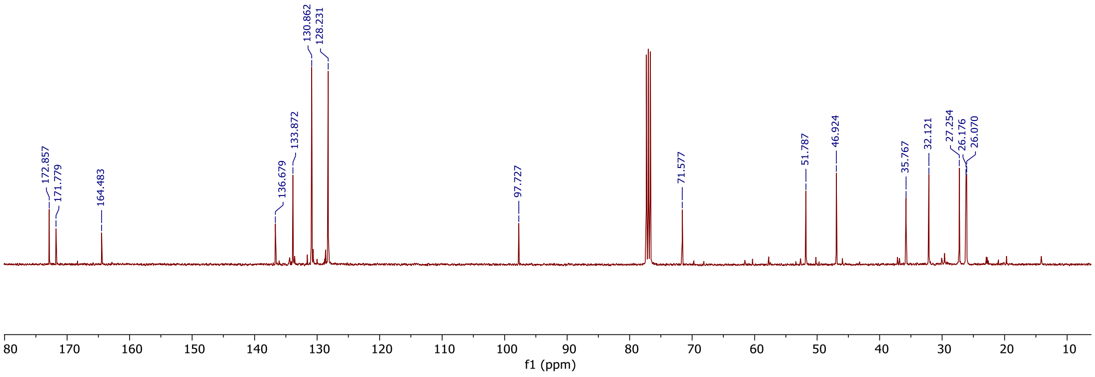

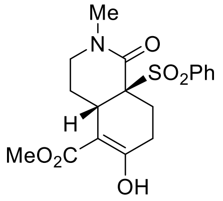

18c

(400 MHz, CDCl$_3$)

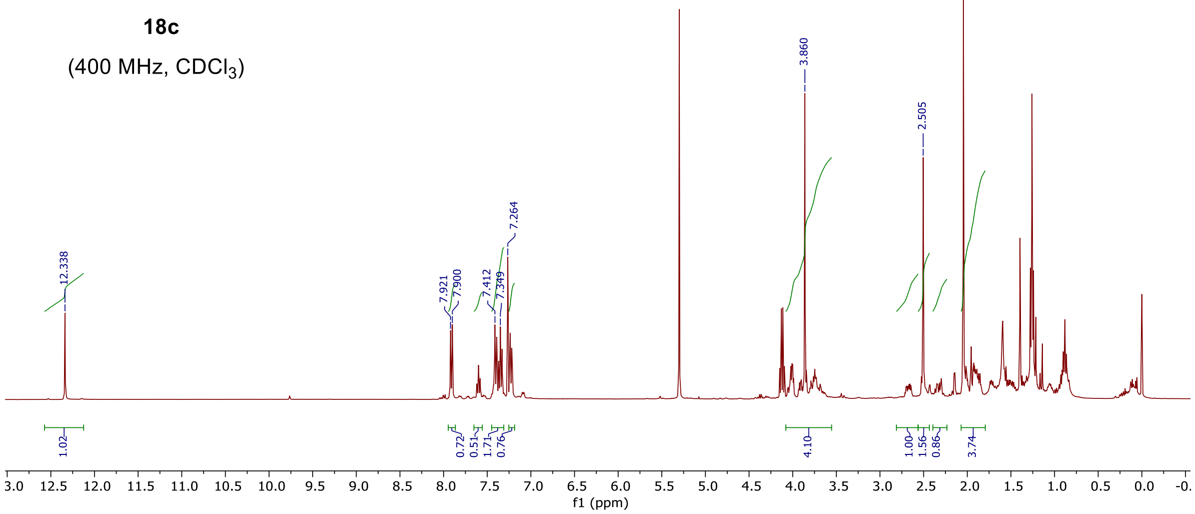

18c

(100.6 MHz, CDCl$_3$)

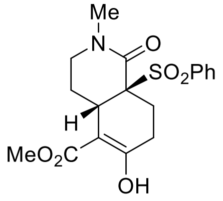

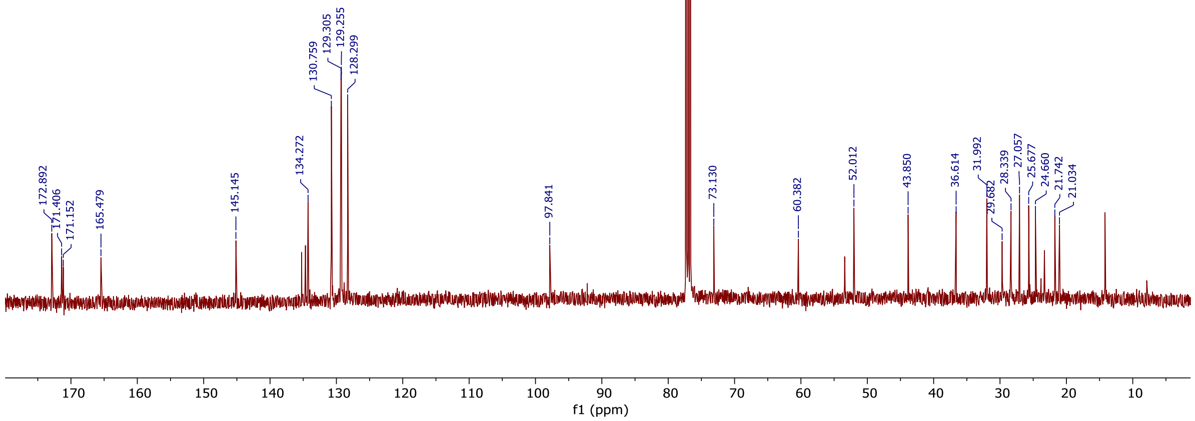

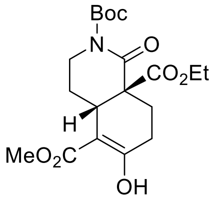

19

(400 MHz, $CDCl_3$)

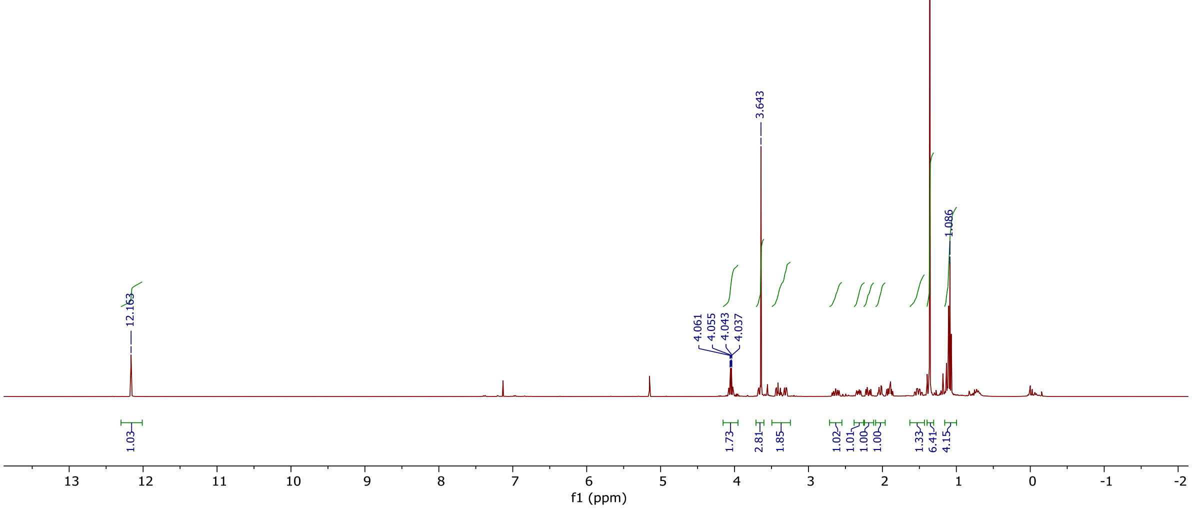

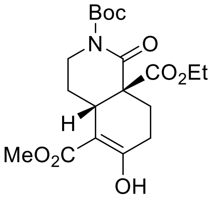

19

(100.6 MHz, $CDCl_3$)

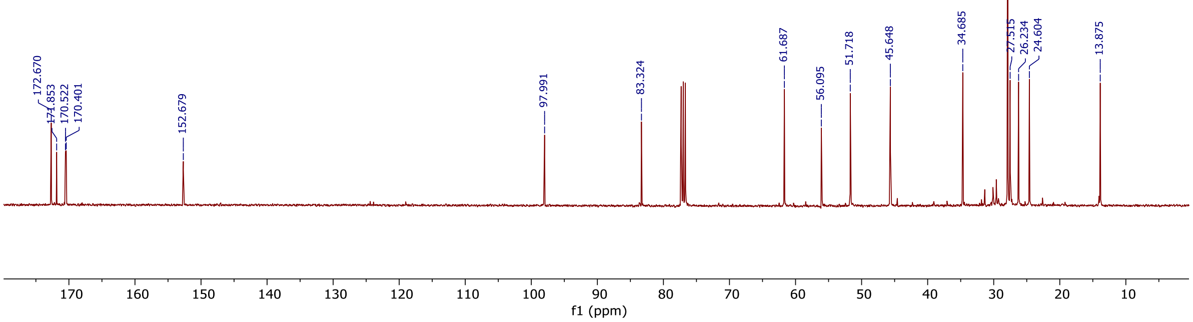

II) Copies of NOE NMR spectra **13a**, **14a**, and **14b**

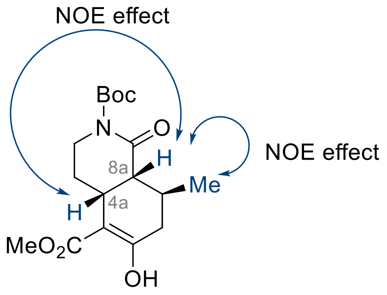

13a

(400 MHz, $CDCl_3$)

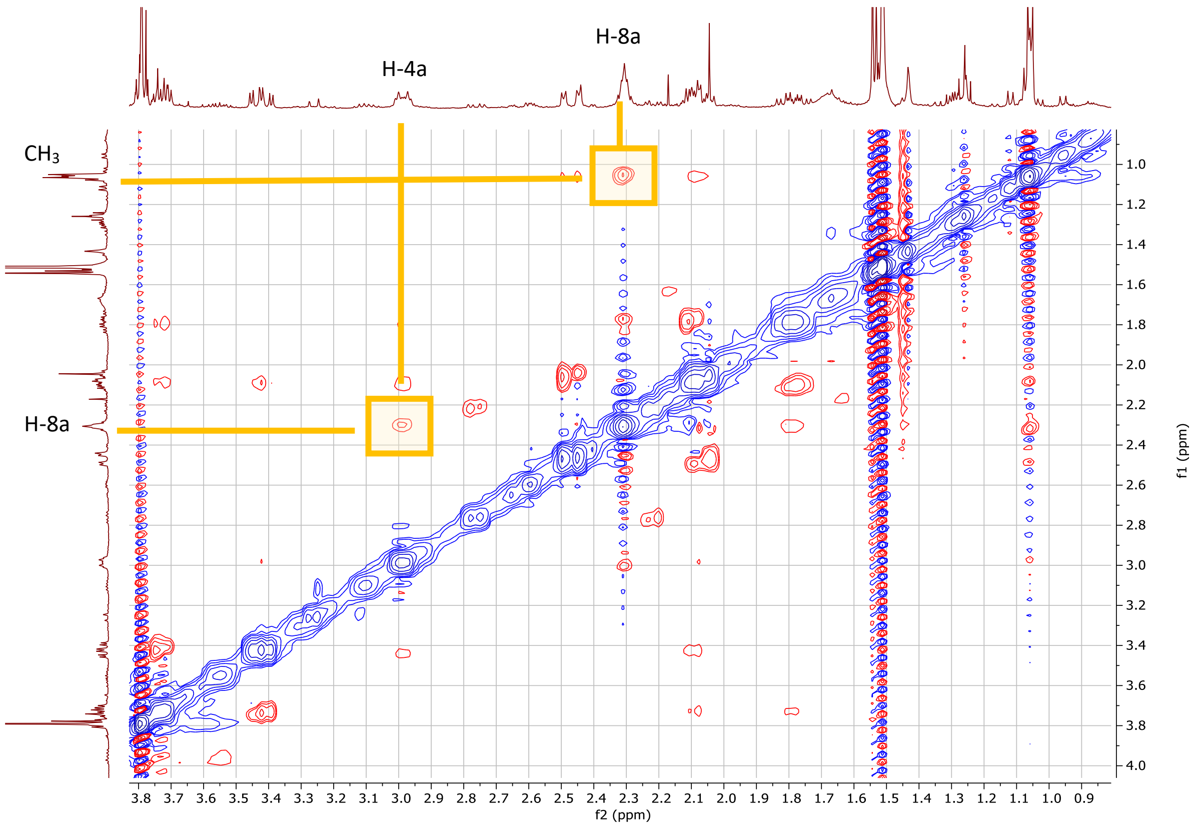

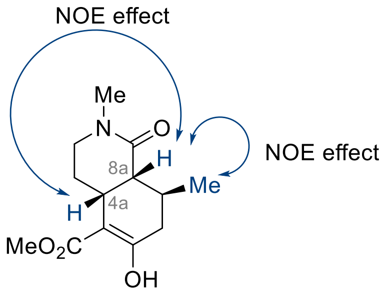

14a

(400 MHz, $CDCl_3$)

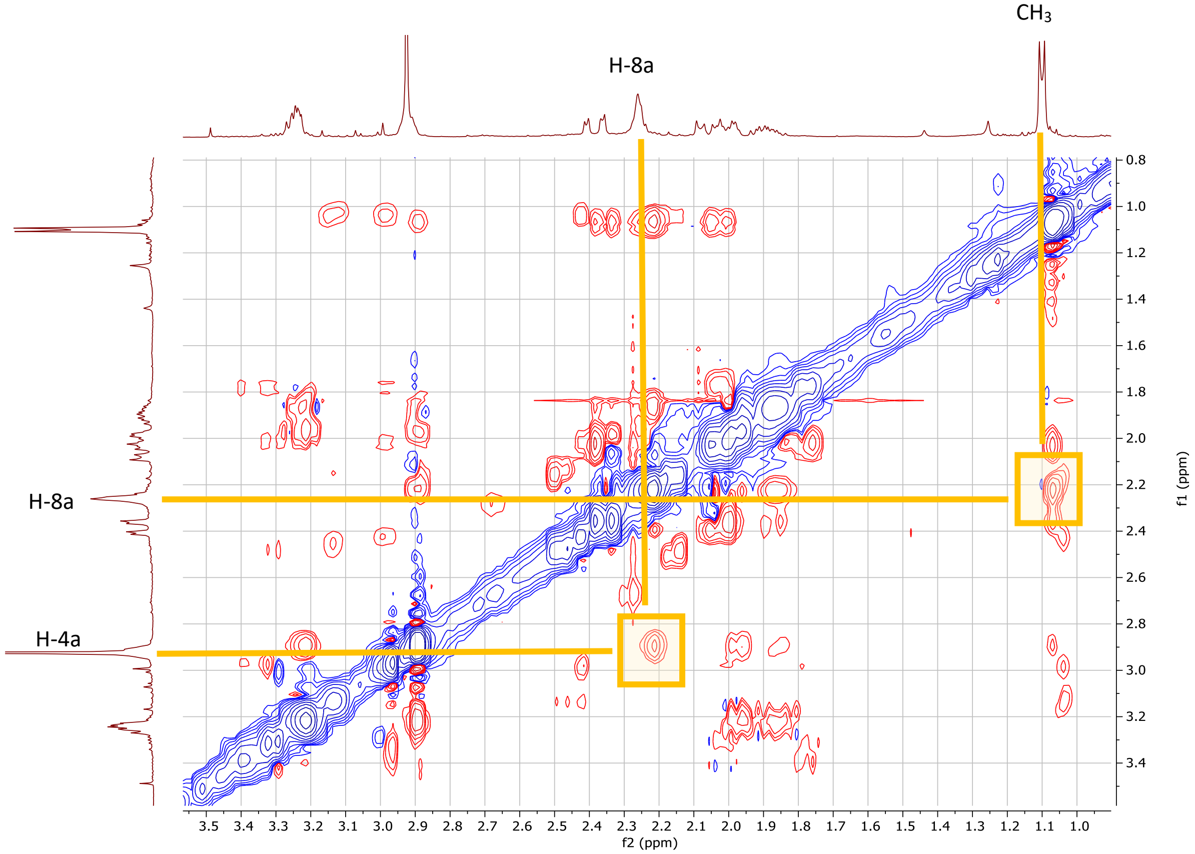

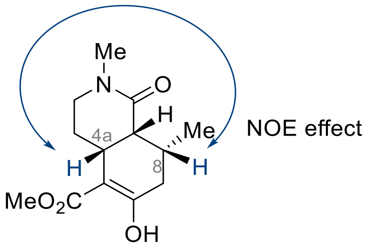

14b

(400 MHz, $CDCl_3$)

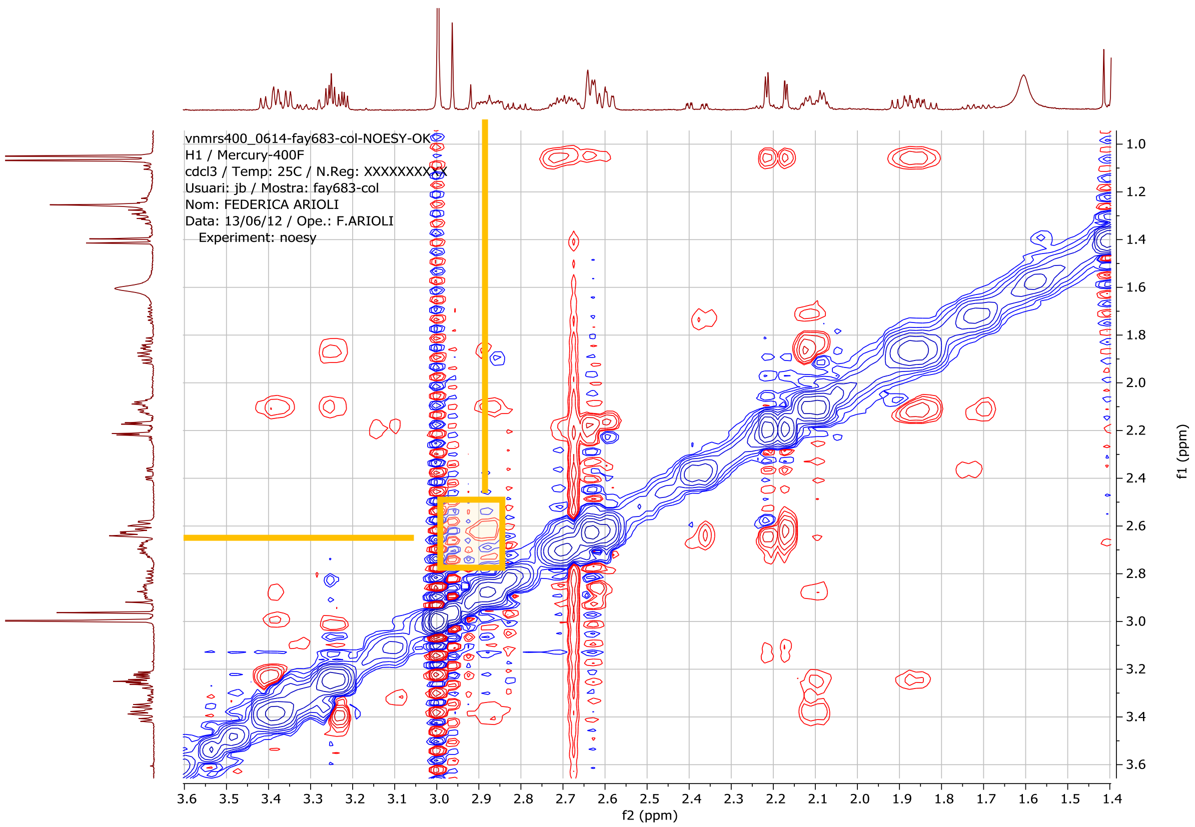

III) X- ray crystallographic data for compounds **10a**, **12a**, and **12b**

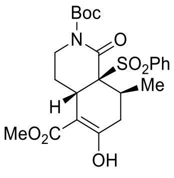

10a

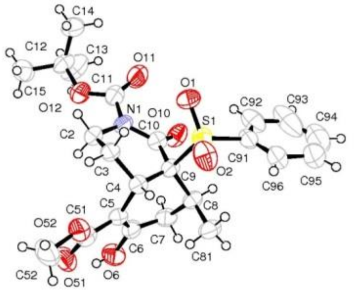

Table S1. Crystal data and structure refinement for compound **10a**.

Identification code	Jb95	
Empirical formula	C23 H29 N O8 S	
Formula weight	479.53	
Temperature	294(2) K	
Wavelength	0.71073 Å	
Crystal system	Orthorhombic	
Space group	P 21 21 21	
Unit cell dimensions	a = 7.948(3) Å	= 90°.
	b = 10.790(3) Å	= 90°.
	c = 27.749(9) Å	= 90°.
Volume	2379.7(14) $Å^3$	
Z	4	
Density (calculated)	1.338 Mg/m^3	
Absorption coefficient	0.184 mm^{-1}	
F(000)	1016	
Crystal size	0.45 x 0.27 x 0.15 mm^3	
Theta range for data collection	1.47 to 24.96°.	
Index ranges	0<=h<=9, 0<=k<=12, 0<=l<=32	
Reflections collected	2468	
Independent reflections	2398 [R(int) = 0.0534]	
Completeness to theta = 24.96°	99.8 %	
Max. and min. transmission	0.9729 and 0.9218	
Refinement method	Full-matrix least-squares on F^2	
Data / restraints / parameters	2398 / 0 / 304	
Goodness-of-fit on F^2	0.958	
Final R indices [I>2sigma(I)]	R1 = 0.0559, wR2 = 0.1214	
R indices (all data)	R1 = 0.1161, wR2 = 0.1470	
Absolute structure parameter	-0.1(2)	
Largest diff. peak and hole	0.220 and -0.256 e.$Å^{-3}$	

Table S2. Atomic coordinates (x 10^4) and equivalent isotropic displacement parameters (Å^{2}x 10^3) for jb95. U(eq) is defined as one third of the trace of the orthogonalized U^{ij} tensor.

	x	y	z	U(eq)
S(1)	6582(2)	1618(2)	4083(1)	57(1)
O(1)	8167(6)	1911(5)	3856(2)	75(2)
O(2)	6553(7)	1294(4)	4584(2)	76(2)
O(6)	1414(6)	5630(5)	4169(2)	79(2)
O(10)	5358(6)	3177(5)	3147(2)	68(2)
O(11)	8107(8)	4282(5)	2805(2)	89(2)
O(12)	8266(7)	6097(5)	3187(2)	70(1)
O(51)	2924(7)	6523(5)	4904(2)	80(2)
O(52)	4939(7)	5391(5)	5234(2)	68(1)
N(1)	6969(7)	4561(5)	3565(2)	53(2)
C(2)	7303(8)	5248(6)	4012(2)	53(2)
C(3)	7099(8)	4426(7)	4449(2)	53(2)
C(4)	5357(8)	3813(6)	4450(2)	46(2)
C(5)	3927(8)	4767(6)	4474(2)	47(2)
C(6)	2625(9)	4752(7)	4167(3)	58(2)
C(7)	2348(8)	3766(7)	3807(3)	60(2)
C(8)	3301(8)	2602(6)	3921(2)	48(2)
C(9)	5178(8)	2995(6)	4001(2)	45(2)
C(10)	5832(9)	3587(6)	3526(3)	51(2)
C(11)	7818(10)	4960(7)	3146(3)	62(2)
C(12)	9343(9)	6741(7)	2826(3)	62(2)
C(13)	8442(11)	6864(8)	2359(3)	99(3)
C(14)	11002(9)	6081(7)	2780(3)	77(2)
C(15)	9630(11)	7999(7)	3075(3)	85(3)
C(51)	3888(9)	5628(7)	4873(3)	59(2)
C(52)	4860(11)	6166(8)	5662(3)	94(3)
C(81)	2539(9)	1939(6)	4356(3)	62(2)
C(91)	5719(9)	355(7)	3758(3)	56(2)
C(92)	5774(11)	357(8)	3271(3)	80(3)
C(93)	5130(16)	-661(12)	3024(5)	127(5)
C(94)	4492(14)	-1639(14)	3279(7)	134(7)
C(95)	4475(14)	-1643(11)	3753(5)	123(5)
C(96)	5069(11)	-601(8)	4020(4)	92(3)

Table S3. Bond lengths [Å] and angles [°] for jb95.

S(1)-O(2)	1.436(5)
S(1)-O(1)	1.444(5)
S(1)-C(91)	1.771(7)
S(1)-C(9)	1.871(6)
O(6)-C(6)	1.350(8)
O(10)-C(10)	1.204(7)
O(11)-C(11)	1.218(8)
O(12)-C(11)	1.282(8)
O(12)-C(12)	1.488(8)
O(51)-C(51)	1.236(8)
O(52)-C(51)	1.329(8)
O(52)-C(52)	1.453(8)
N(1)-C(10)	1.391(8)
N(1)-C(11)	1.412(9)
N(1)-C(2)	1.469(7)
C(2)-C(3)	1.509(8)
C(3)-C(4)	1.534(9)
C(4)-C(9)	1.534(9)
C(4)-C(5)	1.535(9)
C(5)-C(6)	1.341(9)
C(5)-C(51)	1.446(9)
C(6)-C(7)	1.475(9)
C(7)-C(8)	1.500(9)
C(8)-C(81)	1.528(8)
C(8)-C(9)	1.567(8)
C(9)-C(10)	1.553(9)
C(12)-C(13)	1.488(10)
C(12)-C(14)	1.504(10)
C(12)-C(15)	1.540(10)
C(91)-C(92)	1.352(10)
C(91)-C(96)	1.364(11)
C(92)-C(93)	1.392(13)
C(93)-C(94)	1.368(18)
C(94)-C(95)	1.315(17)
C(95)-C(96)	1.427(14)
O(2)-S(1)-O(1)	119.4(3)
O(2)-S(1)-C(91)	107.5(4)
O(1)-S(1)-C(91)	106.5(3)
O(2)-S(1)-C(9)	107.5(3)
O(1)-S(1)-C(9)	107.0(3)
C(91)-S(1)-C(9)	108.6(3)
C(11)-O(12)-C(12)	123.2(6)
C(51)-O(52)-C(52)	118.5(6)
C(10)-N(1)-C(11)	118.5(6)

Table S3. *Cont*.

C(10)-N(1)-C(2)	124.4(6)
C(11)-N(1)-C(2)	117.1(6)
N(1)-C(2)-C(3)	111.2(5)
C(2)-C(3)-C(4)	110.7(5)
C(9)-C(4)-C(3)	109.2(5)
C(9)-C(4)-C(5)	110.7(5)
C(3)-C(4)-C(5)	112.3(5)
C(6)-C(5)-C(51)	118.6(6)
C(6)-C(5)-C(4)	122.4(6)
C(51)-C(5)-C(4)	118.7(6)
C(5)-C(6)-O(6)	122.6(7)
C(5)-C(6)-C(7)	123.6(7)
O(6)-C(6)-C(7)	113.8(6)
C(6)-C(7)-C(8)	112.7(6)
C(7)-C(8)-C(81)	111.0(6)
C(7)-C(8)-C(9)	106.5(5)
C(81)-C(8)-C(9)	113.1(5)
C(4)-C(9)-C(10)	114.9(5)
C(4)-C(9)-C(8)	111.0(5)
C(10)-C(9)-C(8)	108.1(5)
C(4)-C(9)-S(1)	107.7(4)
C(10)-C(9)-S(1)	103.3(4)
C(8)-C(9)-S(1)	111.7(4)
O(10)-C(10)-N(1)	123.3(6)
O(10)-C(10)-C(9)	119.1(6)
N(1)-C(10)-C(9)	117.6(6)
O(11)-C(11)-O(12)	126.2(8)
O(11)-C(11)-N(1)	123.2(7)
O(12)-C(11)-N(1)	110.6(7)
C(13)-C(12)-O(12)	110.5(6)
C(13)-C(12)-C(14)	112.9(7)
O(12)-C(12)-C(14)	109.9(6)
C(13)-C(12)-C(15)	112.5(7)
O(12)-C(12)-C(15)	101.3(6)
C(14)-C(12)-C(15)	109.0(6)
O(51)-C(51)-O(52)	119.2(7)
O(51)-C(51)-C(5)	124.6(7)
O(52)-C(51)-C(5)	116.1(7)
C(92)-C(91)-C(96)	123.1(8)
C(92)-C(91)-S(1)	119.6(7)
C(96)-C(91)-S(1)	117.2(7)
C(91)-C(92)-C(93)	118.6(10)
C(94)-C(93)-C(92)	119.4(13)
C(95)-C(94)-C(93)	121.5(15)

Table S3. *Cont*.

C(94)-C(95)-C(96)	120.9(14)
C(91)-C(96)-C(95)	116.4(10)

Table S4. Anisotropic displacement parameters ($Å^2$x 10^3)for jb95. The anisotropic displacement factor exponent takes the form: -2 ^{2}[$h^2a^{*2}U^{11}$ + ... + 2 h k a* b* U^{12}]

	U^{11}	U^{22}	U^{33}	U^{23}	U^{13}	U^{12}
S(1)	51(1)	53(1)	66(1)	-1(1)	-10(1)	4(1)
O(1)	37(3)	78(4)	111(4)	-9(3)	2(3)	-2(3)
O(2)	96(4)	60(3)	72(3)	5(3)	-26(3)	18(3)
O(6)	43(3)	69(4)	125(5)	-7(3)	-6(3)	22(3)
O(10)	71(3)	77(4)	56(3)	2(3)	-7(3)	-29(3)
O(11)	93(4)	81(4)	94(4)	-14(3)	31(4)	-31(4)
O(12)	71(3)	55(3)	82(3)	10(3)	15(3)	-10(3)
O(51)	72(4)	63(3)	106(4)	-23(3)	-1(3)	13(3)
O(52)	65(3)	66(3)	72(3)	-17(3)	3(3)	-4(3)
N(1)	46(3)	61(4)	53(3)	-5(3)	6(3)	-13(3)
C(2)	44(4)	55(4)	60(5)	-3(4)	-2(4)	-7(4)
C(3)	38(4)	55(4)	67(5)	-10(4)	-3(3)	0(4)
C(4)	45(4)	46(4)	46(4)	5(3)	-2(3)	1(3)
C(5)	38(4)	50(4)	52(4)	-2(3)	3(3)	1(3)
C(6)	38(4)	52(5)	85(6)	-2(4)	1(4)	-2(4)
C(7)	33(4)	67(5)	78(5)	1(4)	-6(4)	7(4)
C(8)	35(3)	57(4)	52(4)	5(3)	-6(3)	-5(4)
C(9)	36(3)	43(4)	56(4)	14(3)	-5(3)	2(3)
C(10)	51(4)	54(5)	50(4)	4(4)	-2(4)	1(4)
C(11)	60(5)	60(5)	67(5)	-2(4)	2(4)	-9(4)
C(12)	52(4)	66(5)	68(5)	18(4)	3(4)	-12(5)
C(13)	93(6)	128(8)	76(5)	38(6)	-11(6)	-32(7)
C(14)	51(5)	79(6)	100(6)	0(5)	3(5)	3(5)
C(15)	89(6)	56(5)	109(7)	6(5)	26(6)	6(5)
C(51)	48(4)	62(5)	69(5)	2(4)	-1(4)	-8(4)
C(52)	82(6)	97(7)	105(7)	-48(6)	-6(6)	4(6)
C(81)	43(4)	67(5)	75(5)	10(4)	5(4)	-3(4)
C(91)	49(4)	52(5)	67(5)	-15(4)	-7(4)	6(4)
C(92)	79(6)	75(6)	86(6)	-9(5)	-4(5)	29(5)
C(93)	111(10)	124(10)	147(11)	-69(10)	-56(9)	58(9)
C(94)	63(7)	97(10)	242(19)	-73(14)	-36(11)	11(7)
C(95)	80(7)	68(7)	221(15)	-22(10)	28(10)	0(6)
C(96)	74(6)	58(5)	145(9)	-25(6)	21(7)	4(5)

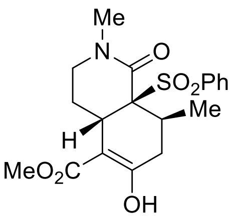

12a

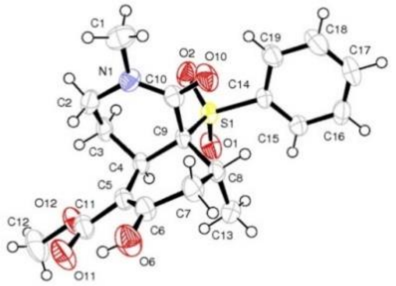

Table S5. Crystal data and structure refinement for compound **12a**.

Identification code	Jb94	
Empirical formula	C19 H23 N O6 S	
Formula weight	393.44	
Temperature	293(2) K	
Wavelength	0.71073 Å	
Crystal system	Triclinic	
Space group	P -1	
Unit cell dimensions	a = 7.652(3) Å	= 80.94(4)°.
	b = 8.175(3) Å	= 79.28(4)°.
	c = 17.270(10) Å	= 62.69(4)°.
Volume	939.9(7) Å^3	
Z	2	
Density (calculated)	1.390 Mg/m^3	
Absorption coefficient	0.208 mm^{-1}	
F(000)	416	
Crystal size	0.45 x 0.39 x 0.21 mm^3	
Theta range for data collection	1.20 to 24.97°.	
Index ranges	-8<=h<=9, -9<=k<=9, 0<=l<=20	
Reflections collected	3556	
Independent reflections	3294 [R(int) = 0.0452]	
Completeness to theta = 24.97°	100.0 %	
Refinement method	Full-matrix least-squares on F^2	
Data / restraints / parameters	3294 / 0 / 248	
Goodness-of-fit on F^2	1.054	
Final R indices [I>2sigma(I)]	R1 = 0.0451, wR2 = 0.1184	
R indices (all data)	R1 = 0.0607, wR2 = 0.1255	
Largest diff. peak and hole	0.314 and -0.267 e.Å^{-3}	

Table S6. Atomic coordinates (x 10^4) and equivalent isotropic displacement parameters ($Å^2$x 10^3) for jb94. U(eq) is defined as one third of the trace of the orthogonalized U^{ij} tensor.

	x	y	z	U(eq)
S(1)	3709(1)	3363(1)	2117(1)	36(1)
O(1)	3669(3)	4156(2)	2806(1)	51(1)
O(2)	5595(2)	2370(2)	1661(1)	54(1)
O(6)	-656(3)	-921(3)	3763(1)	65(1)
O(10)	2083(3)	1508(2)	1128(1)	53(1)
O(11)	1981(3)	-2493(3)	4700(1)	70(1)
O(12)	4294(3)	-1486(2)	4591(1)	56(1)
N(1)	4416(3)	-1052(3)	1677(1)	41(1)
C(1)	4852(4)	-1984(4)	963(2)	57(1)
C(2)	5500(3)	-2140(3)	2337(2)	44(1)
C(3)	5560(3)	-933(3)	2893(1)	41(1)
C(4)	3466(3)	526(3)	3162(1)	34(1)
C(5)	2184(3)	-372(3)	3618(1)	38(1)
C(6)	434(4)	-59(3)	3406(2)	44(1)
C(7)	-535(4)	1385(4)	2776(2)	48(1)
C(8)	266(3)	2822(3)	2617(1)	39(1)
C(9)	2551(3)	1748(3)	2428(1)	32(1)
C(10)	3009(3)	704(3)	1690(1)	37(1)
C(11)	2787(4)	-1558(3)	4340(2)	47(1)
C(12)	5003(5)	-2610(4)	5303(2)	77(1)
C(13)	-457(4)	4021(3)	3316(2)	52(1)
C(14)	2222(3)	5159(3)	1466(1)	34(1)
C(15)	820(3)	6827(3)	1746(1)	39(1)
C(16)	-290(4)	8206(3)	1218(2)	48(1)
C(17)	-6(4)	7926(3)	432(2)	52(1)
C(18)	1433(5)	6285(4)	157(2)	62(1)
C(19)	2573(4)	4898(3)	672(2)	51(1)

Table S7. Bond lengths [Å] and angles [°] for jb94.

S(1)-O(1)	1.4321(19)
S(1)-O(2)	1.437(2)
S(1)-C(14)	1.771(2)
S(1)-C(9)	1.868(2)
O(6)-C(6)	1.335(3)
O(10)-C(10)	1.226(3)
O(11)-C(11)	1.222(3)
O(12)-C(11)	1.333(3)
O(12)-C(12)	1.444(3)
N(1)-C(10)	1.343(3)
N(1)-C(1)	1.456(3)
N(1)-C(2)	1.462(3)
C(2)-C(3)	1.501(3)
C(3)-C(4)	1.533(3)
C(4)-C(5)	1.521(3)
C(4)-C(9)	1.545(3)
C(5)-C(6)	1.354(3)
C(5)-C(11)	1.459(3)
C(6)-C(7)	1.489(3)
C(7)-C(8)	1.525(3)
C(8)-C(13)	1.533(4)
C(8)-C(9)	1.551(3)
C(9)-C(10)	1.540(3)
C(14)-C(19)	1.378(3)
C(14)-C(15)	1.385(3)
C(15)-C(16)	1.382(3)
C(16)-C(17)	1.374(4)
C(17)-C(18)	1.374(4)
C(18)-C(19)	1.379(4)
O(1)-S(1)-O(2)	118.30(12)
O(1)-S(1)-C(14)	108.32(11)
O(2)-S(1)-C(14)	106.63(11)
O(1)-S(1)-C(9)	107.98(10)
O(2)-S(1)-C(9)	107.41(11)
C(14)-S(1)-C(9)	107.79(10)
C(11)-O(12)-C(12)	117.5(2)
C(10)-N(1)-C(1)	118.1(2)
C(10)-N(1)-C(2)	125.01(19)
C(1)-N(1)-C(2)	116.9(2)
N(1)-C(2)-C(3)	111.79(18)
C(2)-C(3)-C(4)	110.79(18)
C(5)-C(4)-C(3)	111.12(18)
C(5)-C(4)-C(9)	111.53(17)
C(3)-C(4)-C(9)	108.73(18)

Table S7. *Cont.*

C(6)-C(5)-C(11)	117.4(2)
C(6)-C(5)-C(4)	122.8(2)
C(11)-C(5)-C(4)	119.7(2)
O(6)-C(6)-C(5)	124.1(2)
O(6)-C(6)-C(7)	113.0(2)
C(5)-C(6)-C(7)	122.8(2)
C(6)-C(7)-C(8)	111.30(19)
C(7)-C(8)-C(13)	110.2(2)
C(7)-C(8)-C(9)	106.62(18)
C(13)-C(8)-C(9)	115.0(2)
C(10)-C(9)-C(4)	114.97(17)
C(10)-C(9)-C(8)	107.96(18)
C(4)-C(9)-C(8)	110.73(18)
C(10)-C(9)-S(1)	104.17(15)
C(4)-C(9)-S(1)	107.67(14)
C(8)-C(9)-S(1)	111.17(15)
O(10)-C(10)-N(1)	121.7(2)
O(10)-C(10)-C(9)	118.9(2)
N(1)-C(10)-C(9)	119.39(19)
O(11)-C(11)-O(12)	122.3(2)
O(11)-C(11)-C(5)	124.5(2)
O(12)-C(11)-C(5)	113.2(2)
C(19)-C(14)-C(15)	121.0(2)
C(19)-C(14)-S(1)	118.64(18)
C(15)-C(14)-S(1)	120.24(18)
C(16)-C(15)-C(14)	118.6(2)
C(17)-C(16)-C(15)	120.6(2)
C(16)-C(17)-C(18)	120.2(2)
C(17)-C(18)-C(19)	120.1(3)
C(14)-C(19)-C(18)	119.4(2)

Table S8. Anisotropic displacement parameters ($Å^2$x 10^3)for jb94. The anisotropic displacement factor exponent takes the form: -2 ^{2}[$h^2a^{*2}U^{11}$ + ... + 2 h k a* b* U^{12}]

	U^{11}	U^{22}	U^{33}	U^{23}	U^{13}	U^{12}
S(1)	36(1)	30(1)	42(1)	4(1)	-14(1)	-13(1)
O(1)	71(1)	44(1)	51(1)	5(1)	-31(1)	-32(1)
O(2)	33(1)	42(1)	74(1)	5(1)	-2(1)	-11(1)
O(6)	58(1)	64(1)	81(2)	20(1)	-17(1)	-38(1)
O(10)	68(1)	51(1)	42(1)	3(1)	-30(1)	-22(1)
O(11)	83(2)	62(1)	66(1)	28(1)	-20(1)	-40(1)
O(12)	75(1)	53(1)	44(1)	14(1)	-31(1)	-28(1)
N(1)	41(1)	36(1)	44(1)	-10(1)	-7(1)	-14(1)
C(1)	60(2)	59(2)	60(2)	-24(1)	3(1)	-31(1)
C(2)	36(1)	33(1)	56(2)	0(1)	-8(1)	-9(1)
C(3)	32(1)	36(1)	49(1)	9(1)	-18(1)	-9(1)
C(4)	37(1)	29(1)	34(1)	2(1)	-16(1)	-11(1)
C(5)	42(1)	32(1)	37(1)	2(1)	-11(1)	-14(1)
C(6)	46(1)	40(1)	47(2)	4(1)	-10(1)	-21(1)
C(7)	37(1)	53(2)	54(2)	8(1)	-18(1)	-20(1)
C(8)	31(1)	37(1)	42(1)	10(1)	-13(1)	-10(1)
C(9)	33(1)	27(1)	33(1)	3(1)	-13(1)	-10(1)
C(10)	41(1)	39(1)	37(1)	0(1)	-14(1)	-20(1)
C(11)	57(2)	37(1)	42(1)	4(1)	-15(1)	-17(1)
C(12)	104(3)	69(2)	57(2)	22(2)	-49(2)	-31(2)
C(13)	46(1)	38(1)	55(2)	0(1)	0(1)	-7(1)
C(14)	37(1)	26(1)	37(1)	2(1)	-10(1)	-13(1)
C(15)	41(1)	33(1)	41(1)	2(1)	-6(1)	-15(1)
C(16)	39(1)	32(1)	64(2)	7(1)	-9(1)	-11(1)
C(17)	61(2)	44(1)	59(2)	19(1)	-33(1)	-27(1)
C(18)	100(2)	49(2)	40(2)	7(1)	-24(2)	-35(2)
C(19)	75(2)	36(1)	38(1)	0(1)	-11(1)	-20(1)

Table S9. Hydrogen bonds for jb94 [Å and °].

D-H...A	d(D-H)	d(H...A)	d(D...A)	<(DHA)
O(6)-H(6)...O(11)	0.82	1.84	2.553(3)	145.3

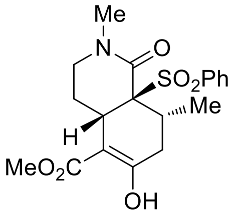

12b

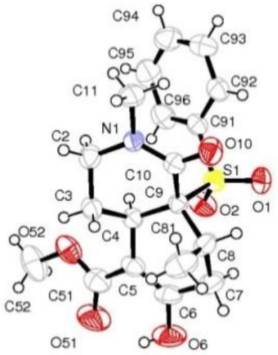

Table S10. Crystal data and structure refinement for compound **12b**.

Identification code	Jb96	
Empirical formula	C19 H23 N O6 S	
Formula weight	393.44	
Temperature	294(2) K	
Wavelength	0.71073 Å	
Crystal system	Orthorhombic	
Space group	P 21 21 21	
Unit cell dimensions	a = 8.256(2) Å	α= 90°.
	b = 11.5211(14) Å	β= 90°.
	c = 19.717(3) Å	γ = 90°.
Volume	1875.4(6) $Å^3$	
Z	4	
Density (calculated)	1.393 Mg/m^3	
Absorption coefficient	0.209 mm^{-1}	
F(000)	832	
Crystal size	0.39 x 0.21 x 0.21 mm^3	
Theta range for data collection	2.05 to 24.97°.	
Index ranges	-9<=h<=9, 0<=k<=13, 0<=l<=23	
Reflections collected	3689	
Independent reflections	3289 [R(int) = 0.0455]	
Completeness to theta = 24.97°	100.0 %	
Max. and min. transmission	0.9574 and 0.9230	
Refinement method	Full-matrix least-squares on F^2	
Data / restraints / parameters	3289 / 0 / 248	
Goodness-of-fit on F^2	0.971	
Final R indices [I>2sigma(I)]	R1 = 0.0492, wR2 = 0.0858	
R indices (all data)	R1 = 0.0944, wR2 = 0.0962	
Absolute structure parameter	-0.05(12)	
Largest diff. peak and hole	0.159 and -0.189 e.$Å^{-3}$	

Table S11. Atomic coordinates (x 10^4) and equivalent isotropic displacement parameters (Å^{2}x 10^3) for jb96. U(eq) is defined as one third of the trace of the orthogonalized U^{ij} tensor.

	x	y	z	U(eq)
S(1)	2860(1)	4807(1)	6406(1)	46(1)
O(1)	1504(3)	4042(2)	6520(1)	59(1)
O(2)	3254(3)	5645(2)	6923(1)	59(1)
O(6)	6615(4)	4060(3)	8239(1)	83(1)
O(10)	3428(3)	2795(2)	5397(1)	57(1)
O(51)	8625(4)	5592(3)	7879(1)	92(1)
O(52)	8414(3)	6362(3)	6852(2)	64(1)
N(1)	5486(3)	3898(3)	5030(1)	39(1)
C(2)	6916(4)	4605(3)	5151(2)	47(1)
C(3)	7538(4)	4440(3)	5864(2)	43(1)
C(4)	6200(3)	4734(3)	6374(2)	37(1)
C(5)	6791(4)	4738(3)	7103(2)	46(1)
C(6)	6124(5)	4054(4)	7591(2)	54(1)
C(7)	4768(5)	3239(4)	7467(2)	60(1)
C(8)	4649(5)	2872(3)	6730(2)	49(1)
C(9)	4712(4)	3936(3)	6249(2)	36(1)
C(10)	4529(4)	3481(3)	5516(2)	39(1)
C(11)	5115(4)	3627(4)	4322(2)	55(1)
C(51)	8005(5)	5566(4)	7319(2)	62(1)
C(52)	9650(5)	7218(4)	7051(2)	87(2)
C(81)	5969(5)	1973(3)	6574(2)	68(1)
C(91)	2523(4)	5564(3)	5638(2)	43(1)
C(92)	1552(4)	5088(4)	5138(2)	50(1)
C(93)	1327(5)	5689(4)	4537(2)	59(1)
C(94)	2086(6)	6726(4)	4432(2)	70(1)
C(95)	3048(5)	7190(4)	4921(3)	71(1)
C(96)	3239(5)	6624(4)	5543(2)	58(1)

Table S12. Bond lengths [Å] and angles [°] for jb96.

S(1)-O(1)	1.442(2)
S(1)-O(2)	1.442(2)
S(1)-C(91)	1.770(4)
S(1)-C(9)	1.855(3)
O(6)-C(6)	1.341(4)
O(10)-C(10)	1.227(4)
O(51)-C(51)	1.218(4)
O(52)-C(51)	1.343(5)
O(52)-C(52)	1.472(5)
N(1)-C(10)	1.332(4)
N(1)-C(2)	1.454(4)
N(1)-C(11)	1.462(4)
C(2)-C(3)	1.509(4)
C(3)-C(4)	1.531(4)
C(4)-C(5)	1.519(4)
C(4)-C(9)	1.554(4)
C(5)-C(6)	1.359(5)
C(5)-C(51)	1.448(5)
C(6)-C(7)	1.482(6)
C(7)-C(8)	1.517(5)
C(8)-C(81)	1.534(5)
C(8)-C(9)	1.550(5)
C(9)-C(10)	1.544(5)
C(91)-C(96)	1.370(5)
C(91)-C(92)	1.384(5)
C(92)-C(93)	1.385(5)
C(93)-C(94)	1.365(5)
C(94)-C(95)	1.358(6)
C(95)-C(96)	1.398(5)
O(1)-S(1)-O(2)	118.26(15)
O(1)-S(1)-C(91)	108.21(16)
O(2)-S(1)-C(91)	108.10(17)
O(1)-S(1)-C(9)	109.62(15)
O(2)-S(1)-C(9)	107.13(15)
C(91)-S(1)-C(9)	104.70(15)
C(51)-O(52)-C(52)	116.6(3)
C(10)-N(1)-C(2)	124.4(3)
C(10)-N(1)-C(11)	119.0(3)
C(2)-N(1)-C(11)	116.6(3)
N(1)-C(2)-C(3)	111.0(3)
C(2)-C(3)-C(4)	109.8(3)
C(5)-C(4)-C(3)	113.0(3)
C(5)-C(4)-C(9)	113.9(3)
C(3)-C(4)-C(9)	109.6(3)

Table S12. Cont.

C(6)-C(5)-C(51)	117.0(3)
C(6)-C(5)-C(4)	122.6(3)
C(51)-C(5)-C(4)	120.2(3)
O(6)-C(6)-C(5)	123.3(4)
O(6)-C(6)-C(7)	112.9(4)
C(5)-C(6)-C(7)	123.8(3)
C(6)-C(7)-C(8)	112.5(3)
C(7)-C(8)-C(81)	109.5(3)
C(7)-C(8)-C(9)	111.3(3)
C(81)-C(8)-C(9)	112.8(3)
C(10)-C(9)-C(8)	107.5(3)
C(10)-C(9)-C(4)	115.2(3)
C(8)-C(9)-C(4)	113.4(3)
C(10)-C(9)-S(1)	105.0(2)
C(8)-C(9)-S(1)	107.3(2)
C(4)-C(9)-S(1)	107.8(2)
O(10)-C(10)-N(1)	122.2(3)
O(10)-C(10)-C(9)	118.1(3)
N(1)-C(10)-C(9)	119.5(3)
O(51)-C(51)-O(52)	120.0(4)
O(51)-C(51)-C(5)	125.0(4)
O(52)-C(51)-C(5)	115.0(3)
C(96)-C(91)-C(92)	120.5(4)
C(96)-C(91)-S(1)	119.2(3)
C(92)-C(91)-S(1)	120.3(3)
C(91)-C(92)-C(93)	119.2(4)
C(94)-C(93)-C(92)	120.5(4)
C(95)-C(94)-C(93)	120.3(4)
C(94)-C(95)-C(96)	120.4(4)
C(91)-C(96)-C(95)	119.1(4)

Table S13. Anisotropic displacement parameters (Å^{2}x 10^3)for jb96. The anisotropic displacement factor exponent takes the form: -2 ^{2}[h^{2}a*^{2}U^{11} + ... + 2 h k a* b* U^{12}]

	U^{11}	U^{22}	U^{33}	U^{23}	U^{13}	U^{12}
S(1)	38(1)	59(1)	40(1)	-3(1)	4(1)	3(1)
O(1)	38(1)	81(2)	58(2)	6(2)	11(1)	-8(2)
O(2)	58(2)	74(2)	46(2)	-27(1)	-6(1)	11(2)
O(6)	95(3)	119(3)	34(2)	-2(2)	-14(2)	16(2)
O(10)	59(2)	64(2)	49(2)	-11(1)	3(1)	-20(2)
O(51)	91(2)	128(3)	56(2)	-20(2)	-30(2)	-6(2)
O(52)	53(2)	64(2)	75(2)	-24(2)	-13(2)	-6(2)
N(1)	38(2)	49(2)	30(2)	-3(2)	2(1)	-5(2)
C(2)	47(2)	54(3)	42(2)	-5(2)	5(2)	-5(2)
C(3)	37(2)	50(2)	43(2)	-5(2)	0(2)	1(2)
C(4)	36(2)	40(2)	36(2)	-4(2)	1(2)	2(2)
C(5)	43(2)	57(3)	37(2)	-11(2)	-6(2)	8(2)
C(6)	60(3)	71(3)	32(2)	-6(2)	-7(2)	17(2)
C(7)	71(3)	72(3)	38(2)	10(2)	8(2)	5(3)
C(8)	52(2)	50(2)	43(2)	10(2)	9(2)	0(2)
C(9)	37(2)	39(2)	31(2)	-1(2)	2(2)	1(2)
C(10)	38(2)	40(2)	39(2)	-3(2)	-1(2)	3(2)
C(11)	56(3)	75(3)	34(2)	-7(2)	-1(2)	0(2)
C(51)	62(3)	74(3)	50(3)	-16(2)	-4(2)	9(3)
C(52)	57(3)	90(4)	114(4)	-48(3)	-3(3)	-13(3)
C(81)	97(3)	49(3)	58(3)	7(2)	6(2)	14(2)
C(91)	35(2)	45(2)	48(2)	-2(2)	-2(2)	5(2)
C(92)	42(2)	55(3)	54(2)	-3(2)	-1(2)	2(2)
C(93)	61(3)	66(3)	51(3)	-5(2)	-12(2)	15(2)
C(94)	67(3)	72(3)	71(3)	24(3)	-5(3)	16(3)
C(95)	64(3)	45(3)	105(4)	19(3)	-9(3)	-1(3)
C(96)	51(3)	47(3)	77(3)	-2(2)	-9(2)	6(2)
